# Plant-derived compounds effectively inhibit the main protease of SARS-CoV-2: An in silico approach

**DOI:** 10.1371/journal.pone.0273341

**Published:** 2022-08-23

**Authors:** Shafi Mahmud, Shamima Afrose, Suvro Biswas, Abir Nagata, Gobindo Kumar Paul, Mohasana Akter Mita, Md. Robiul Hasan, Mst. Sharmin Sultana Shimu, Shahriar Zaman, Md. Salah Uddin, Md Sayeedul Islam, Md. Abu Saleh

**Affiliations:** 1 Division of Genome Sciences and Cancer, The John Curtin School of Medical Research, and The Shine-Dalgarno Centre for RNA Innovation, The Australian National University, Canberra, Australian Capital Territory, Australia; 2 Department of Genetic Engineering and Biotechnology, University of Rajshahi, Rajshahi, Bangladesh; 3 Microbiology Laboratory, Department of Genetic Engineering and Biotechnology, University of Rajshahi, Rajshahi, Bangladesh; 4 Department of Regenerative Dermatology, Graduate School of Medicine, Osaka University, Suita, Osaka, Japan; 5 Department of Biological Sciences, Graduate School of Science, Osaka University, Toyonaka, Osaka, Japan; University of Hail, SAUDI ARABIA

## Abstract

The current coronavirus disease 2019 (COVID-19) pandemic, caused by the coronavirus 2 (SARS-CoV-2), involves severe acute respiratory syndrome and poses unprecedented challenges to global health. Structure-based drug design techniques have been developed targeting the main protease of the SARS-CoV-2, responsible for viral replication and transcription, to rapidly identify effective inhibitors and therapeutic targets. Herein, we constructed a phytochemical dataset of 1154 compounds using deep literature mining and explored their potential to bind with and inhibit the main protease of SARS-CoV-2. The three most effective phytochemicals Cosmosiine, Pelargonidin-3-O-glucoside, and Cleomiscosin A had binding energies of -8.4, -8.4, and -8.2 kcal/mol, respectively, in the docking analysis. These molecules could bind to Gln189, Glu166, Cys145, His41, and Met165 residues on the active site of the targeted protein, leading to specific inhibition. The pharmacological characteristics and toxicity of these compounds, examined using absorption, distribution, metabolism, excretion, and toxicity (ADMET) analyses, revealed no carcinogenicity or toxicity. Furthermore, the complexes were simulated with molecular dynamics for 100 ns to calculate the root mean square deviation (RMSD), root mean square fluctuation (RMSF), radius of gyration (Rg), solvent-accessible surface area (SASA), and hydrogen profiles from the simulation trajectories. Our analysis validated the rigidity of the docked protein-ligand. Taken together, our computational study findings might help develop potential drugs to combat the main protease of the SARS-CoV-2 and help alleviate the severity of the pandemic.

## Introduction

Coronavirus disease 2019 (COVID-19) is caused by a new variant of the severe acute respiratory syndrome coronavirus 2 (SARS-CoV-2) that is 65–125 nm in diameter and originated from the Hubei Province of Wuhan in China [1]. A higher basic reproduction rate (R0 = 2–6.47) due to human-to-human transmission, either via respiratory droplets or via close contact with people, indicates the high transmissibility of the SARS-CoV-2 [2, 3]. According to the most recent information provided by the WHO, the outbreak has resulted in 536,590,224 confirmed cases and 6,316,655 deaths globally, as of June 20, 2022 (https://covid19.who.int/). SARS-CoV-2 is included in the Ortho-Coronaviridae subfamily of the Coronaviridae family and belongs to lineage B of the Betacoronavirus genus [4, 5]. The SARS-CoV-2 genome is the longest known RNA genome, composed of a single open reading frame and two un-translated regions (UTRs) with a 3′ poly-A tail and a 5′ cap structure [4, 6, 7]. Among the six open reading frames (ORFs), nonstructural proteins (nsp1–16) are encoded by the first ORF (ORF1) at the 5′ region. The remaining ORFs encode four structural and accessory proteins [8–10].

Mpro is a crucial enzyme that plays a key role in viral replication and transcription and is a promising therapeutic target against SARS-CoV-2 infection [11]. Being proteolytically autocleaved between nsp4 and nsp6, Mpro (nsp5) produces functional proteins from the polyproteins 1a (pp1a; 486 kDa) and pp1ab (790 kDa), which are overlapping replicase polyproteins that mediate viral replication and transcription [12, 13]. Nonstructural proteins, nsp1-nsp6, are produced by proteolytic cleavage between pp1a and pp1b along with the RNA-dependent RNA polymerase (RdRp) [14, 15]. The envelope (E), nucleocapsid (N), spike (S), and membrane (M) proteins, as well as accessory proteins, are encoded by the subgenomic RNAs processed from nsps [16, 17].

Main protease (Mpro) induces maturation of the polyproteins and is a prerequisite for nsps protein synthesis. Hence, the life cycle of the virus depends on the activity of Mpro. Pathogen progression and immune response within the host cell rely on the Mpro activity to produce the viral replicase enzyme [7, 16]. Therefore, viral replication and viral transcription require functional Mpro [15, 18]. Given the importance of its activity, Mpro has become a major focus for discovering novel and effective antiviral drugs [14, 19]. Inhibiting Mpro enzyme activity blocks viral replication by suppressing the production of infectious viral particles [13, 20]. Eventually, pathogenicity is mitigated as the viral particles fail to evade the host cell’s innate immunity [18, 21]. An Mpro homolog has not been identified in the human genome. All coronaviruses strictly conserve this enzyme, making it a potential therapeutic target for pharmacological inhibitors that could lead to the development of novel antiviral drugs [22].

Natural products, also known as secondary metabolites, have been the most significant sources of effective drug candidates [23–27]. Throughout history, phytochemicals have been used as remedies in the form of traditional medicines, potions, and oils. According to World Health Organization (WHO), 80% of the global population still uses conventional plant-derived medicines for basic health care, and 122 plant-derived pharmaceuticals have ethnopharmacological implications. For instance, “aspirin”, which is derived from a natural product, is a well-known anti-inflammatory agent [28]. Moreover, digitoxin, an active plant-derived component, improves heart contractibility strength. Furthermore, penicillin is the most well-known natural product derived from a fungus [28]. Lung cancer, acute leukemia, thyroid cancer, soft tissue and bone sarcomas, and both Hodgkins and non-Hodgkins lymphomas are all treated using doxorubicin [23]. Quinine has been used to treat malaria, fever, cancer, mouth and throat infections, and indigestion for millennia [28].

Natural components such as Pudilan Xiaoyan oral liquid, Xuebijing, Propolis, Tripterygium wilfordii, Artemisia annua, Glycyrrhiza glabra L., and Jinhua Qinggan granules exhibit plausible anti-SARS-CoV-2 effects according to an in silico prognosis [29]. Likewise, emodin, hesperidin, and chrysin have shown an S protein inhibiting efficacy that is analogous to chloroquine in tandem with hydroxychloroquine [30]. Phytochemicals such as lignan, quercetin, kaempferol, N-cis-feruloyltyramine, sugiol, jasmonic acid, putaminoxin D, 5,7-dimethoxyflavanone-4′-O-β-d-glucopyranoside, bonducellpin D, and caesalmin B have displayed anti-SARS-CoV-2 effect against Mpro target [31–33]. Moreover, karuquinone B, Lonicerae Japonicae Flos, and castanospermine, against S protein, in conjunction with cryptotanshinone, moupinamide, quercetin, coumaroyltyramine, kaempferol, tanshinone IIa, and N-cis-feruloyltyramine, against PLpro protein, have revealed inhibitory effects on SARS-CoV-2 [32, 34, 35]. Recently, a flavonol named quercetin, an FDA-approved compound included in anti-allergy and antioxidant medicines, has shown a promising inhibitory effect against SARS-CoV-2. As natural compounds and phytochemicals are efficiently processed at lower costs and have an impact on every stage of the interaction between the host and the virus, the quest for the most efficacious anti-SARS-CoV-2 therapeutic phytochemical is a worthwhile investigation to be performed both in silico and in vivo. Therefore, natural products and several phytochemicals, solely or in combination with traditional therapies, can be utilized to preclude and treat SARS-CoV-2 disease [29].

Computational drug detection schemes that include virtual screening techniques and molecular dynamics simulation proceedings are reliable high-throughput methods to identify plausible antiviral phytochemicals among a diverse array of repurposed phytochemical candidates. We hypothesized that in conjunction with molecular docking, a centralized virtual screening scheme can estimate the binding energy of compatible molecular interactions between the objective protein substrate and the potential ligand library. Moreover, computational methods identifying possible binding modes and binding site residues located in conserved motifs are plausible and scientifically reputable techniques to recognize repurposing phytochemicals. Such methods using drug-repurposing phytochemicals with antiviral activities consume less time than laborious conventional screening methods.

In low-income, economically backward, and underdeveloped countries, 17.8% of the people have received only one dose of the COVID-19 vaccine (https://ourworldindata.org/covid-vaccinations?country). The lack of vaccines and effective antiviral components and their limited efficacy necessitate the development of phytochemical-based eco-friendly phytopharmaceuticals against viral diseases that inhibit viral replication and penetration, while having manageable side effects and cost-effectiveness [36, 37]. Therefore, in this study, we aimed to identify effective inhibitors and therapeutic targets for blocking the function of SARS-CoV-2. We retrieved 1154 phytochemicals by literature review and docked them against Mpro using a molecular docking technique that calculates the binding affinities and modes between the target substrate (e.g. proteins) and numerous ligands, such as phytochemicals, in the shortest time possible.

## Material and methods

### Protein preparation

The Research Collaboratory for Structural Bioinformatics Protein Data Bank (RCSB PDB) Protein Data Bank was used to obtain the crystal structure of Mpro in SARS-CoV-2 (PDB-ID: 6LU7; resolution 2.16) [38]. The latest version of the Discovery Studio [39] and the PyMol software [40] package were used to deplete and capacitate the extracted protein structure through the computational process. First, we eliminated all the heteroatoms, water molecules, and inhibitors in Mpro. Then, a computational tool named SWISS PDB Viewer software [41] package was applied for further purification and energy depreciation in the presence of the Groningen Molecular Simulation (GROMOS) 43B1 force field. Alongside, the optimized crystal conformation of Mpro was scrutinized using the same software to recognize subsistent characteristics that include side-chain geometry, lacking hydrogen, and erroneous bond order.

### Ligand preparation

About 1154 phytochemicals were identified from various medicinal plants after an extensive and thorough literature survey (S1–S3 Tables). The three-dimensional structures of the procured phytochemicals that would be efficient ligand molecules were retrieved from the PubChem database [42]. For optimization and energy minimization of the ligand structure within about 2000 minimization steps, mmff94 force field [43] along with the steepest gradient algorithm was utilized.

#### Molecular docking study

Molecular docking through PyRx Virtual Screening Tools (Autodock Vina, v.1.2.0.) [44] was performed to determine the binding affinity between the phytochemicals and Mpro and to elucidate the binding pose, exhibiting every single feasible orientation and conformation for any specific ligands at the binding site of the Mpro and the phytochemicals. A suitable gradient method and a universal force field (UFF) were used to achieve structural optimization and energy minimization. The numerical value of the sum of the number of minimization steps programmed was about 2000. As compatible ligands and substrates are required to undergo docking in AutoDock Vina, Mpro was chosen as the macromolecule substrate, and phytochemicals were selected as the ligands. After precisely preparing the ligand and the substrate, a grid box in Autodock was imposed to identify the substrate-binding pocket representing the active site of the main protease. At the docking site of Mpro, the assigned grid box annotated the dimensions of X: 50.3334 Y: 67.2744 Z: 59.2586 Å, centered on X: 26.299 Y: 12.6039 Z: 58.9455 Å. As the ligand-flexible and protein-fixed docking was run, an anchored protease was identified, where every bond of the ligands was rotatable. After the completion of molecular docking, the highest docking energy signified the preeminent conformation. Based on the highest binding affinities and non-bonded interactions, the three top-most potential phytochemicals were marked for further investigation. Next, we used BIOVIA Discovery Studio and PyMol to explore the transcendent or docked conformations with the maximum docking energy by utilizing and assessing the visualized non-bonded interactions between the selected phytochemicals and the Mpro protein. Furthermore, the molecular docking approach with the formerly formulated grid was executed to dock the co-crystallized N3 ligand of 6LU7 by utilizing the ‘PyRx’ tool.

### Absorption, distribution, metabolism, excretion, and toxicity (ADMET) analysis

Indispensable online-based servers, such as the ADMET structure-activity relationship (admetSAR) [45], SwissADME [46], and pKCSM [47] databases, were used to analyze and evaluate the physicochemical descriptors in conjunction with the pharmacokinetic properties. The above-mentioned web servers require Canonical Simplified Molecular-Input Line-Entry System (SMILES) retrieved from the PubChem database that predicts medicinal chemistry compatibility of the selected plausible antiviral phytochemicals.

### Biological activities of the drug candidates

The online cheminformatics server "Molinspiration" (https://www.molinspiration.com/) was used to evaluate the biological activity of the screened phytochemicals. SMILES of these phytochemicals retrieved from the PubChem database were supplied as inputs to assess several biological activity parameters. This program applies sophisticated Bayesian statistics to assess a training set that consists of active structure and compares with the inactive molecules. Based on the investigation, a fragment-based model was created, and the bioactivity of each substructure fragment was estimated, adding up to the total activity contributions of the molecules’ fragments.

### Molecular dynamics simulation

The structural stability and constancy of the docked complex were precisely evaluated through the molecular dynamics simulation study. For molecular dynamics simulation, another scientific artificial reality application (YASARA) dynamics program [48] which used Assistant Model Building with Energy Refinement 14 (AMBER14) forcefield [49] was utilized. A cubic simulation cell was generated and extended for 20 Å at every part of the simulated protein-ligand complex. The docked complexes were initially cleaned and optimized with the hydrogen bond network orientations. The TIP3P water solvation model was used at 0.997 g/L-1, 25°C, and 1 atm, and energy minimization was performed using the steepest gradient algorithms with simulated annealing methods [50]. The physiological condition of the simulation cell was neutralized by the addition of 0.9% NaCl at pH 7.4 and 25°C. Long-range electrostatic interactions were calculated using the Particle Mesh Ewalds algorithm with a cut-off radius of 8 Å [51]. The time step of the simulation was set as 1.25 fs. The simulation trajectories were saved after every 100 ps, and the simulation was extended up to 100 ns periods. The trajectories were used to calculate the root mean square deviation (RMSD), root mean square fluctuation (RMSF), radius of gyration (Rg), solvent-accessible surface area (SASA), and hydrogen bonds [52–61].

## Results

### Molecular docking analysis

**Fig 1:**
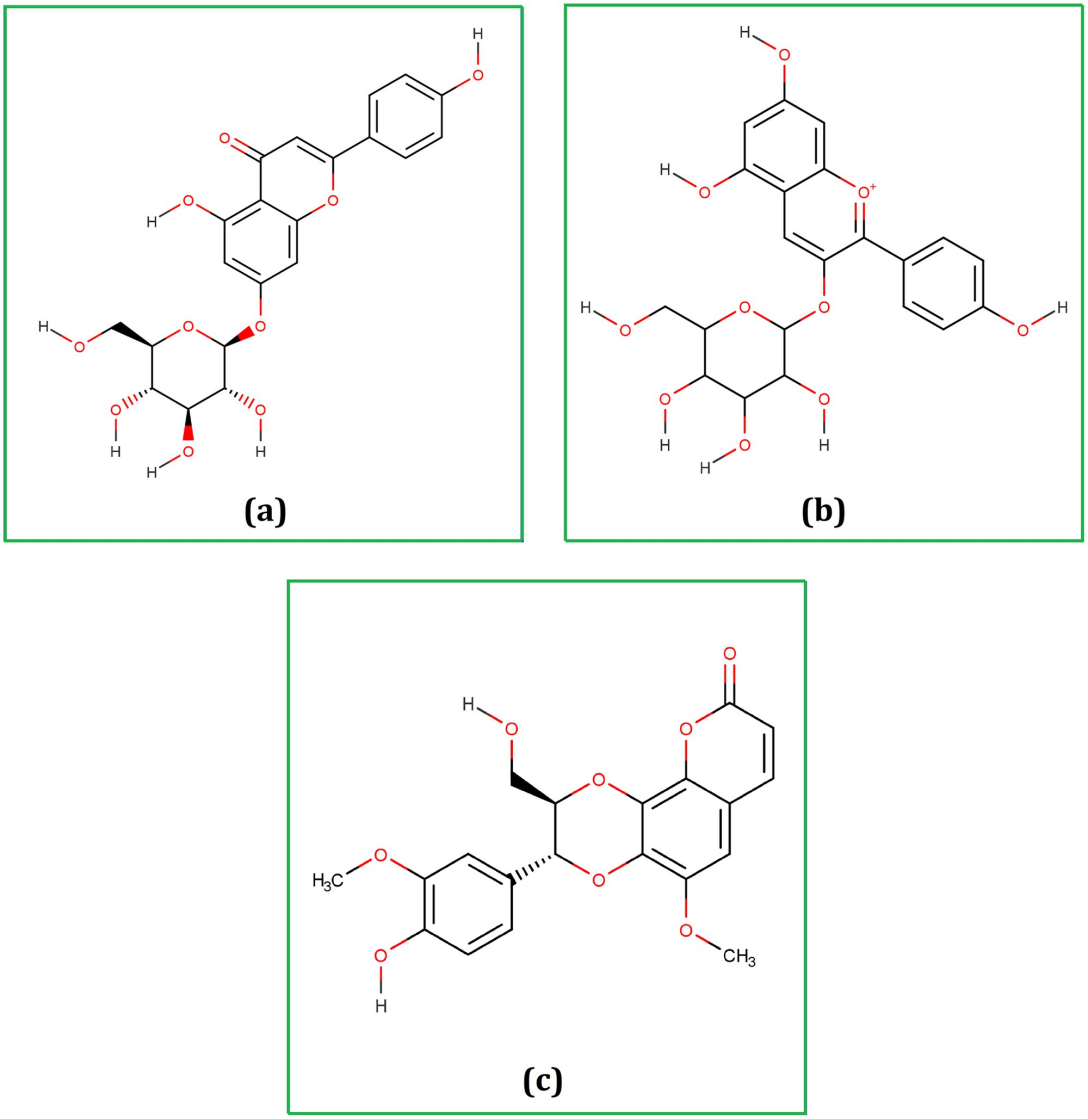
Chemical structure (2D) of Cosmosiine (a), Pelargonidin-3-O-glucoside (b), and Cleomiscosin A (c). The structures were drawn using the Marvin Sketch software.

The three phytochemicals identified among the 1154 in PubChem were cosmosiine (CID_5280704), pelargonidin-3-O-glucoside (CID_12302249), and cleomiscosin A (CID_442510) with binding affinities -8.4, -8.4, and -8.2 kcal/mol, respectively (Fig 1).

**Fig 2:**
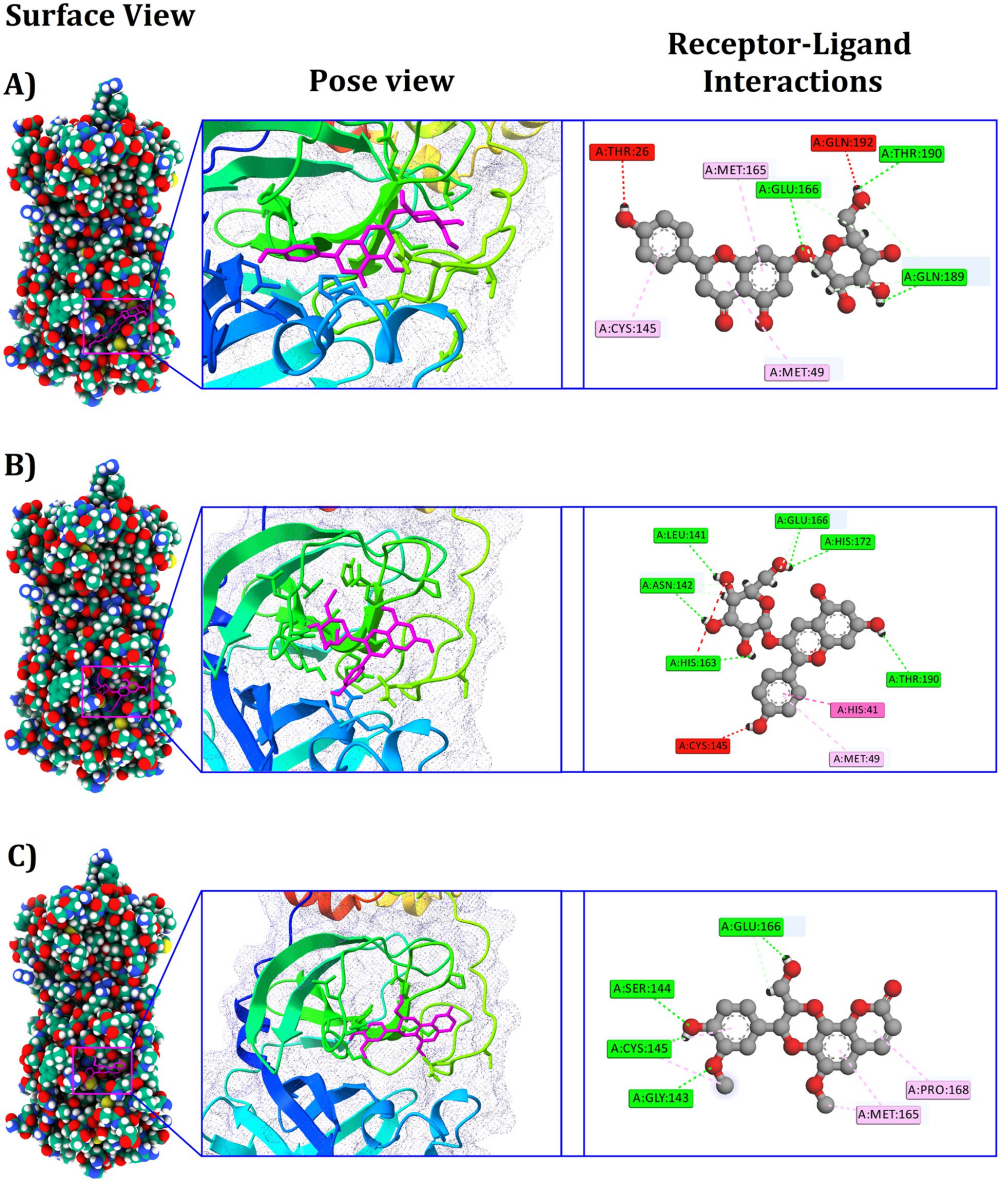
Illustration of the different binding modes of the selected compounds within the active and catalytic sites of the Mpro. The alphabetical orders indicate the respective complexes of Cosmosiine, Pelargonidin-3-O-glucoside, and Cleomiscosin A, respectively. Cosmosiine interacted with Thr190, Gln189, Glu166, Met165, Met49, and Cys145; pelargonidin-3-O-glucoside with Glu166, His172, Leu141, Asn142, His163, Thr190, His41, and MET49, and Cleomiscosin with Cys145, Glu166, Gly143, and Ser144, Met165, Pro168 residues.

Our investigation revealed that cosmosiine interacted with Mpro through three conventional hydrogen bonds at positions Thr190, Gln189, and Glu166, in addition to three pi-alkyl bonds formations at positions Met165, Met49, and Cys145 (Fig 2 and Table 1). On the other hand, pelargonidin-3-O-glucoside formed six conventional hydrogen bonds at Glu166, His172, Leu141, Asn142, His163, and Thr190, one pi-pi T-shaped bond at His41, and one pi-alkyl bond at MET49. Besides, a multiplex of cleomiscosin A and Mpro was equalized by four conventional hydrogen bonds at Cys145, Glu166, Gly143, and Ser144, one alkyl bond at Met165, in addition to a pi-alkyl bond at Pro168. After docking the co-crystallized N3 ligand with the prepared grid, the binding affinity was -7 kcal/mol, which was comparatively lower than the previously encountered binding affinity such as -8.4, -8.4, and -8.2 kcal/mol. Although the N3 co-crystallized ligand resided in the binding pocket of 6LU7, the lower binding affinity regarding N3 interaction reveals the preferentiality of 6LU7, substantiating virtual screening in tandem with docking analyses.

**Table 1:** Non-bonding interactions between SARS-CoV-2 Mpro and the top 3 phytochemical compounds.

Complex	PubChem CID	Binding affinity (kcal/mol)	Residues in contact	Interaction type	Distance in Ǻ
Cosmosiine	5280704	-8.4	THR190	Conventional Hydrogen Bond	2.47031
			GLN189	Conventional Hydrogen Bond	2.53715
			GLU166	Conventional Hydrogen Bond	2.78713
			MET165	pi-Alkyl	5.37772
			MET49	pi-Alkyl	4.53263
			CYS145	pi-Alkyl	5.00892
Pelargonidin-3-O-glucoside	12302249	-8.4	GLU166	Conventional Hydrogen Bond	2.18119
			HIS172	Conventional Hydrogen Bond	2.90534
			LEU141	Conventional Hydrogen Bond	2.37095
			ASN142	Conventional Hydrogen Bond	2.42628
			HIS163	Conventional Hydrogen Bond	2.39095
			THR190	Conventional Hydrogen Bond	2.58522
			HIS41	pi-pi T-shaped	5.16974
			MET49	pi-Alkyl	5.27065
Cleomiscosin A	442510	-8.2	CYS145	Conventional Hydrogen Bond	2.86763
			GLU166	Conventional Hydrogen Bond	2.04218
			GLY143	Conventional Hydrogen Bond	2.00045
			SER144	Conventional Hydrogen Bond	2.44246
			MET165	Alkyl	4.25281
			PRO168	pi-Alkyl	5.30755

### ADMET

Pharmacokinetics and toxicity, alongside other drug-like properties, of the screened top-most phytochemicals, were assessed for evaluating the potency and safety (Table 2). Moreover, several operative properties of these top phytochemicals, including carcinogenicity, hepatotoxicity, p-glycoprotein inhibition, cytochrome P450 (CYP) inhibition, and central nervous system (CNS) permeability, were evaluated. Here, CNS permeability refers to a compound’s ability to pass through the selectively semipermeable blood-brain barrier [47, 62]. It has been shown that the CNS permeability value should be greater than -2 to penetrate the central nervous system [63]. Neither toxicity nor carcinogenicity is subsistent within the selected phytochemicals. The molecular weight (MW) of the screened molecules, cosmosiine, pelargonidin-3-O-glucoside, and cleomiscosin A, were 432.4 g/mol, 433.4 g/mol, and 386.4 g/mol, respectively. These values indicate that these compounds adhere to the range of MW for the Lipinski rule of five [64]. Six donors and 10 acceptors of hydrogen bond in cosmosiine, seven donors and nine acceptors of hydrogen bond in pelargonidin-3-O-glucoside, and two donors and eight acceptors of hydrogen bond in cleomiscosin A were observed. Besides, each of these hit phytochemicals showed neither hepatotoxicity nor acute oral toxicity and followed the Lipinski rule of five.

**Table 2:** Pharmacological profile of the top three potential candidates derived from SwissADME, admetSAR, and pKCSM webservers.

Parameter	Cosmosiine	Pelargonidin-3-O-glucoside	Cleomiscosin A
Molecular weight	432.4 g/mol	433.4 g/mol	386.4 g/mol
H-Bond Acceptor	10	9	8
H-Bond Donor	6	7	2
CNS	-3.746	-3.628	-3.483
CYP2D6 substrate	No	No	No
CYP3A4 substrate	No	No	Yes
CYP1A2 inhibitor	No	No	No
CYP2C19 inhibitor	No	No	No
CYP2C9 inhibitor	No	No	No
CYP2D6 inhibitor	No	No	No
CYP3A4 inhibitor	No	No	No
Carcinogenicity	Non-carcinogenic	Non-carcinogenic	Non-carcinogenic
Hepatotoxicity	No	No	No
P-glycoprotein inhibitor	No	No	No
Acute Oral Toxicity	No	No	No
Lipinski rule of five	Yes	Yes	Yes

### Biological activities of the drug candidates

Several observations required meticulous evaluation to assess the biological activities of the top three plausible phytochemicals with significant antiviral repurposing therapeutic potential (Table 3). Cosmosiine showed the most preeminent GPCR ligand activity when compared with pelargonidin-3-O-glucoside and cleomiscosin A. In the case of ion channel inhibitor activity, pelargonidin-3-O-glucoside exhibited a comparatively lower value than cosmosiine and cleomiscosin A. As a kinase inhibitor, cleomiscosin A manifested an increased activity than pelargonidin-3-O-glucoside, whereas cosmosiine showed the most predominant activity. Cosmosiine also behaved as a more potent nuclear receptor-ligand compared to the other two phytochemicals. As a protease inhibitor, cosmosiine revealed the highest activity over both pelargonidin-3-O-glucoside and cleomiscosin A. All the screened phytochemicals exhibited promising enzyme inhibitor activities with cosmosiine having the highest activity value, and pelargonidin-3-O-glucoside and cleomiscosin showing almost similar activity values.

**Table 3:** Biological activities of the top screened phytochemicals.

Compounds	GPCR ligand	Ion channel inhibitor	Kinase inhibitor	Nuclear receptor-ligand	Protease inhibitor	Enzyme inhibitor
Cosmosiine	0.10	-0.01	0.14	0.31	0.02	0.43
Pelargonidin-3-O-glucoside	0.03	-0.03	-0.01	0.10	-0.04	0.25
Cleomiscosin A	-0.11	-0.18	-0.15	-0.07	-0.19	0.23

Note: Bioactivity score > 0 (biologically active); −5.0 < Bioactivity score < 0 (moderately active); Bioactivity score < 0 (biologically inactive).

### The molecular dynamics simulation study

**Fig 3:**
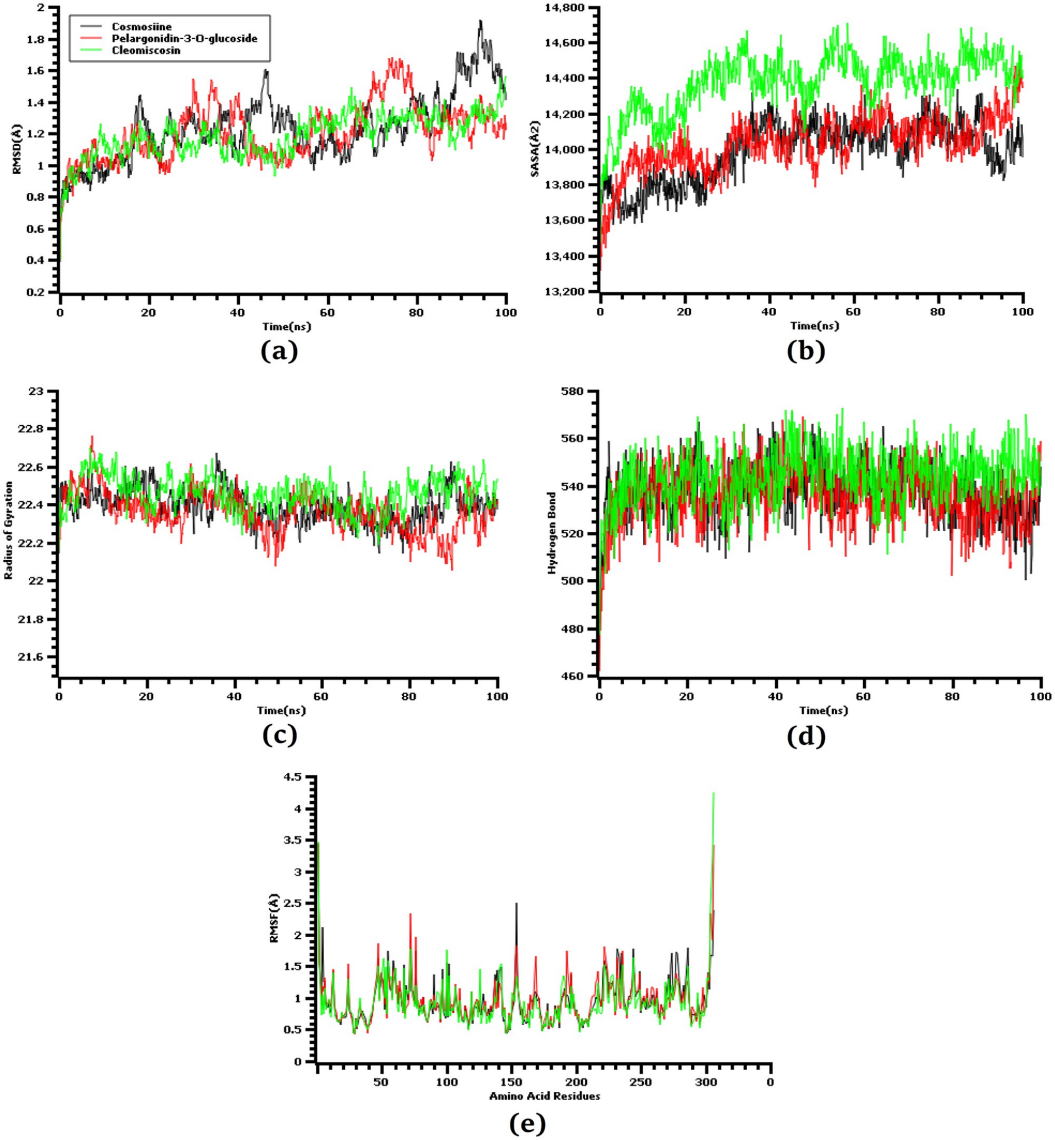
Time kinetics analysis of all the simulated systems. The alphabetical orders from (a) to (e) indicate RMSD analysis of the alpha carbon atoms (a), protein volume with expansion analysis (b), degree of rigidity and compactness analysis (c), hydrogen bond analysis (d), and flexibility analysis of amino acid residue (e).

The root mean square deviations of the simulations complexes were investigated to observe the variations and stability of the complexes. Fig 3(a) demonstrates how the top three docked complex, cosmosiine, pelargonidin-3-0-glucoside, cleomiscosin A showed an initial upward trend in RMSD. Cleomiscosin A maintained stability from the 20 ns time frame, and a lower degree of deviation in RMSD was observed for the entire simulation period. Pelargonidin-3-0-glucoside maintained a trend similar to that of cleomiscosin A for the maximum simulation periods. Pelargonidin-3-0-glucoside surpassed cleomiscosin A in RMSD in the 65–75-ns period, which might have ascended due to the higher instability of this complex. However, it quickly began to show a lower RMSD profile by maintaining a similar trend like cleomiscosin A, which also displayed a similar RMSD pattern like these two complexes in the entire simulation period. It had a slightly higher deviation at the last 85–90-ns time, although it did not significantly deviate.

The solvent-accessible surface area (SASA) of the simulated complexes was measured to determine how the protein volume changed, with a greater SASA profile indicating increased protein surface area and a lower SASA profile indicating truncation of the protein complexes. Fig 3(b) demonstrates that the cleomiscosin A complex had a higher SASA profile than the other two complexes in the whole simulation trajectories, which correlated with the complexes’ extensions. The expansion of the protein volume in SASA might have occurred due to the breakage of internal bonding. The other two complexes, with cosmosiine and pelargonidin-3-0-glucoside, had lower but constant SASA profiles in the whole simulation trajectories.

The radius of gyration of the simulation complexes is related to their labile nature, where a higher Rg profile indicates a more mobile nature of the complexes. Fig 3(c) shows that the three top complexes had a steady Rg profile in the entire trajectory and did not deviate significantly. The lower degree of fluctuations in the Rg descriptors for all three complexes correlated with the constant nature of the docked complexes.

We also analyzed the hydrogen bond from the trajectories as it had a vital role in determining the stability of the docked complexes. The top three screened complexes had regular hydrogen bonding patterns in the simulating environment, with cleomiscosin A having the most number of hydrogen bonds [Fig 3(d)].

The complexes’ root mean square fluctuations were analyzed to determine their flexibility across the amino acid residues [Fig 3(e)]. Majority of the amino acid residues had lower RMSF profile except for Ser1 (Helix strand), Gly2 (Helix strand), Asn72 (Helix strand), Tyr154 (Helix strand), Thr169 (Beta turn), Ala193 (Gamma turn), Arg222 (Gamma turn), Leu232 (Helix strand), Lys236 (Helix strand), Gln244 (Helix strand), Gly278 (Beta turn), Ser301 (Beta turn), Gly302 (Beta turn), Val303 (Beta turn), Thr304 (Beta turn), Phe305 (Beta turn), and Gln306 (Beta turn) residues.

The docked complexes were again analyzed after 100 ns simulation and explored for their binding interactions to evaluate their binding rigidity. The cosmosiine and the Mpro complexes were stabilized by five hydrogen bonds at Gln192, Gln189, Arg188, Thr26, and Thr190 positions, and three hydrophobic bonds (two pi-alkyl and one pi-sulfur bond) at Met49, Met165, and Cys145 positions (Table 4). The pelargonidin-3-O-glucoside and the Mpro complexes exhibited four hydrogen bonds at Glu166, His163, Ser144, and Leu141 after the 100 ns simulation time. Cleomiscosin A complexes had three hydrogen bonds at Gly143, Cys145, Asn142, and two alkyl bonds at Met165 and His41 positions.

**Table 4:** Non-bonded interactions of the docked complexes after the 100-ns simulation time, where H, PS, PA, and A represent hydrogen, pi-sulfur, pi-alkyl, and alkyl bonds, respectively.

Complex	Interacting Residues	Interaction Type	Distance (Å)
Cosmosiine	GLN192	H	1.91
	GLN189	H	1.56
	Arg188	H	2.50
	THR26	H	3.09
	THR190	H	2.47
	MET49	PS	4.35
	MET165	PA	4.47
	CYS145	PA	4.22
Pelargonidin-3-O-glucoside	GLU166	H	1.79
	HIS163	H	2.28
	SER144	H	2.87
	LEU141	H	2.91
Cleomiscosin A	GLY143	H	1.98
	CYS145	H	2.79
	ASN142	H	2.52
	MET165	A	4.34
	HIS41	A	4.21

## Discussion

According to our analysis and screening via molecular docking and dynamics investigations, three potent phytochemicals were selected, which showed higher binding affinity for Mpro at their active sites than the other compounds, which is essential for the targeted inhibition of Mpro. In the case of cosmosiine, after the 0 ns simulation time, we observed one interaction in Domain 1 (Met49), three interactions in Domain 2 (Cys145, Met165, and Glu166), and two interactions in the extended loop region (Gln189 and Thr190) connecting Domain 2 and Domain 3 (Fig 2 and Table 1). After the 100-ns simulation time, the cosmosiine and Mpro complex exhibited two interactions at Domain 1 (Thr26 and Met49), two interactions at Domain 2 (Met165 and Cys145), and four interactions at the more extended loop region (Gln192, Gln189, Arg188, and Thr190) which connects Domain 2 and 3. Cosmosiine can inhibit the cytotoxicity and the dysregulation associated with ACE2, IL1α, and TGFβ expressions induced by the recombinant spike protein of SARS-CoV-2 [65]. It has also been found to have several biological activities, such as induction of apoptosis, autophagy, and cell cycle arrest [66]. Previous studies have also suggested that cosmosiine inhibits cell migration in cervical cancer [67], insulin receptor phosphorylation, adiponectin secretion [68], inflammation, and oxidative stress [69]. Moreover, cosmosiine has potential anti-inflammatory and antibacterial properties [69].

In the case of pelargonidin-3-O-glucoside, two interactions were observed at Domain 1 (His41 and Met49), in addition to five interactions at Domain 2 (Glu166, His172, Leu141, Asn142, and His163) in tandem with one interaction in a longer loop region (Thr190) connecting Domain 2 and Domain 3 [13], after the 0 ns simulation time (Fig 2 and Table 1). The pelargonidin-3-O-glucoside and Mpro protease complex stabilization revealed four interactions at Domain 2 (Glu166, His163, Ser144, and Leu141) after the 100 ns simulation time. Pelargonidin-3-O-glucoside, a plant metabolite, has multiple pharmacological activities in cellular assays, including inhibition of peroxynitrate formation [70], interactions with erythrocytes and liposome membrane [71], improvement of cognitive impairment [72], nitrosative stress [73], and anthocyanin biosynthesis mechanisms [74].

According to a previous study by Abha et al., 2011, cleomiscosin A had a more significant binding capability with toll-like receptors (TLR-4), cluster of differentiation molecules (CDs), and inducible nitric oxide synthase (iNOS) protein [75]. There have been no studies on its interaction with the Mpro of SARS-CoV-2. In our study, cleomiscosin A displayed six interactions at Domain 2 (Cys145, Glu166, Gly143, Met165, Pro168, and Ser144) after the 0 ns simulation time (Fig 2 and Table 1). The stabilization of cleomiscosin A and Mpro complexes manifested one interaction at Domain 1 (HIS41), in conjunction with four interactions at Domain 2 (Gly143, Cys145, Asn142, and Met165) after the 100 ns of simulation time (Table 4). Cleomiscosin A also exhibits anti-inflammatory, analgesic, and antipyretic properties [76], as well as antihepatotoxic and anti-inflammatory properties [77, 78].

During the entire simulation period, diverse common interactions concerning the simulation sets for cosmosiine were identified, including Met49, Met165, Cys145, Gln189, and Thr190, which indicates the binding stability of the complex throughout the simulation time. Constant interactions were observed at Domain 1 (Met49), Domain 2 (Met165, and Cys145), and the linker loop region (Gln189 and Thr190). Our findings showed that cosmosiine predominantly interacts with the active site of the Mpro enzyme through residues including Met165, Cys145, Gln189, and Thr190, among which Cys145 is highly conserved among all coronaviruses [14]. Furthermore, pelargonidin-3-O-glucoside displayed distinct substantial interactions in the simulation epoch (during the 0 ns to 100 ns simulation time), including Glu166, His163, and Leu141. Precisely, the static interactions were remarkable at Domain 2 (Glu166, His163, and Leu141). However, pelargonidin-3-O-glucoside interacts with Mpro at the leading site through the residues Glu166, His163, and Leu141. Several stable interactions within the entire simulation time were identified, including Gly143, Cys145, and Met165 in the case of cleomiscosin A; stable interactions were observed at Domain 2 (Gly143, Cys145, and Met165). Prominently, cleomiscosin A interacts with Mpro enzyme’s active site through residues including Gly143, Cys145, and Met165. These common interactions throughout the simulation period reveal the binding rigidity or the binding stability of the complex.

The cosmosiine and pelargonidin-3-O-glucoside compounds exihibit beta-D-glucopyranosyl moeity where the pelargonidin-3-O-glucoside functions as a plant metabolite and cosmosiine has role in non-steroidial drug, metabolite and antibacterial agents. Both ligand molecules are beta glucosidage and functions in metabolisams. The cleomiscosin A is a heterocyclic compound which exihibit antiinflammatory activity. This compound is beta lactone and organic heterocyclic compounds.

Similar to our findings, numerous plant-derived compounds, such as curcumin, gartanin, robinetin [63], amentoflavone, gallocatechin gallate [79], chelidimerine, rutin, fumariline, catechin gallate, adlumidine, astragalin, somniferine [80], kaempferol, herbacetin, eugenol, 6-shogaol [81], triacontane, hexacosane, methyl linoleate, and methyl palmitoleate [52], formed similar binding patterns while docking with Main protease.

**Fig 4:**
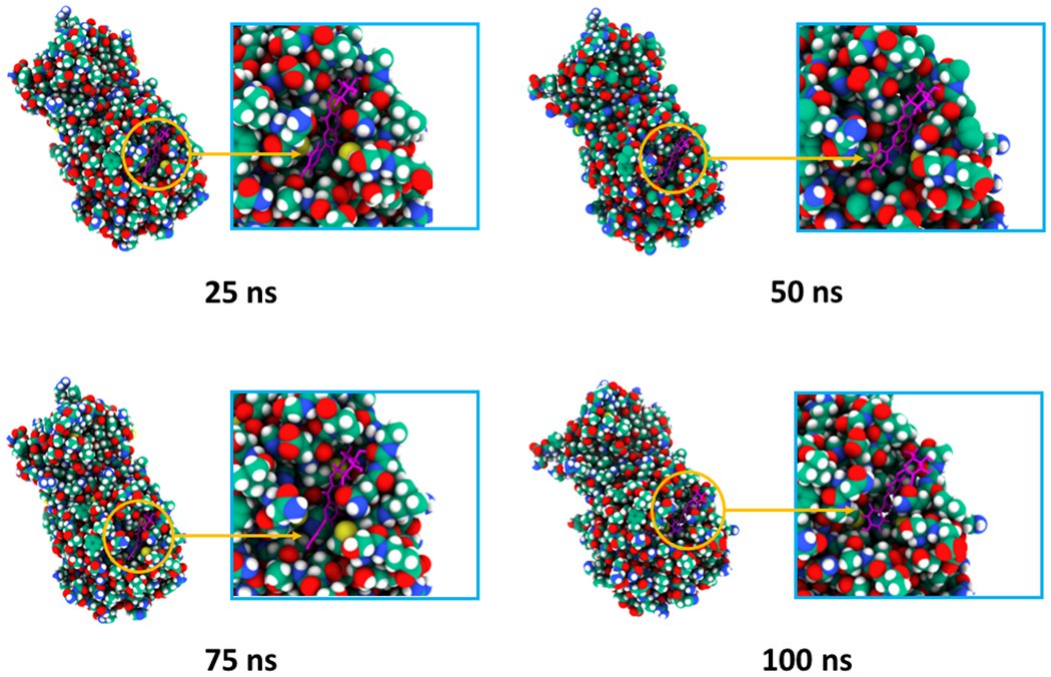
Surface view of the docked complexes in molecular dynamics simulation. The snapshots were acquired at 25, 50, 75, and 100 ns for the Cosmosiine and Mpro complex.

**Fig 5:**
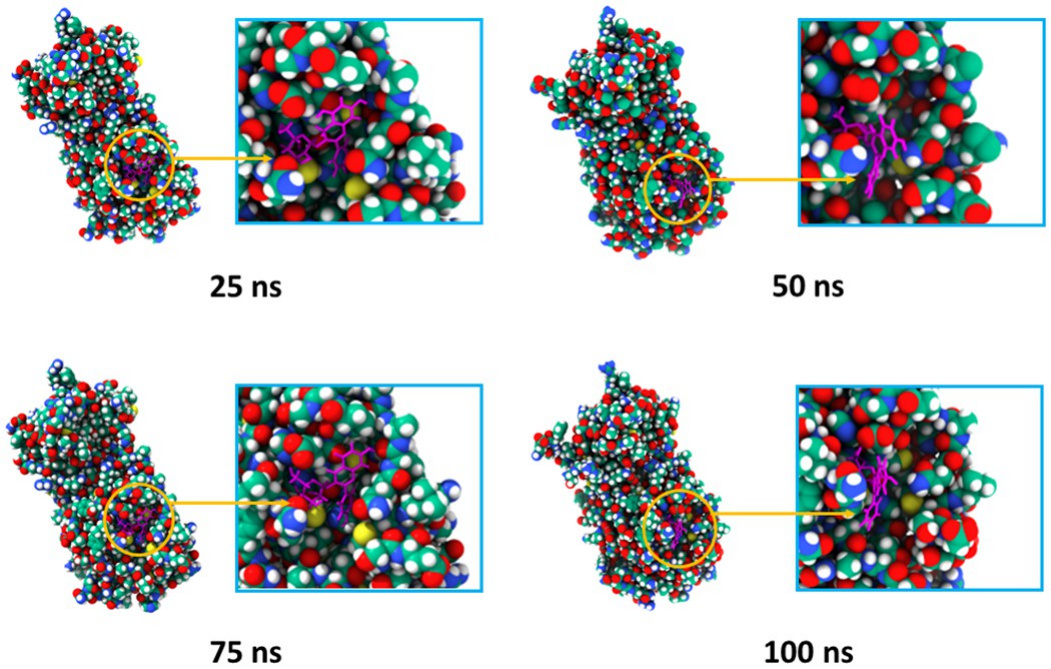
Surface view and binding pockets of the Pelargonidin-3-O-glucoside and Mpro complex, where snapshots at 25, 50, 75, and 100 ns were acquired.

**Fig 6:**
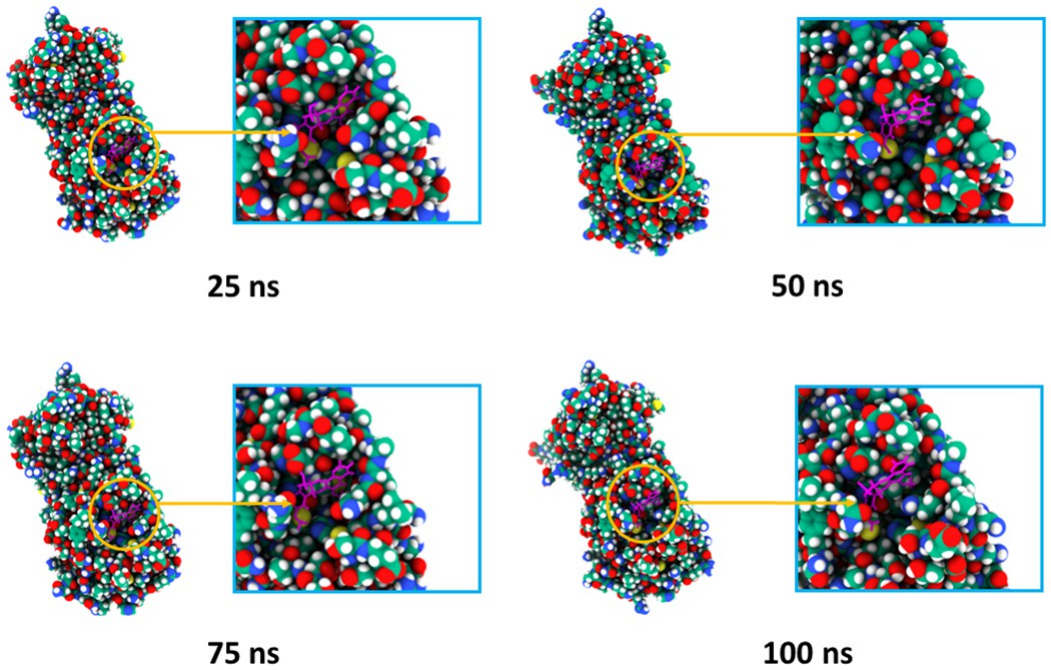
Surface view of the docked Cleomiscosin A and Mpro complex, where snapshots at 25, 50, 75, and 100 ns were acquired.

Molecular dynamics simulation of the docked complexes also revealed that the three compounds, cosmosiine, pelargonidin-3-O-glucoside, and cleomiscosin A, had a stable profile in several simulated trajectories, including RMSD, RMSF, SASA, Rg, and hydrogen bond studies. We also acquired images from various simulated trajectories, including at 25, 50, 75, and 100 ns, to investigate for changes in the binding sites or the binding rigidity at the binding pockets (Figs 4–6). In the simulated images, the docked positions of the three complexes were stable. The findings of the molecular docking and dynamics simulation studies suggest that these three compounds potentially inhibit the function of the SARS-targeted CoV-2 Mpro. However, these findings must be verified in a wet-lab setting.

## Conclusion

Using a computational method, we identified effective inhibitors of the SARS-CoV-2 main protease. The phytochemical library was tested against the main protease using molecular docking to identify the most potent lead compounds. In addition, three of the top ligand molecules in the library, namely, cosmosiine, pelargonidin-3-O-glucoside, and cleomiscosin A, were found to bind to the active site of the targeted protein. The binding orientation and the stiffness of the docked protein-ligand complexes were also determined using molecular dynamics simulation. Simulation descriptors, such as RMSD, RMSF, SASA, and Rg, and hydrogen bond descriptors aided in analyzing the inflexible character of the complexes in atomistic environments. The toxicity and carcinogenicity of the top compounds were thoroughly investigated using multiple computational tools, and a possibility of no adverse or unfavorable effects was observed. Because this study relies solely on computational mining, these findings require further in silico cross docking and in vitro investigations, including enzymatic assays, to confirm the computational findings.

## Supporting information

S1 Table Chemical name and pubchem CID of different phytochemicals retrived from different plants. (DOCX)

S2 Table Chemical name and pubchem CID of different phytochemicals retrived from different plants. (DOCX)

S3 Table Chemical name and pubchem CID of different phytochemicals retrived from different plants. (DOCX)

## References

[1] Ge H, Wang X, Yuan X, Xiao G, Wang C, Deng T, et al. The epidemiology and clinical information about COVID-19. Eur J Clin Microbiol Infect Dis. 2020;39: 1011–1019. doi: 10.1007/s10096-020-03874-z 32291542 PMC7154215

[2] Acter T, Uddin N, Das J, Akhter A, Choudhury TR, Kim S. Evolution of severe acute respiratory syndrome coronavirus 2 (SARS-CoV-2) as coronavirus disease 2019 (COVID-19) pandemic: A global health emergency. Sci Total Environ. 2020;730: 138996. doi: 10.1016/j.scitotenv.2020.138996 32371230 PMC7190497

[3] Ezzikouri S, Nourlil J, Benjelloun S, Kohara M, Tsukiyama-Kohara K. Coronavirus disease 2019—Historical context, virology, pathogenesis, immunotherapy, and vaccine development. Hum Vaccines Immunother. 2020;16: 2992–3000. doi: 10.1080/21645515.2020.1787068 32755425 PMC8641599

[4] Chan JFW, Kok KH, Zhu Z, Chu H, To KKW, Yuan S, et al. Genomic characterization of the 2019 novel human-pathogenic coronavirus isolated from a patient with atypical pneumonia after visiting Wuhan. Emerg Microbes Infect. 2020;9: 221–236. doi: 10.1080/22221751.2020.1719902 31987001 PMC7067204

[5] Benedetti F, Snyder GA, Giovanetti M, Angeletti S, Gallo RC, Ciccozzi M, et al. Emerging of a SARS-CoV-2 viral strain with a deletion in nsp1. J Transl Med. 2020;18: 4–9. doi: 10.1186/s12967-020-02507-5 32867854 PMC7457216

[6] Lu R, Zhao X, Li J, Niu P, Yang B, Wu H, et al. Genomic characterisation and epidemiology of 2019 novel coronavirus: implications for virus origins and receptor binding. Lancet. 2020;395: 565–574. doi: 10.1016/S0140-6736(20)30251-8 32007145 PMC7159086

[7] Zhou P, Lou Yang X, Wang XG, Hu B, Zhang L, Zhang W, et al. A pneumonia outbreak associated with a new coronavirus of probable bat origin. Nature. 2020;579: 270–273. doi: 10.1038/s41586-020-2012-7 32015507 PMC7095418

[8] Wu A, Peng Y, Huang B, Ding X, Wang X, Niu P, et al. Genome Composition and Divergence of the Novel Coronavirus (2019-nCoV) Originating in China. Cell Host Microbe. 2020;27: 325–328. doi: 10.1016/j.chom.2020.02.001 32035028 PMC7154514

[9] Yang Y, Xiao Z, Ye K, He X, Sun B, Qin Z, et al. SARS-CoV-2: characteristics and current advances in research. Virol J. 2020;17: 1–17. doi: 10.1186/s12985-020-01369-z 32727485 PMC7387805

[10] Ugurel OM, Ata O, Turgut-Balik D. An updated analysis of variations in SARS-CoV-2 genome. Turkish J Biol. 2020;44: 157–167. doi: 10.3906/biy-2005-111 32595352 PMC7314508

[11] Yang H, Yang M, Ding Y, Liu Y, Lou Z, Zhou Z, et al. The crystal structures of severe acute respiratory syndrome virus main protease and its complex with an inhibitor. Proc Natl Acad Sci U S A. 2003;100: 13190–13195. doi: 10.1073/pnas.1835675100 14585926 PMC263746

[12] X B., K X. Activation and maturation of SARS-CoV main protease. Protein Cell. 2011;2: 282–290. doi: 10.1007/s13238-011-1034-1 21533772 PMC4875205

[13] Ullrich S, Nitsche C. The SARS-CoV-2 main protease as drug target. Bioorganic Med Chem Lett. 2020;30: 127377. doi: 10.1016/j.bmcl.2020.127377 32738988 PMC7331567

[14] Khan SA, Zia K, Ashraf S, Uddin R, Ul-Haq Z. Identification of chymotrypsin-like protease inhibitors of SARS-CoV-2 via integrated computational approach. J Biomol Struct Dyn. 2020;0: 1–10. doi: 10.1080/07391102.2020.1751298 32238094

[15] Kuo CJ, Chi YH, Hsu JTA, Liang PH. Characterization of SARS main protease and inhibitor assay using a fluorogenic substrate. Biochem Biophys Res Commun. 2004;318: 862–867. doi: 10.1016/j.bbrc.2004.04.098 15147951 PMC7134607

[16] Dai W, Zhang B, Jiang XM, Su H, Li J, Zhao Y, et al. Structure-based design of antiviral drug candidates targeting the SARS-CoV-2 main protease. Science (80-). 2020;368: 1331–1335. doi: 10.1126/science.abb4489 32321856 PMC7179937

[17] Ren Z, Yan L, Zhang N, Guo Y, Yang C, Lou Z, et al. The newly emerged SARS-Like coronavirus HCoV-EMC also has an “Achilles” heel": Current effective inhibitor targeting a 3C-like protease”. Protein Cell. 2013;4: 248–250. doi: 10.1007/s13238-013-2841-3 23549610 PMC4875521

[18] Anand K, Ziebuhr J, Wadhwani P, Mesters JR, Hilgenfeld R. Coronavirus main proteinase (3CLpro) Structure: Basis for design of anti-SARS drugs. Science (80-). 2003;300: 1763–1767. doi: 10.1126/science.1085658 12746549

[19] Yang H, Xie W, Xue X, Yang K, Ma J, Liang W, et al. Design of wide-spectrum inhibitors targeting coronavirus main proteases. PLoS Biol. 2005;3. doi: 10.1371/journal.pbio.0030324 16128623 PMC1197287

[20] Serseg T, Benarous K, Yousfi M. Hispidin and Lepidine E: Two natural compounds and folic acid as potential inhibitors of 2019-novel coronavirus main protease (2019-nCoVMpro), molecular docking and SAR study. arXiv. 2020. 10.2174/1573409916666200422075440 32321407

[21] Grum-Tokars V, Ratia K, Begaye A, Baker SC, Mesecar AD. Evaluating the 3C-like protease activity of SARS-Coronavirus: Recommendations for standardized assays for drug discovery. Virus Res. 2008;133: 63–73. doi: 10.1016/j.virusres.2007.02.015 17397958 PMC4036818

[22] Xiantian X, Ping C, Jingfang W, Jiannan F, Hui Z, Xuan L, et al. Evolution of the novel coronavirus from the ongoing Wuhan outbreak and modeling of its spike protein for risk of human transmission. Sci CHINA Life Sci. 2020;63: 457–460. doi: 10.1007/s11427-020-1637-5 32009228 PMC7089049

[23] Haefner B. Drugs from the deep: Marine natural products as drug candidates. Drug Discov Today. 2003;8: 536–544. doi: 10.1016/s1359-6446(03)02713-2 12821301

[24] Cragg GM, Newman DJ. Biodiversity: A continuing source of novel drug leads. Pure Appl Chem. 2005;77: 7–24. doi: 10.1351/pac200577010007

[25] Rey-Ladino J, Ross AG, Cripps AW, McManus DP, Quinn R. Natural products and the search for novel vaccine adjuvants. Vaccine. 2011;29: 6464–6471. doi: 10.1016/j.vaccine.2011.07.041 21787827

[26] Mishra BB, Tiwari VK. Natural products: An evolving role in future drug discovery. Eur J Med Chem. 2011;46: 4769–4807. doi: 10.1016/j.ejmech.2011.07.057 21889825

[27] Graupner P. The Role of Natural Product Chemistry in Agriculture. Planta Med. 2013;79: 2141–2153. doi: 10.1055/s-0033-1348516

[28] Dias DA, Urban S, Roessner U. A Historical overview of natural products in drug discovery. Metabolites. 2012;2: 303–336. doi: 10.3390/metabo2020303 24957513 PMC3901206

[29] Merarchi M, Dudha N, Das BC, Garg M. Natural products and phytochemicals as potential anti-SARS-CoV-2 drugs. Phyther Res. 2021;35: 5384–5396. doi: 10.1002/ptr.7151 34132421 PMC8441929

[30] Basu A, Sarkar A, Maulik U. Molecular docking study of potential phytochemicals and their effects on the complex of SARS-CoV2 spike protein and human ACE2. Sci Rep. 2020;10: 1–15. doi: 10.1038/s41598-020-74715-4 33077836 PMC7573581

[31] Padhi S, Masi M, Chourasia R, Rajashekar Y, Rai AK, Evidente A. ADMET profile and virtual screening of plant and microbial natural metabolites as SARS-CoV-2 S1 glycoprotein receptor binding domain and main protease inhibitors. Eur J Pharmacol. 2020;890: 173648. doi: 10.1016/j.ejphar.2020.173648 33069672 PMC7561576

[32] hai Zhang D, lun Wu K, Zhang X, qiong Deng S, Peng B. In silico screening of Chinese herbal medicines with the potential to directly inhibit 2019 novel coronavirus. J Integr Med. 2020;18: 152–158. doi: 10.1016/j.joim.2020.02.005 32113846 PMC7102521

[33] Gurung AB, Ali MA, Lee J, Farah MA, Al-Anazi KM. Unravelling lead antiviral phytochemicals for the inhibition of SARS-CoV-2 Mpro enzyme through in silico approach. Life Sci. 2020;255: 117831. doi: 10.1016/j.lfs.2020.117831 32450166 PMC7243810

[34] Al-Sehemi AG, Olotu FA, Dev S, Pannipara M, Soliman ME, Carradori S, et al. Natural Products Database Screening for the Discovery of Naturally Occurring SARS-Cov-2 Spike Glycoprotein Blockers. ChemistrySelect. 2020;5: 13309–13317. doi: 10.1002/slct.202003349 33363254 PMC7753608

[35] Niu M, Wang R-L, Wang Z-X, Zhang P, Bai Z-F, Jing J, et al. [Rapid establishment of traditional Chinese medicine prevention and treatment of 2019-nCoV based on clinical experience and molecular docking]. Zhongguo Zhong Yao Za Zhi. 2020;45: 1213–1218. doi: 10.19540/j.cnki.cjcmm.20200206.501 32281327

[36] Attia YA, Alagawany MM, Farag MR, Alkhatib FM, Khafaga AF, Abdel-Moneim AME, et al. Phytogenic Products and Phytochemicals as a Candidate Strategy to Improve Tolerance to Coronavirus. Front Vet Sci. 2020;7: 1–18. doi: 10.3389/fvets.2020.573159 33195565 PMC7606864

[37] Ben-Shabat S, Yarmolinsky L, Porat D, Dahan A. Antiviral effect of phytochemicals from medicinal plants: Applications and drug delivery strategies. Drug Deliv Transl Res. 2020;10: 354–367. doi: 10.1007/s13346-019-00691-6 31788762 PMC7097340

[38] Rose PW, Prlić A, Altunkaya A, Bi C, Bradley AR, Christie CH, et al. The RCSB protein data bank: Integrative view of protein, gene and 3D structural information. Nucleic Acids Res. 2017;45: D271–D281. doi: 10.1093/nar/gkw1000 27794042 PMC5210513

[39] Studio D. version 2.5. Accelrys Inc San Diego, CA, USA. 2009.

[40] Delano W. The PyMOL Molecular Graphics System. 2002.

[41] Kaplan WLT. Software review Swiss-PDB Viewer (Deep View). Brief Bioinform. 2001;2: 195–197. 11465736 10.1093/bib/2.2.195

[42] Kim S, Thiessen PA, Bolton EE, Chen J, Fu G, Gindulyte A, et al. PubChem substance and compound databases. Nucleic Acids Res. 2016;44: D1202–D1213. doi: 10.1093/nar/gkv951 26400175 PMC4702940

[43] Halgren TA. Performance of MMFF94*. J Comput Chem. 1996;17: 490–519. Available: http://journals.wiley.com/jcc

[44] Trott O, Olson AJ. AutoDock Vina: Improving the speed and accuracy of docking with a new scoring function, efficient optimization, and multithreading. J Comput Chem. 2009. doi: 10.1002/jcc.21334 19499576 PMC3041641

[45] Cheng F, Li W, Zhou Y, Shen J, Wu Z, Liu G, et al. AdmetSAR: A comprehensive source and free tool for assessment of chemical ADMET properties. J Chem Inf Model. 2012;52: 3099–3105. doi: 10.1021/ci300367a 23092397

[46] Daina A, Michielin O, Zoete V. SwissADME: A free web tool to evaluate pharmacokinetics, drug-likeness and medicinal chemistry friendliness of small molecules. Sci Rep. 2017;7: 1–13. doi: 10.1038/srep42717 28256516 PMC5335600

[47] Pires DEV, Blundell TL, Ascher DB. pkCSM: Predicting small-molecule pharmacokinetic and toxicity properties using graph-based signatures. J Med Chem. 2015;58: 4066–4072. doi: 10.1021/acs.jmedchem.5b00104 25860834 PMC4434528

[48] Land H, Humble MS. YASARA: A tool to obtain structural guidance in biocatalytic investigations. Methods Mol Biol. 2018;1685: 43–67. doi: 10.1007/978-1-4939-7366-8_4 29086303

[49] Case DA, Cheatham TE, Darden T, Gohlke H, Luo R, Merz KM, et al. The Amber biomolecular simulation programs. J Comput Chem. 2005;26: 1668–1688. doi: 10.1002/jcc.20290 16200636 PMC1989667

[50] Krieger E, Vriend G. New ways to boost molecular dynamics simulations. J Comput Chem. 2015;36: 996–1007. doi: 10.1002/jcc.23899 25824339 PMC6680170

[51] Krieger E, Nielsen JE, Spronk CAEM, Vriend G. Fast empirical p K a prediction by Ewald summation. 2006;25: 481–486. doi: 10.1016/j.jmgm.2006.02.009 16644253

[52] Mahmud S, Paul GK, Afroze M, Islam S, Gupt SBR, Razu MH, et al. Efficacy of phytochemicals derived from avicennia officinalis for the management of covid-19: A combined in silico and biochemical study. Molecules. 2021;26. doi: 10.3390/molecules26082210 33921289 PMC8070553

[53] Mahmud S, Biswas S, Paul GK, Mita MA, Promi MM, Afrose S, et al. Plant-based phytochemical screening by targeting main protease of sars-cov-2 to design effective potent inhibitors. Biology (Basel). 2021;10. doi: 10.3390/biology10070589 34206970 PMC8301192

[54] Mahmud S, Paul GK, Biswas S, Afrose S, Mita MA, Hasan MR, et al. Prospective Role of Peptide-Based Antiviral Therapy Against the Main Protease of SARS-CoV-2. Front Mol Biosci. 2021;8. doi: 10.3389/fmolb.2021.628585 34041263 PMC8142691

[55] Mahfuz AMU., Khan A, Biswas S, Afrose S, Mahmud S, Mohammed Bahadur N, et al. In search of inhibitors of anti-cancer drug target Fibroblast Growth Factor Receptors: insights from virtual screening, molecular docking, and molecular dynamics study. Arab J Chem. 2022;15: 103882. doi: 10.1016/j.arabjc.2022.103882

[56] Mahmud S, Hasan MR, Biswas S, Paul GK, Afrose S, Mita MA, et al. Screening of Potent Phytochemical Inhibitors Against SARS-CoV-2 Main Protease: An Integrative Computational Approach. Front Bioinforma. 2021;1: 1–15. doi: 10.3389/fbinf.2021.717141 PMC9581031 36303755

[57] Kumar Paul G, Mahmud S, Aldahish AA, Afroze M, Biswas S, Briti Ray Gupta S, et al. Computational screening and biochemical analysis of Pistacia integerrima and Pandanus odorifer plants to find effective inhibitors against Receptor-Binding domain (RBD) of the spike protein of SARS-Cov-2. Arab J Chem. 2022;15: 103600. doi: 10.1016/j.arabjc.2021.103600 34909068 PMC8632739

[58] Mahmud S, Rafi O, Paul GK, Promi MM, Sharmin M, Shimu S, et al. Designing a multi—epitope vaccine candidate to combat MERS—CoV by employing an immunoinformatics approach. Sci Rep. 2021; 1–21. doi: 10.1038/s41598-021-92176-1 34326355 PMC8322212

[59] Biswas S, Mahmud S, Mita MA, Afrose S, Hasan MR, Sultana Shimu MS, et al. Molecular Docking and Dynamics Studies to Explore Effective Inhibitory Peptides Against the Spike Receptor Binding Domain of SARS-CoV-2. Front Mol Biosci. 2022;8: 1–10. doi: 10.3389/fmolb.2021.791642 35187069 PMC8851422

[60] Antiviral peptides against the main protease of SARS-CoV_2: A molecular docking and dynamics study. 10.1016/j.arabjc.2021.103315 PMC8277949 34909064

[61] Molecular docking and dynamics study to explore phytochemical ligand molecules against the main protease of SARS-CoV-2 from extensive phytochemical datasets. 10.1080/17512433.2021.1959318 34301158

[62] Bellettato CM, Scarpa M. Possible strategies to cross the blood–brain barrier. Ital J Pediatr. 2018;44. doi: 10.1186/s13052-018-0563-0 30442184 PMC6238258

[63] Mahmud S, Uddin MAR, Paul GK, Shimu MSS, Islam S, Rahman E, et al. Virtual screening and molecular dynamics simulation study of plant-derived compounds to identify potential inhibitors of main protease from SARS-CoV-2. Brief Bioinform. 2021.; 22: 1402–1414. doi: 10.1093/bib/bbaa428 33517367 PMC7929365

[64] Lipinski CA. Lead- and drug-like compounds: The rule-of-five revolution. Drug Discov Today Technol. 2004;1: 337–341. doi: 10.1016/j.ddtec.2004.11.007 24981612

[65] Luo Grace, Zhu Wei. Apigetrin inhibits cytotoxicity and dysregulation of ACE2, IL1α and TGFβ expression induced by recombinant spike protein of SARS-CoV-2. World J Adv Res Rev. 2021;9: 144–154. doi: 10.30574/wjarr.2021.9.2.0035

[66] Kim SM, Vetrivel P, Ha SE, Kim HH, Kim JA, Kim GS. Apigetrin induces extrinsic apoptosis, autophagy and G2/M phase cell cycle arrest through PI3K/AKT/mTOR pathway in AGS human gastric cancer cell. J Nutr Biochem. 2020;83: 108427. doi: 10.1016/j.jnutbio.2020.108427 32559585

[67] Liu MM, Ma RH, Ni ZJ, Thakur K, Cespedes-Acuña CL, Jiang L, et al. Apigenin 7-O-glucoside promotes cell apoptosis through the PTEN/PI3K/AKT pathway and inhibits cell migration in cervical cancer HeLa cells. Food Chem Toxicol. 2020;146. doi: 10.1016/j.fct.2020.111843 33152472

[68] Tzeng YM, Rao YK, Lee MJ, Chen K, Lee YC, Wu WS. Insulin-mimetic action of rhoifolin and cosmosiin isolated from citrus grandis (L.) osbeck leaves: Enhanced adiponectin secretion and insulin receptor phosphorylation in 3T3-L1 cells. Evidence-based Complement Altern Med. 2011;2011. doi: 10.1093/ecam/nep204 20008903 PMC3152991

[69] Guo H, Li M, Xu LJ. Apigetrin treatment attenuates LPS-induced acute otitis media though suppressing inflammation and oxidative stress. Biomed Pharmacother. 2019;109: 1978–1987. doi: 10.1016/j.biopha.2018.07.022 30551453

[70] Paixão J, Dinis TCP, Almeida LM. Malvidin-3-glucoside protects endothelial cells up-regulating endothelial NO synthase and inhibiting peroxynitrite-induced NF-kB activation. Chem Biol Interact. 2012;199: 192–200. doi: 10.1016/j.cbi.2012.08.013 22959858

[71] Bonarska-Kujawa D, Pruchnik H, Kleszczyńska H. Interaction of selected anthocyanins with erythrocytes and liposome membranes. Cell Mol Biol Lett. 2012;17: 289–308. doi: 10.2478/s11658-012-0010-y 22396139 PMC6275648

[72] Ridzwan N, Jumli MN, Baig AA, Rohin MAK. Pomegranate-derived anthocyanin regulates MORs-cAMP/CREB-BDNF pathways in opioid-dependent models and improves cognitive impairments. J Ayurveda Integr Med. 2020;11: 478–488. doi: 10.1016/j.jaim.2019.12.001 32430240 PMC7772514

[73] Winter AN, Ross EK, Khatter S, Miller K, Linseman DA. Chemical basis for the disparate neuroprotective effects of the anthocyanins, callistephin and kuromanin, against nitrosative stress. Free Radic Biol Med. 2017;103: 23–34. doi: 10.1016/j.freeradbiomed.2016.12.012 27986528

[74] Huang G, Huang G, Zeng Y, Wei L, Yao Y, Dai J, et al. Comparative transcriptome analysis of mulberry reveals anthocyanin biosynthesis mechanisms in black (Morus atropurpurea Roxb.) and white (Morus alba L.) fruit genotypes. BMC Plant Biol. 2020;20: 1–12. doi: 10.1186/s12870-020-02486-1 32552771 PMC7301479

[75] Meena A, Yadav DK, Srivastava A, Khan F, Chanda D, Chattopadhyay SK. In Silico Exploration of Anti-Inflammatory Activity of Natural Coumarinolignoids. Chem Biol Drug Des. 2011;78: 567–579. doi: 10.1111/j.1747-0285.2011.01173.x 21736704

[76] Begum S, Saxena B, Goyal M, Ranjan R, Joshi VB, Rao CV., et al. Study of anti-inflammatory, analgesic and antipyretic activities of seeds of Hyoscyamus niger and isolation of a new coumarinolignan. Fitoterapia. 2010;81: 178–184. doi: 10.1016/j.fitote.2009.08.024 19720117

[77] Chattopadhyay SK, Kumar S, Kaur R, Tandon S, Rane S. Identification and quantification of two antihepatotoxic coumarinolignoids cleomiscosin A and cleomiscosin B in the seeds of Cleome viscosa using liquid chromatography-tandem mass spectrometry. Biomed Chromatogr. 2009;23: 340–356. doi: 10.1002/bmc.1121 18800331

[78] Sharma S, Chattopadhyay SK, Trivedi P, Bawankule DU. Synthesis and anti-inflammatory activity of derivatives of coumarino-lignoid, cleomiscosin A and its methyl ether. Eur J Med Chem. 2010;45: 5150–5156. doi: 10.1016/j.ejmech.2010.08.027 20813432

[79] Swargiary A, Mahmud S, Saleh MA. Screening of phytochemicals as potent inhibitor of 3-chymotrypsin and papain-like proteases of SARS-CoV2: an in silico approach to combat COVID-19. J Biomol Struct Dyn. 2020;0: 1–15. doi: 10.1080/07391102.2020.1835729 33089730 PMC7594184

[80] Mousavi SS, Karami A, Haghighi TM, Tumilaar SG, Fatimawali, Idroes R, et al. In silico evaluation of iranian medicinal plant phytoconstituents as inhibitors against main protease and the receptor-binding domain of sars-cov-2. Molecules. 2021;26. doi: 10.3390/molecules26185724 34577194 PMC8470205

[81] Tallei TE, Tumilaar SG, Niode NJ, Fatimawali, Kepel BJ, Idroes R, et al. Potential of Plant Bioactive Compounds as SARS-CoV-2 Main Protease (Mpro) and Spike (S) Glycoprotein Inhibitors: A Molecular Docking Study. Scientifica (Cairo). 2020;2020. doi: 10.1155/2020/6307457 33425427 PMC7773461

