# Supplementary material for: Plant-derived compounds effectively inhibit the main protease of SARS-CoV-2: An in silico approach
Source: PLoS One. 2022 Aug 23;17(8):e0273341. doi: 10.1371/journal.pone.0273341 (PMC9398018; doi:10.1371/journal.pone.0273341)
Supplement: S1 Table — (DOCX) [file pone.0273341.s001.docx]

S1 Table: Chemical name and pubchem CID of different phytochemicals retrived from different plants.

| **SERIAL NO.** | **PLANT NAME** | **CHEMICAL NAME** | **PUBCHEM CID** |
| --- | --- | --- | --- |
| 01 | *Hibiscus rosa-sinensis* Linn. | Methyl sterculate ^1^ | 115261 |
|  |  | Malvalate^1^ | 283632 |
|  |  | Beta-sitosterol^1^ | 222284 |
|  |  | Cyanidin 3-sophoroside^1^ | 44256720 |
|  |  | Quercetin-3-diglucoside^1^ | 10211337 |
|  |  | Quercetin 3,7-diglucoside^1^ | 10121947 |
|  |  | Taraxeryl acetate^1^ | 94225 |
|  |  | Isodecyl octyl ester^1^ | 14902 |
|  |  | Cyanidin- 3,5 -diglucoside^2^ | 44256812 |
|  |  | n triacontane^3^ | 12535 |
|  |  | n – hentriacontane^3^ | 12410 |
|  |  | Neochlorogenic acid^4^ | 5280633 |
|  |  | p-hydroxybenzoic acid^4^ | 135 |
|  |  | Gallic acid^4^ | 370 |
|  |  | Apigenin^4^ | 5280443 |
| 02 | *Homalomena occulta* (Lour.) Schott | Linalool^5^ | 6549 |
|  |  | 4-terpineol^5^ | 11230 |
|  |  | α-terpineol^5^ | 442501 |
|  |  | Geraniol^5^ | 637566 |
|  |  | 4isopropyl-1-methyl-3-  cyclohexen-1-ol^5^ | 11468 |
|  |  | Spathulenol^5^ | 92231 |
|  |  | Epi-α-cadinol^5^ | 12302222 |
|  |  | δ-cadinene^5^ | 12306048 |
|  |  | 1-cyclohexanone^5^ | 11608142 |
|  |  | 2-methyl-2-(3-methyl-2-oxobutyl)^5^ | 592628 |
|  |  | linoleic acid^5^ | 5280450 |
|  |  | 4-(2,6,6-trimethyl-cyclohex-1-enyl)^5^ | 606866 |
|  |  | α-pinen^6^ | 119853 |
|  |  | α-Bisabolol^6^ | 1549992 |
|  |  | Benzyl benzoate^6^ | 2345 |
|  |  | Bicyclogermacren^6^ | 101417559 |
|  |  | (E)-nerolidol^6^ | 5284507 |
|  |  | Oplodiol^7^ | 12313756 |
| 03 | *Houttuynia cordata* Thunb. | Methyl n- ketone nonyl^8^ | 8163 |
|  |  | β-myrcene^8^ | 31253 |
|  |  | Houttuynin^8^ | 122640 |
|  |  | Decanal^8^ | 8175 |
|  |  | Trans-caryophyllene^8^ | 5281515 |
|  |  | Decanoic acid^8^ | 2969 |
|  |  | Camphene^8^ | 6616 |
|  |  | β-pinene^8^ | 440967 |
|  |  | Lauraldehyde^8^ | 8194 |
|  |  | Bornyl acetate^8^ | 6448 |
|  |  | α-pinene^8^ | 440967 |
|  |  | Limonene^8^ | 22311 |
|  |  | 4-terpineol^8^ | 11230 |
|  |  | Caryophyllene oxide^8^ | 1742210 |
|  |  | Nonanol^8^ | 8914 |
|  |  | Linalool^8^ | 6549 |
|  |  | Chlorogenic acid methyl ester^8^ | 6476139 |
|  |  | 2-(4-hydroxyphenyl)ethyl-β-D-glucopyranoside^8^ | 159278 |
|  |  | Catechin^8^ | 9064 |
|  |  | Procyanidin B^8^ | 130556 |
|  |  | Houttuynamide A^8^ | 44521377 |
|  |  | Houttuynoside A^8^ | 4452132 |
|  |  | Aristolactam A^8^ | 148657 |
|  |  | Piperolactam A^8^ | 3081016 |
|  |  | Chlorogenic acid^8^ | 1794427 |
|  |  | Stigmast-4-en-3-one^8^ | 5484202 |
|  |  | 5α-stigmastane-3,6-dione^8^ | 92043363 |
|  |  | Stigmast-4-ene-3,6- dione^8^ | 11743229 |
|  |  | Stigmast-3,6- dione^8^ | 146158184 |
|  |  | Sitoindoside I^8^ | 9832350 |
|  |  | Daucosterol^8^ | 5742590 |
|  |  | β-Sitosterol^8^ | 222284 |
|  |  | β-sitosterol glucoside^8^ | 70697783 |
|  |  | Brassicasterol^8^ | 5281327 |
|  |  | Stigmasterol^8^ | 5280794 |
|  |  | Spinasterol^8^ | 5281331 |
|  |  | Stigmast-4-ene-3,6-dione^8^ | 5490007 |
|  |  | Norcepharadione B^9^ | 189168 |
|  |  | Quercitrin ^9^ | 5280459 |
|  |  | Rutin^10^ | 5280805 |
|  |  | Hyperoside^10^ | 5281643 |
|  |  | Quercetin ^10^ | 5280343 |
|  |  | 2-undecanone ^11^ | 8163 |
|  |  | (Z)-β-ocimene ^11^ | 5320250 |
| 04 | *Imperata cylindrica* (L.) Raeusch*.* | Rutin^12^ | 5280805 |
|  |  | Gallic acid^12^ | 370 |
|  |  | Ellagic acid^12^ | 5281855 |
|  |  | Vanillic acid^13^ | 8468 |
|  |  | Ferulic acid^13^ | 445858 |
|  |  | p-coumaric acid^13^ | 637542 |
|  |  | p-hydroxybenzoic acid^13^ | 135 |
|  |  | Caffeic acid^13^ | 689043 |
|  |  | 2-Methoxyestrone^14^ | 440624 |
|  |  | 11, 16-Dihydroxypregn-4-ene-3, 20-dione^14^ | 22295176 |
|  |  | Tricin^14^ | 5281702 |
|  |  | 5-methoxyflavone^15^ | 94525 |
|  |  | Methyl caffeate^15^ | 689075 |
|  |  | Abscisic acid^15^ | 5280896 |
| 05 | *Inula cappa* (Buch.-Ham. ex D. Don) DC | Isoalantolactone^16^ | 73285 |
|  |  | Germacranolide^16^ | 101616641 |
|  |  | Lupeol^16^ | 259846 |
|  |  | Oleanolic acid^16^ | 10494 |
|  |  | β-sitosterol^16^ | 222284 |
|  |  | Thymol ^16^ | 6989 |
|  |  | Isobutyrate^16^ | 6590 |
|  |  | Capric acid^16^ | 2969 |
|  |  | Caprylicacid^16^ | 379 |
|  |  | Myristic acid^16^ | 11005 |
|  |  | Luteolin^16^ | 5280445 |
|  |  | Apigenin^16^ | 5280443 |
|  |  | Chrysoeriol^16^ | 5280666 |
|  |  | Artemetin^16^ | 5320351 |
|  |  | Luteolin-3-methyl^16^ | 44258196 |
|  |  | Friedelin^16^ | 91472 |
|  |  | Scopoletin^16^ | 5280460 |
|  |  | Isoscopoletin^16^ | 69894 |
|  |  | Scopolin^17^ | 439514 |
|  |  | β-Caryophyllene^18^ | 1742210 |
|  |  | β-bisabolene^18^ | 10104370 |
|  |  | (E)-β-farnesene^18^ | 5281516 |
|  |  | Aurantiamide acetate^18^ | 124319 |
|  |  | Aurantiamide benzoate^18^ | 102153907 |
|  |  | Physcion^18^ | 10639 |
|  |  | Vanillin^18^ | 1183 |
|  |  | Coniferyl aldehyde^18^ | 5280536 |
|  |  | Syringaldehyde^18^ | 8655 |
|  |  | Syringic acid^18^ | 10742 |
|  |  | Azelaic acid^18^ | 2266 |
| 06 | *Itoa orientalis* Hemsl. | 13-deoxyitol A^19^ | 76334115 |
|  |  | Itol A^19^ | 24885741 |
|  |  | Itol B^19^ | 24885738 |
|  |  | Itol C^20^ | 24885970 |
|  |  | Itoside P^21^ | 24879104 |
|  |  | Itoside J^22^ | 24879103 |
|  |  | Itoside K^22^ | 101865243 |
|  |  | Itoside L^22^ | 24879104 |
|  |  | Itoside M^22^ | 101865244 |
|  |  | Itoside N^22^ | 24879215 |
|  |  | Echitin^22^ | 101865246 |
|  |  | 4-hydroxytremulacin^22^ | 16086547 |
|  |  | Poliothyrsoside^22^ | 3084295 |
|  |  | Poliothyrsin^22^ | 44577174 |
|  |  | Homaloside D^22^ | 23928057 |
|  |  | Tremulacin^22^ | 442544 |
|  |  | Itoside A^23^ | 38361549 |
|  |  | Itoside B^23^ | 44577044 |
|  |  | Itoside C^23^ | 44577068 |
|  |  | Itoside D^23^ | 44577124 |
|  |  | Itoside E^23^ | 44577125 |
|  |  | Itoside F^23^ | 44577126 |
|  |  | Itoside G^23^ | 25022462 |
|  |  | Itoside H^23^ | 44577154 |
|  |  | Itoside I^23^ | 101456838 |
|  |  | (¼2-(hydroxymethyl)benzene-1,4-diol)^23^ | 188287 |
|  |  | (¼benzene-1,2-diol)^23^ | 476858 |
| 07 | *Ixeris polycephala* Cass. | KMCP^24^ | 102371598 |
| 08 | *Jasminum nudiflor*  *um* Lindl. | Jasmoside^25^ | 74193152 |
|  |  | Jasnudifloside H^25^ | 102510585 |
|  |  | Jasminin^25^ | 93473214 |
|  |  | Verbascoside^25^ | 5281800 |
|  |  | Syringin^25^ | 5316860 |
|  |  | Jasnudifloside F^26^ | 102510583 |
|  |  | Jasnudifloside G^26^ | 102510584 |
|  |  | Jasnudifloside I^26^ | 102510586 |
|  |  | Jasnudifloside J^26^ | 102510587 |
|  |  | Jasnudifloside L^26^ | 102510589 |
|  |  | Nudifloside D^26^ | 102510590 |
|  |  | Isooleoacteoside^26^ | 11051372 |
|  |  | Jasnudifloside D^26^ | 10677378 |
|  |  | Jasnudifloside E^26^ | 11804000 |
|  |  | Nudifloside A^26^ | 10581755 |
|  |  | Nudifloside B^26^ | 11803920 |
|  |  | Nudifloside C^26^ | 10674051 |
|  |  | Oleoside 11 methyl ester^27^ | 24121278 |
|  |  | Acteoside^27^ | 5281800 |
|  |  | Poliumoside^27^ | 6442411 |
| 09 | *Kadsura heteroclita* (Roxb.) Craib | Lanosta-4^28^ | 46882800 |
|  |  | 24, Triene-3^28^ | 100986972 |
|  |  | 26-Dioic acid^28^ | 6474316 |
|  |  | Changnanic acid^28^ | 137347563 |
|  |  | Heteroclitalactone A^28^ | 101415306 |
|  |  | Heteroclitalactone D^28^ | 101415307 |
|  |  | Schisanlactone B^28^ | 72188601 |
|  |  | Nigranoic acid^28^ | 10814237 |
|  |  | Kadsuranic acid A^28^ | 52914006 |
|  |  | Kadheterilactone A^28^ | 71591176 |
|  |  | Kadheterilactone B^28^ | 71591177 |
|  |  | Kadsurarin^29^ | 101072554 |
|  |  | Kadsulignan C^29^ | 138112788 |
|  |  | Kadsulignan E^29^ | 44445503 |
|  |  | Isolariciresinol^29^ | 160521 |
|  |  | Evofolin B^29^ | 5317306 |
|  |  | α Eudesmol^30^ | 12304196 |
|  |  | 4-Terpineol^30^ | 11230 |
|  |  | δ-Cadinene^30^  δ-Cadinol^30^ | 6432404  91753503 |
|  |  | δ-4-Carene^30^ | 16211586 |
|  |  | Calarene^30^ | 28481 |
|  |  | α-Pinene^30^ | 6654 |
|  |  | 1,8-Cineol^30^ | 2758 |
|  |  | Linalool^30^ | 6549 |
|  |  | α-Cubebene^30^ | 86609 |
|  |  | Caryophyllene^30^ | 5281515 |
| 10 | *Lagerstroemia indica* Linn. | Stroside A^31^ | 132515140 |
|  |  | Stroside B^31^ | 132515141 |
|  |  | Stroside C^31^ | 132515142 |
|  |  | Pterospermin A^31^ | 53364233 |
|  |  | (2R,3S)-dihydrodehydroconiferyl alcohol^31^ | 5274623 |
|  |  | Hovetrichoside A^31^ | 21668725 |
|  |  | Hovetrichoside B^31^ | 21668726 |
|  |  | Ficusol^31^ | 100955863 |
|  |  | Evofolin-B^31^ | 5317306 |
|  |  | Marphenol C^31^ | 71577010 |
|  |  | Quadranoside^31^ | 10327092 |
|  |  | Betulinic acid^31^ | 64971 |
|  |  | Hederagenin^31^ | 73299 |
|  |  | Arjunolic acid^31^ | 73641 |
|  |  | Oleanolic acid^31^ | 10494 |
|  |  | Maslinic acid^31^ | 73659 |
|  |  | Biphenyl^31^ | 7095 |
|  |  | Decamine^31^ | 10649 |
|  |  | Decinine^31^ | 5316415 |
|  |  | Decodine^31^ | 5316425 |
|  |  | Dihydroverticillatine^31^ | 531647 |
|  |  | Lagerstroemine^31^ | 78385190 |
|  |  | Lagerine^31^ | 101473393 |
|  |  | Dihydrolyfoline^31^ | 42640297 |
|  |  | Rutin^31^ | 5280805 |
|  |  | Quercetrin^31^ | 5280459 |
|  |  | Rosemarinic^31^ | 5315615 |
|  |  | Quercetin^31^ | 5280343 |
|  |  | Naringenin^31^ | 932 |
|  |  | Hespertin^31^ | 76315743 |
|  |  | Kampferol^31^ | 5280863 |
|  |  | Rhamnetin^31^ | 5281691 |
|  |  | Apigenin^31^ | 5280443 |
|  |  | Acacetin^31^ | 5280442 |
|  |  | Pyrogallol^31^ | 1057 |
|  |  | Gallic acid^31^ | 370 |
|  |  | 4-aminobenzoic acid^31^ | 978 |
|  |  | Catechin^31^ | 9064 |
|  |  | Catechol^31^ | 289 |
|  |  | Epicatechin^31^ | 72276 |
|  |  | p -hydroxy benzoic acid^31^ | 53648305 |
|  |  | Chlorogenic acid^31^ | 1794427 |
|  |  | Vanillic acid^31^ | 8468 |
|  |  | Caffeic acid31 | 689043 |
|  |  | p-Coumaric acid^31^ | 1549106 |
|  |  | Ferulic acid^31^ | 445858 |
|  |  | α-Coumaric acid^31^ | 9840292 |
|  |  | Benzoic acid^31^ | 243 |
|  |  | Ellagic acid^31^ | 5281855 |
|  |  | Cinnamic acid^31^ | 444539 |
|  |  | Salycilic acid^31^ | 23361 |
|  |  | 3-O-methylgallate^31^ | 54707974 |
|  |  | Tellimagrandin^31^ | 442690 |
|  |  | Isovitexin^31^ | 162350 |
|  |  | Vitexin^31^ | 5280441 |
|  |  | Iso-orientin^31^ | 144221060 |
|  |  | Orientin^31^ | 5281675 |
|  |  | 3- methoxyellagic acid^31^ | 125749 |
|  |  | Luteolin^31^ | 5280445 |
|  |  | 𝛾-sitosterol^31^ | 222284 |
|  |  | (Z)-9-octadecenamide (oleamide)^31^ | 5283387 |
|  |  | Phytol^31^ | 5280435 |
|  |  | 𝛼-tocopherol^31^ | 86472 |
|  |  | Squalene^31^ | 638072 |
|  |  | n-hexadecanoic acid^31^ | 985 |
|  |  | Linolenic acid^31^ | 5280934 |
|  |  | 5-hydroxy methyl furfural^31^ | 237332 |
|  |  | Acetate^31^ | 175 |
|  |  | Campesterol^31^ | 173183 |
|  |  | Ethyl 𝛼-d-glucopyranoside^31^ | 11127487 |
|  |  | 3,7,11,15 tetramethyl-2-hexadecen-1-ol^31^ | 5366244 |
|  |  | Linoleic acid^31^ | 5280450 |
|  |  | 24-methylenecycloartanol^31^ | 94204 |
|  |  | Cis-11-eicosenamide^31^ | 5365374 |
|  |  | Stigmast-5-en-3-ol^31^ | 22012 |
|  |  | Oleate^31^ | 445639 |
|  |  | 𝛾-tocopherol^31^ | 92729 |
|  |  | Hexadecanamide^31^ | 69421 |
|  |  | Octadecanamide^31^ | 31292 |
|  |  | Octadecanoic acid^31^ | 5281 |
|  |  | Stigmasterol^31^ | 5280794 |
|  |  | Glycerol ß-palmitate^31^ | 14900 |
|  |  | Hexadecanoic acid ethyl ester^31^ | 12366 |
|  |  | Pentacosane^31^ | 12406 |
|  |  | Galacturonic acid^31^ | 439215 |
| 11 | *Laggera crispata* (Vahl) Hepper & J.R.I.Wood | Alpha-Pinene^32^ | 6654 |
|  |  | Camphene^32^ | 6616 |
|  |  | Beta-Pinene^32^ | 14896 |
|  |  | Myrcene^32^ | 31253 |
|  |  | Alpha-Phellandrene^32^ | 7460 |
|  |  | Alpha-Terpinene^32^ | 7462 |
|  |  | p-Cymene^32^ | 7463 |
|  |  | Limonene^32^ | 22311 |
|  |  | p-Mentha-3,8-diene^32^ | 521851 |
|  |  | Terpinolene^32^ | 11463 |
|  |  | Linalool^32^ | 6549 |
|  |  | Trans-Sabinene hydrate^32^ | 12315151 |
|  |  | Cis-p-Menth-2-en-1-ol^32^ | 122484 |
|  |  | Trans-p-Menth-2-en-1-ol^32^ | 122484 |
|  |  | Trans-Pinocamphone^32^ | 11038 |
|  |  | Terpinen-4-ol^32^ | 11230 |
|  |  | Alpha-Terpineol^32^ | 17100 |
|  |  | Trans-p-Mentha-1(7),8-dien-2-ol^32^ | 6428442 |
|  |  | Thymol methyl ether^32^ | 14104 |
|  |  | Carvacrol methyl ether^32^ | 80790 |
|  |  | Thymol^32^ | 6989 |
|  |  | Beta-Elemene^32^ | 6918391 |
|  |  | (E)-Caryophyllene^32^ | 5281515 |
|  |  | 2,5-Dimethoxy-p-cymene^32^ | 6427071 |
|  |  | Beta-Humulene^32^ | 21159064 |
|  |  | Alpha-Humulene^32^ | 6508206 |
|  |  | (E)-beta-Farnesene^32^ | 5281517 |
|  |  | Gamma-Muurolen^32^ | 12313020 |
|  |  | Phenyl ethyl 2-methylbutanoate^32^ | 22162426 |
|  |  | Delta-Selinene^32^ | 520383 |
|  |  | 4-epi-cis-Dihydroagarofuran^32^ | 521889 |
|  |  | Germacrene A^32^ | 5835162 |
|  |  | Gamma-Cadinene^32^ | 92313 |
|  |  | Cubebol^32^ | 11276107 |
|  |  | Alpha-Cadinene^32^ | 12306048 |
|  |  | Alpha-Calacorene^32^ | 12302243 |
|  |  | Elemol^32^ | 92138 |
|  |  | Germacrene D-4-ol^32^ | 5352847 |
|  |  | Caryophyllene oxide^32^ | 1742210 |
|  |  | Guaiol^32^ | 227829 |
|  |  | Humulene epoxide II^32^ | 10704181 |
|  |  | 10-epi-gamma-Eudesmol^32^ | 6430754 |
|  |  | Epi-alpha-Cadinol^32^ | 160799 |
|  |  | Epi-alpha-Muurolol^32^ | 3084331 |
|  |  | Alpha-Muurolol^32^ | 100949538 |
|  |  | Beta-Eudesmol^32^ | 91457 |
|  |  | 7-epi-alpha-Eudesmol^32^ | 12304196 |
|  |  | Bulnesol^32^ | 90785 |
|  |  | Beta-Bisabolol^32^ | 27208 |
|  |  | Eudesma-4(15),7-dien-1b-ol^32^ | 6429131 |
|  |  | Eudesm-7(11)-en-4-ol^32^ | 6432454 |
|  |  | (Z,E)-Farnesol^32^ | 1549108 |
|  |  | (E,E)-Farnesol^32^ | 445070 |
|  |  | Eudesmol acetate^32^ | 146020857 |
|  |  | Alpha-thujene^32^ | 17868 |
|  |  | (E)-beta-Ocimene^32^ | 5281553 |
|  |  | Gamma-Terpinene^32^ | 7461 |
| 12 | *Leonurus japonicus* Houtt. | Ajugoside^33^ | 9865184 |
|  |  | (−)-Loliolide^33^ | 100332 |
|  |  | Staphylionoside E^33^ | 11211418 |
|  |  | Citroside A^33^ | 14312562 |
|  |  | Megastigmane^33^ | 637237 |
|  |  | 3-Oxo-α-ionone^33^ | 387132169 |
|  |  | (+)-Dehydrovomifoliol^33^ | 688492 |
|  |  | (6S,9R)-Vomifoliol^33^ | 5280462 |
|  |  | (3S,5R,6S,7E,9R)-5,6-Epoxy-3,9-dihydroxy-7-megastigmene^33^ | 23251169 |
|  |  | 5,6-Epoxy-3-hydroxy-7-megastigmen-9-one^33^ | 14605580 |
|  |  | (+)-3-Hydroxy-β-ionone^33^ | 5363700 |
|  |  | Chamigrenal^33^ | 177096 |
|  |  | β-Chamigrenic acid^33^ | 13648247 |
|  |  | Arteannuin B^33^ | 6543478 |
|  |  | Saniculamoid D^33^ | 53360479 |
|  |  | Prehispanolone^33^ | 196854 |
|  |  | Preleoheterin^33^ | 15726752 |
|  |  | 13-Epi-preleoheterin^33^ | 102187096 |
|  |  | Leoheteronone B^33^ | 101774198 |
|  |  | 15-Epileoheteronone B^33^ | 101774199 |
|  |  | Leopersin B^33^ | 101685533 |
|  |  | 15-Epileopersin B^33^ | 101685704 |
|  |  | Leoheteronone D^33^ | 101774200 |
|  |  | 15-Epileoheteronone D^33^ | 101774201 |
|  |  | Leoheteronone E^33^ | 101774202 |
|  |  | 15-Epileoheteronone E^33^ | 101774203 |
|  |  | Leoheteronone C^33^ | 11603911 |
|  |  | Leosibirinone A^33^ | 102054462 |
|  |  | Leosibirone B^33^ | 52951193 |
|  |  | 15-Epi-leosibirone B^33^ | 52951194 |
|  |  | Leopersin C^33^ | 10521953 |
|  |  | 15-Epileopersin C^33^ | 15548655 |
|  |  | Leopersin G^33^ | 10854201 |
|  |  | Leoheteronin A^33^ | 11232340 |
|  |  | Leoheteronin C^33^ | 11438980 |
|  |  | Leoheteronin B^33^ | 11370504 |
|  |  | 15-Methoxyleoheteronin B^33^ | 101891061 |
|  |  | Leoheterin^33^ | 15726751 |
|  |  | Hispanolone^33^ | 5458747 |
|  |  | Galeopsin^33^ | 92966417 |
|  |  | Hispanone^33^ | 10402561 |
|  |  | Leojaponin^33^ | 12187536 |
|  |  | 8,9-Secohispanolone^33^ | 70904801 |
|  |  | Villenol^33^ | 21582485 |
|  |  | Leoheteronin D^33^ | 1747800 |
|  |  | Leonuketal^33^ | 122372769 |
|  |  | Isoleojaponin^33^ | 122224470 |
|  |  | Leonurusoleanolide A^33^ | 160697341 |
|  |  | Leonurusoleanolide B^33^ | 275489827 |
|  |  | Leonurusoleanolide C^33^ | 160707980 |
|  |  | Leonurusoleanolide D^33^ | 275489829 |
|  |  | Leonurusoleanolide J^33^ | 102362411 |
|  |  | Leonurusoleanolide I^33^ | 102362410 |
|  |  | Oleanolic acid^33^ | 10494 |
|  |  | Urjinolic acid^33^ | 146156259 |
|  |  | β-Amyrenol^33^ | 225689 |
|  |  | α-Amyrin^33^ | 73170 |
|  |  | Zizyberenalic acid^33^ | 15958448 |
|  |  | Lupeol^33^ | 259846 |
|  |  | Betulin^33^ | 72326 |
|  |  | Betulinic acid^33^ | 64971 |
|  |  | Dihydrobetulin^33^ | 10527286 |
|  |  | Cornusalterin D^33^ | 50994224 |
|  |  | Cornusalterin J^33^ | 50994309 |
|  |  | β-Sitosterol^33^ | 222284 |
|  |  | (24S)-Saringosterol^33^ | 14161396 |
|  |  | β-Sitosterone^33^ | 9801811 |
|  |  | Demethylincisterol A3^33^ | 10969647 |
|  |  | Cyathisterone^33^ | 70698238 |
|  |  | Cycloleonuripeptide A^33^ | 102062685 |
|  |  | Cycloleonuripeptide B^33^ | 102062686 |
|  |  | Cycloleonuripeptide D^33^ | 101887656 |
|  |  | Cycloleonuripeptide E^33^ | 103636027 |
|  |  | Cycloleonuripeptide F^33^ | 11651008 |
|  |  | Cycloleonurinin^33^ | 195797 |
|  |  | Yibeinoside A^33^ | 146673054 |
|  |  | Juzirine^33^ | 3085285 |
|  |  | Leonurine^33^ | 161464 |
|  |  | Stachydrine^33^ | 115244 |
|  |  | Choline^33^ | 305 |
|  |  | Trigonelline^33^ | 5570 |
|  |  | 3-Hydroxypyridine^33^ | 7971 |
|  |  | 3-Hydroxy-2-methylpyridine^33^ | 70719 |
|  |  | 5-Hydroxy-2-hydroxymethylpyridine^33^ | 419490 |
|  |  | Uracil^33^ | 1174 |
|  |  | 5-Methyluracil^33^ | 1135 |
|  |  | Guanosine^33^ | 135398635 |
|  |  | L-tryptophan^33^ | 6305 |
|  |  | L-Phenylalanine^33^ | 6140 |
|  |  | Valine^33^ | 6287 |
|  |  | N-Isobutyl-L-valine^33^ | 14584556 |
|  |  | L-Pyroglutamate acid methyl ester^33^ | 10868485 |
|  |  | Alanine^33^ | 5950 |
|  |  | Genkwanin^33^ | 5281617 |
|  |  | Hydroxygenkwanin^33^ | 5318214 |
|  |  | Luteolin^33^ | 5280445 |
|  |  | Apigenin^33^ | 5280443 |
|  |  | Wogonin^33^ | 5281703 |
|  |  | 5,7,3′,4′,5′-Pentamethoxyflavone^33^ | 44259720 |
|  |  | Cosmosiin^33^ | 5280704 |
|  |  | Spinosin^33^ | 155692 |
|  |  | Linarin^33^ | 5317025 |
|  |  | Kaempferol^33^ | 5280863 |
|  |  | Quercetin^33^ | 5280343 |
|  |  | Myricetin^33^ | 5281672 |
|  |  | Isoquercitrin^33^ | 5280804 |
|  |  | Hyperoside^33^ | 5281643 |
|  |  | Nicotiflorin^33^ | 5318767 |
|  |  | Kaempferol-3-neohesperidoside^33^ | 5318761 |
|  |  | Rutin^33^ | 5280805 |
|  |  | Tiliroside^33^ | 5320686 |
|  |  | Leonurusoide A^33^ | 102219745 |
|  |  | Leonurusoide C^33^ | 102219747 |
|  |  | Leonurusoide E^33^ | 102219749 |
|  |  | Heteronoside^33^ | 91885068 |
|  |  | Leonurusoide B^33^ | 102219746 |
|  |  | Leonurusoide D^33^ | 102219748 |
|  |  | 2′′′-Syringylrutin^33^ | 102054463 |
|  |  | Daidzein^33^ | 5281708 |
|  |  | Ferulic acid^33^ | 445858 |
|  |  | Martynoside^33^ | 5319292 |
|  |  | Bergapten^33^ | 2355 |
|  |  | Xanthotoxin^33^ | 4114 |
|  |  | Isopimpinellin^33^ | 68079 |
|  |  | Imperatorin^33^ | 10212 |
|  |  | Nodakenin^33^ | 73191 |
|  |  | Murrayone^33^ | 5319964 |
|  |  | Auraptenol^33^ | 13343541 |
|  |  | Osthol^33^ | 10228 |
|  |  | Meransin hydrate^33^ | 129819816 |
|  |  | Dimethylgomisin J^33^ | 43595 |
|  |  | GomisinK1^33^ | 78385003 |
|  |  | Sesamin^33^ | 72307 |
|  |  | (−)-Syringaresinol^33^ | 332426 |
|  |  | Leonuriside B^33^ | 100929502 |
|  |  | Leonoside E^33^ | 57325810 |
|  |  | Cistanoside E^33^ | 21632979 |
|  |  | Leonoside F^33^ | 57325811 |
|  |  | Leonuriside A^33^ | 14237625 |
|  |  | 4-Hydroxybenzaldehyde^33^ | 126 |
|  |  | Vanillin^33^ | 1183 |
|  |  | Syringic acid^33^ | 10742 |
|  |  | Syringic acid methyl ester^33^ | 70164 |
|  |  | Gallic acid^33^ | 370 |
|  |  | 3,4,5-Trimethoxybenzoic acid^33^ | 8357 |
|  |  | 1-(3-Ethylphenyl)ethane-1,2-diol^33^ | 129686768 |
|  |  | 2,6-Dimethyl-2E,7-octadiene-1,6-diol^33^ | 5280678 |
|  |  | Arachidic acid^33^ | 10467 |
|  |  | Heneicosanoic acid^33^ | 16898 |
|  |  | Heptacosanoic acid^33^ | 23524 |
|  |  | Methyl myristate^33^ | 31284 |
|  |  | 1,5,8-p-Menthatriene^34^ | 527424 |
|  |  | Menthone^34^ | 26447 |
|  |  | p-Menth-4-en-3-one^34^ | 107372 |
|  |  | Menthol^34^ | 1254 |
|  |  | 4-Terpineol^34^ | 11230 |
|  |  | β-Terpineol^34^ | 8748 |
|  |  | Perillyl acetate^34^ | 61780 |
|  |  | Camphor^34^ | 2537 |
|  |  | Borneol^34^ | 64685 |
|  |  | Bornyl acetate^34^ | 6448 |
|  |  | Camphene^34^ | 6616 |
|  |  | α-Pinene^34^ | 6654 |
|  |  | α-Pyronene^34^ | 10581 |
|  |  | α-Fenchene^34^ | 28930 |
|  |  | Elsholtzione^34^ | 521240 |
|  |  | β-Caryophyllene^34^ | 5281515 |
|  |  | Caryophyllene oxide^34^ | 1742210 |
|  |  | Isocaryophyllene^34^ | 5281522 |
|  |  | Aromadendrane^34^ | 520381 |
|  |  | Dehydroaromadendrane^34^ | 21595261 |
|  |  | Aromadendrene^34^ | 91354 |
|  |  | Palustrol^34^ | 110745 |
|  |  | Spathulenol^34^ | 92231 |
|  |  | α-Muurolene^34^ | 12306047 |
|  |  | γ-Cadinene ^34^ | 92313 |
|  |  | δ-Cadinene^34^ | 441005 |
|  |  | Germacrene D^34^ | 5317570 |
|  |  | α-Copaene^34^ | 19725 |
|  |  | β-Bourbonene^34^ | 62566 |
|  |  | β-Patchoulene^34^ | 101731 |
|  |  | Patchouli alcohol^34^ | 10955174 |
|  |  | β-Cubebene^34^ | 93081 |
|  |  | β-Elemene^34^ | 6918391 |
|  |  | Irisone^34^ | 5282108 |
|  |  | Phytol^34^ | 5280435 |
|  |  | Phytone^34^ | 10408 |
|  |  | Isopimara-8,15-diene^34^ | 10084655 |
|  |  | Dehydroabietane^34^ | 6432211 |
|  |  | n-Dodecane^34^ | 8182 |
|  |  | n-Tridecane^34^ | 12388 |
|  |  | 2-Methyldecane^34^ | 23415 |
|  |  | n-Tetradecane^34^ | 12389 |
|  |  | n-Pentadecane^34^ | 12391 |
|  |  | n-Hexadecane^34^ | 11006 |
|  |  | n-Heptadecane^34^ | 12398 |
|  |  | n-Octadecane^34^ | 11635 |
|  |  | n-Nonadecane^34^ | 12401 |
|  |  | n-Docosane^34^ | 12405 |
|  |  | Artemisia triene^34^ | 5320377 |
|  |  | 1-Dodecene^34^ | 8183 |
|  |  | Tetradecenal^34^ | 116625 |
|  |  | (Z)-7-Hexadecenal^34^ | 5364438 |
|  |  | Methyl palmitate^34^ | 8181 |
|  |  | Methyl octadecenoate^34^ | 5370350 |
|  |  | Methyl linoleate^34^ | 5284421 |
|  |  | Methyl linolelaidate^34^ | 5362793 |
|  |  | 1,2,3,4,5,8-Hexahydronaphthalene^34^ | 142062 |
|  |  | Benzyl benzoate^34^ | 2345 |
|  |  | Diisobutyl phthalate^34^ | 6782 |
|  |  | Quercetin-3-neohesperidoside^35^ | 5491657 |
|  |  | Staphylionoside E^35^ | 11211418 |
|  |  | Lavandulifolioside^35^ | 14034195 |
|  |  | (+)-Dehydrovomifoliol^35^ | 688492 |
|  |  | Cichoric acid^35^ | 5281764 |
| 13 | *Lespedeza cuneata* (Dum. Cours.) G. Don | Cuneataside E^36^ | 11706085 |
|  |  | Beta-sitosterol^37^ | 222284 |
|  |  | Beta-sitosterol glucoside^37^ | 5742590 |
|  |  | Quercetin^37^ | 5280343 |
|  |  | Kaempferol^37^ | 5280863 |
|  |  | Isovitexin^37^ | 162350 |
|  |  | Hirsutrin^37^ | 5280804 |
|  |  | Nicotiflorin^37^ | 5318767 |
|  |  | Vitexin^37^ | 5280441 |
|  |  | Astragalin^37^ | 5282102 |
|  |  | Trifolin^37^ | 5282149 |
|  |  | Isorhamnetin^37^ | 5281654 |
|  |  | Benzyl-Beta-D-glucopyranoside^37^ | 13254166 |
|  |  | Homovanillyl alcohol^37^ | 16928 |
|  |  | Loroxanthin^37^ | 16061271 |
|  |  | 7R,8S-dihydrodehydrodiconiferyl alcohol^37^ | 5274623 |
|  |  | Pinitol^37^ | 230881 |
|  |  | Tannins^37^ | 250395 |
|  |  | Isoorientin^37^ | 114776 |
|  |  | Vicenin II^37^ | 442664 |
|  |  | Desmodin^37^ | 13338925 |
|  |  | Avicularin^37^ | 5490064 |
|  |  | Juglanin^37^ | 5318717 |
|  |  | Hyperin^37^ | 5281643 |
| 14 | *Leycesteria formosa* Wall. | cis-Caffeic acid^38^ | 1549111 |
|  |  | trans-Caffeic acid^38^ | 2518 |
|  |  | Luteolin^38^ | 5280445 |
|  |  | Apigenin^38^ | 5280443 |
|  |  | Ursolic acid^38^ | 64945 |
|  |  | Caffeic acid methyl ester^38^ | 689075 |
|  |  | 3’-8” Biapigenin^39^ | 10414856 |
|  |  | Podocarpusflavone A^39^ | 5320644 |
| 15 | *Ligusticum sinense* Oliv. | Z-ligustilide^40^ | 5319022 |
|  |  | Levistolide A^40^ | 70698035 |
|  |  | Senkyunolide B^40^ | 5281559 |
|  |  | 3-butylphthalide^40^ | 61361 |
|  |  | Riligustilide^40^ | 6442656 |
|  |  | Neocnidilide^40^ | 3083857 |
|  |  | Senkyunolide A^40^ | 3085257 |
|  |  | (3S, 3aR)- (–)-Sedanolide^41^ | 3083857 |
|  |  | Senkyunolide N^41^ | 15138552 |
|  |  | Senkyunolide I^41^ | 11521428 |
|  |  | Senkyunolide H^41^ | 13965088 |
|  |  | Sedanonic acid^41^ | 12367058 |
|  |  | Alpha-thujene^42^ | 17868 |
|  |  | Alpha-pinene^42^ | 6654 |
|  |  | Sabinene^42^ | 18818 |
|  |  | Beta-pinene^42^ | 14896 |
|  |  | Myrcene^42^ | 31253 |
|  |  | Alpha-phellandrene^42^ | 7460 |
|  |  | Delta-3-Carene^42^ | 26049 |
|  |  | Alpha-terpinene^42^ | 7462 |
|  |  | P-Cymene^42^ | 7463 |
|  |  | Beta-ocimene^42^ | 18756 |
|  |  | Gamma-terpinene^42^ | 7461 |
|  |  | n-Octanol^42^ | 957 |
|  |  | Alpha-terpinolene^42^ | 11463 |
|  |  | n-Nonanal^42^ | 31289 |
|  |  | Linalool^42^ | 6549 |
|  |  | Heptyl acetate^42^ | 8159 |
|  |  | Myrcenol^42^ | 10975 |
|  |  | n-Butyl angelate^42^ | 5364789 |
|  |  | Camphor^42^ | 2537 |
|  |  | Isoamyl n-valerate^42^ | 74901 |
|  |  | Isoborneol^42^ | 6321405 |
|  |  | Borneol^42^ | 64685 |
|  |  | Terpineol-4^42^ | 11230 |
|  |  | Alpha-terpineol^42^ | 17100 |
|  |  | n-Decanal^42^ | 8175 |
|  |  | Verbenone^42^ | 29025 |
|  |  | Bornyl acetate^42^ | 6448 |
|  |  | Isobornyl acetate^42^ | 247573 |
|  |  | n-Undecanal^42^ | 8186 |
|  |  | Carvacrol^42^ | 10364 |
|  |  | Alpha-copaene^42^ | 19725 |
|  |  | Beta-elemene^42^ | 6918391 |
|  |  | Beta-caryophyllene^42^ | 5281515 |
|  |  | Trans-Beta-Farnesene^42^ | 5281517 |
|  |  | alpha-Humulene^42^ | 5281520 |
|  |  | gamma-Patchoulene^42^ | 521302 |
|  |  | Beta-selinene^42^ | 442393 |
|  |  | gamma-Cadinene^42^ | 92313 |
|  |  | Ligustilide^42^ | 5319022 |
| 16 | *Lonicera maackii* (Rupr.) Maxim. | Secoxyloganin^43^ | 162868 |
|  |  | Loganin^43^ | 87691 |
|  |  | Oleanolic acid^43^ | 10494 |
|  |  | Hederagenin^43^ | 73299 |
|  |  | Erythrodiol^43^ | 101761 |
|  |  | β-sitosterol^43^ | 222284 |
|  |  | β-daucosterol^43^ | 5742590 |
|  |  | Stigmasterol^43^ | 5280794 |
|  |  | Digitoxin^43^ | 441207 |
|  |  | Hexane^43^ | 8058 |
|  |  | 2,4-dimethylpentane^43^ | 7907 |
|  |  | 1-methyl-4-isopropylbenzene^43^ | 7463 |
|  |  | 2,6,10-trimethyltetradecane^43^ | 85785 |
|  |  | Pentadecane^43^ | 12391 |
|  |  | Methyl 12,15-octadecadienoate^43^ | 548866 |
|  |  | 11,14-octadecadienoic acid methyl ester^43^ | 5365677 |
|  |  | 12,15-octadecadienoic acid methyl ester^43^ | 5365571 |
|  |  | Methylene bis[6-tert-butyl] p-cresol^43^ | 8398 |
|  |  | 10-octadecenoic acid^43^ | 12745 |
|  |  | 2-bromo- octadecanoic acid^43^ | 97917 |
|  |  | Phthalic acid mono[2-ethylhexyl] ester^43^ | 20393 |
|  |  | 2-hexenal^43^ | 5281168 |
|  |  | Phenylacetaldehyde^43^ | 998 |
|  |  | Nonanal^43^ | 31289 |
|  |  | Decanal^43^ | 8175 |
|  |  | β-cyclocitral^43^ | 9895 |
|  |  | Trans-2-hexen-1-ol^43^ | 5318042 |
|  |  | Linalool^43^ | 6549 |
|  |  | Phenethyl alcohol^43^ | 6054 |
|  |  | (-)-Terpinen-4-Ol^43^ | 5325830 |
|  |  | 3-Heptene^43^ | 11612 |
|  |  | Dodecane^43^ | 8182 |
|  |  | Tridecane^43^ | 12388 |
|  |  | Hexadecane^43^ | 11006 |
|  |  | (2,6,6-Trimethyl- 2-hydroxycyclohexylidene)Acetic acid lactone^43^ | 27209 |
|  |  | Trans-2-hexenyl-hexanoate^43^ | 5352973 |
|  |  | Damascenone^43^ | 5366074 |
|  |  | Geranylacetone^43^ | 1549778 |
|  |  | 1,1,6-trimethyl- 1,2,3,4-tetrahydro-naphthalene^43^ | 68057 |
|  |  | Tetradecane^43^ | 12389 |
|  |  | 1-ethyl-2,3-dimethylbenzene^43^ | 13621 |
|  |  | (-) Farnesene^43^ | 5281516 |
|  |  | Pentadecane^43^ | 12391 |
|  |  | Hexadecane^43^ | 11006 |
|  |  | Octadecane^43^ | 11635 |
|  |  | Phytane^43^ | 12523 |
|  |  | Nonadecane^43^ | 12401 |
|  |  | Hexadecanoic acid^43^ | 985 |
|  |  | Eicosane^43^ | 8222 |
|  |  | Heneicosane^43^ | 12403 |
|  |  | Docosane^43^ | 12405 |
|  |  | Methyl behenate^43^ | 13584 |
|  |  | Nonacosane^43^ | 12409 |
|  |  | Methyl lignocerate^43^ | 75546 |
|  |  | Ethanedioic acid dimethyl ester^43^ | 11120 |
|  |  | (Z)-3-methyl-4-undecene^43^ | 5364744 |
|  |  | P-xylene^43^ | 7809 |
|  |  | Cyclohexanol^43^ | 7966 |
|  |  | 2,3-dimethyl-3-hexanol^43^ | 107235 |
|  |  | Benzaldehyde^43^ | 240 |
|  |  | 5-ethyl-2-furaldehyde^43^ | 89989 |
|  |  | Benzeneacetaldehyde^43^ | 998 |
|  |  | 1,2,3,4-tetramethyl-benzene^43^ | 10263 |
|  |  | Naphthalene^43^ | 931 |
|  |  | Cantharidin^43^ | 5944 |
|  |  | 3,5-dimethyl-benzoic acid methyl ester^43^ | 32786 |
|  |  | (-)-isolongifolol methyl ether^43^ | 91700604 |
|  |  | (-)-isolongifolol^43^ | 16217350 |
|  |  | Imiprothrin^43^ | 123622 |
|  |  | D-bicuculline^43^ | 10237 |
|  |  | Scoparone^43^ | 8417 |
|  |  | Picrotoxin^43^ | 31304 |
|  |  | 2,4,7,14-tetramethyl-4-vinyl-tricyclo [5.4.3.0(1,8)] tetradecan-6-ol^43^ | 590916 |
|  |  | Dibutyl phthalate^43^ | 3026 |
|  |  | 2,4-bis(1,1-dimethylethyl)-phenol^43^ | 159495 |
|  |  | Benzoic acid-2,5-bis(trimethylsiloxy)-trimethylsilyl ester^43^ | 520779 |
|  |  | Pentacosane^43^ | 12406 |
|  |  | Tetracosane^43^ | 12592 |
|  |  | Dotriacontane^43^ | 11008 |
|  |  | (Z,Z)- 9-hexadecenoic acid-9-octadecenyl ester^43^ | 5364677 |
|  |  | Rutin^43^ | 5280805 |
|  |  | Hyperoside^43^ | 5281643 |
|  |  | Luteolin^43^ | 5280445 |
|  |  | Apigenin^43^ | 5280443 |
|  |  | Apigenin-7-glucoside^43^ | 5280704 |
|  |  | Kaempferol^43^ | 5280863 |
|  |  | Kaempferol-3-glucoside^43^ | 5282102 |
|  |  | Chlorogenic acid^43^ | 1794427 |
|  |  | Isochlorogenic acid A^43^ | 6474310 |
|  |  | Isochlorogenic acid C^43^ | 6474309 |
|  |  | Methyl chlorogenic acid^43^ | 6476139 |
|  |  | Tripalmitin^43^ | 11147 |
|  |  | Erythrodiol^44^ | 101761 |
|  |  | Uvaol^44^ | 92802 |
|  |  | Daucosterol^44^ | 5742590 |
| 17 | *Luculia pinceana* Hook. var. pinceana | Paeonol^45^ | 11092 |
|  |  | (E,E)-α-farnesene^45^ | 5281516 |
|  |  | Cyclosativene^45^ | 519960 |
|  |  | δ-cadinene^45^ | 6432404 |
|  |  | (1S)-2,6,6-Trimethylbicyclo[3.1.1]hept-2-ene^45^ | 6654 |
|  |  | Santolina triene^45^ | 519872 |
|  |  | α-Pinene^45^ | 6654 |
|  |  | Limonene^45^ | 22311 |
|  |  | 3-Carene^45^ | 26049 |
|  |  | (Z)-Verbenol^45^ | 61126 |
|  |  | (E)-β-Ocimene^45^ | 5281553 |
|  |  | α-Campholenal^45^ | 1252759 |
|  |  | α-Santoline alcohol^45^ | 565379 |
|  |  | Perilla alcohol^45^ | 10819 |
|  |  | Nonanoic acid,ethyl ester^45^ | 31251 |
|  |  | Megastigma-4,6(E),8(E)-triene^45^ | 5369483 |
|  |  | Hexyl caprylate^45^ | 14228 |
|  |  | Pentanoic acid, 2,2,4-trimethyl 3-carboxyisopropyl, isobutyl ester^45^ | 551220 |
|  |  | Methyl salicylate^45^ | 4133 |
|  |  | Phenol, 2,6-bis(1,1-dimethylethyl)-4-(1-methylpropyl)^45^ | 86583 |
|  |  | α-Cubebene^45^ | 86609 |
|  |  | Isoledene^45^ | 530426 |
|  |  | Caryophyllene^45^ | 5281515 |
|  |  | β-Ylangene^45^ | 519779 |
|  |  | (-)-β-Cadinene^45^ | 10657 |
|  |  | γ-Muurolene^45^ | 12313020 |
|  |  | Cubebol^45^ | 11276107 |
|  |  | 4- epi-Cubebol^45^ | 12304217 |
|  |  | Cedrol^45^ | 65575 |
|  |  | α-Acorenol^45^ | 11972555 |
|  |  | Cubenol^45^ | 519857 |
| 18 | *Luffa acutangula* (Linn.) Roxb. | Apigenin-7-glucoside^46^ | 5280704 |
|  |  | Luteolin-7-glucoside^46^ | 5280637 |
|  |  | 1,8-dihydroxy-4- methylanthracene-9,10-dione^46^ | 129853343 |
|  |  | Myristic acid^46^ | 11005 |
|  |  | Palmitic acid^46^ | 985 |
|  |  | Stearic acid^46^ | 5281 |
|  |  | Oleic acid^46^ | 445639 |
|  |  | Linoleic acid^46^ | 5280450 |
|  |  | Oleanolic acid^46^ | 10494 |
|  |  | Acutoside-A^46^ | 21606142 |
|  |  | Acutoside-B^46^ | 3536509 |
|  |  | Acutoside-D^46^ | 4353789 |
|  |  | Acutoside-E^46^ | 101596471 |
|  |  | Acutoside-F^46^ | 131751146 |
|  |  | Acutoside-G^46^ | 4555947 |
|  |  | Acutoside-C^46^ | 131753039 |
|  |  | 3-Methyl-1-butanol^46^ | 31260 |
|  |  | 4,5-Dimethyl-1-hexene^46^ | 27683 |
|  |  | α-Thujene^46^ | 17868 |
|  |  | α-Pinene^46^ | 6654 |
|  |  | Sabinene^46^ | 18818 |
|  |  | β-Pinene^46^ | 14896 |
|  |  | β-Myrcene^46^ | 31253 |
|  |  | D,L-Limonene^46^ | 22311 |
|  |  | 1,8-Cineole^46^ | 2758 |
|  |  | β-Ocimene (Z)^46^ | 5320250 |
|  |  | β-Ocimene (E)^46^ | 5281553 |
|  |  | β-Terpinene^46^ | 66841 |
|  |  | γ-Terpinene^46^ | 7461 |
|  |  | trans-Linalool oxide^46^ | 6432254 |
|  |  | trans-Dihydrocarvone^46^ | 6432474 |
|  |  | Linalool^46^ | 6549 |
|  |  | cis-Sabinene hydrate^46^ | 101629835 |
|  |  | α-Thujone^46^ | 261491 |
|  |  | 2-methyl-6-methylene-1,7- octadien-3-one^46^ | 93231 |
|  |  | 3,4-dimethyl-2,4,6-octatriene^46^ | 5371124 |
|  |  | α-Terpineol^46^ | 17100 |
|  |  | 1H-Indole^46^ | 798 |
|  |  | Neryl acetate^46^ | 1549025 |
|  |  | 2,3-dihydro,3,5-dihydroxy-6- methyl-(4H)-pyran-4-one^46^ | 119838 |
|  |  | 3,7,11,15-tetramethyl-2- hexadecen-1-ol^46^ | 5366244 |
|  |  | 9,12,15-octadecatrienoic acid methyl ester^46^ | 5367462 |
|  |  | Citronellyl tiglate^46^ | 6386037 |
|  |  | Ascorbic acid^46^ | 54670067 |
|  |  | Carotene^46^ | 6419725 |
|  |  | Lignin^47^ | 73555271 |
|  |  | Phenol^47^ | 996 |
|  |  | Cucurbitacin B^47^ | 5281316 |
|  |  | Hexadecanoic acid ethyl ester^48^ | 12366 |
|  |  | 9, 12, 15-octadecatrienoic acid^48^ | 5282822 |
|  |  | Hentriacontane^48^ | 12410 |
|  |  | Hexadecanoic acid methyl ester^48^ | 10928805 |
|  |  | n-Hexadecanoic acid^48^ | 985 |
|  |  | Triacontane^48^ | 12535 |
|  |  | Tetratriacontane^48^ | 26519 |
|  |  | 9, 12, 15-octadecatrienoic acid methyl ester^48^ | 9316 |
|  |  | 4H-Pyran-4-one, 2,3-dihydro-3,5-dihydroxy-6-methyl-^49^ | 119838 |
|  |  | 9-Octadecynoic acid^49^ | 68167 |
|  |  | 9,12,15-Octadecatrienoic acid, (Z,Z,Z)-^49^ | 5280934 |
|  |  | 1,2-Benzenedicarboxylic acid, diisooctyl ester^49^ | 33934 |
|  |  | Bicyclo[4.4.0]dec-2-ene-4-ol, 2-methyl-9-(prop-1-en-3- ol-2-yl)-^49^ | 535256 |
|  |  | Diazoprogesterone^49^ | 543575 |
|  |  | 1,6,10,14-Hexadecatetraen-3-ol, 3,7,11,15-tetramethyl-, (E,E)-^49^ | 5365872 |
| 19 | *Lycium chinense* Mill. | Palmitic acid^50^ | 985 |
|  |  | Palmitoleic acid^50^ | 445638 |
|  |  | Stearic acid^50^ | 5281 |
|  |  | Oleic acid^50^ | 445639 |
|  |  | Linoleic acid^50^ | 5280450 |
|  |  | Arachidic acid^50^ | 10467 |
|  |  | α-Linolenic acid^50^ | 5280934 |
|  |  | Gondoic acid^50^ | 5282768 |
|  |  | Behenic acid^50^ | 8215 |
|  |  | Lignoceric acid^50^ | 11197 |
|  |  | L-ascorbic acid^51^ | 54670067 |
|  |  | Provitamin A^51^ | 5280489 |
|  |  | α-amylase^51^ | 62698 |
|  |  | Tetragalloyl glucose^51^ | 73178 |
|  |  | Di-O-caffeoylquinic acid^51^ | 13604687 |
|  |  | Chlorogenic acid^51^ | 1794427 |
|  |  | Quercetin-3-O-glucuronide^51^ | 5274585 |
|  |  | Quercetin-3-O-rutinoside^51^ | 5280805 |
|  |  | Quercetin-3-O-robinobioside^51^ | 10371536 |
|  |  | Isorhamnetin-3-O-rutinoside^51^ | 5481663 |
|  |  | p-Coumaric acid^52^ | 637542 |
|  |  | Ferulic acid^52^ | 445858 |
|  |  | Isoquercitrin^52^ | 5280804 |
|  |  | Rutin^52^ | 5280805 |
|  |  | Quercitrin^52^ | 5280459 |
|  |  | Quercetin^52^ | 5280343 |
|  |  | Kaempferol^52^ | 5280863 |
|  |  | Caftaric acid^52^ | 6440397 |
|  |  | Sinapic acid^52^ | 637775 |
|  |  | Cichoric acid^52^ | 5281764 |
|  |  | Hyperoside^52^ | 5281643 |
|  |  | Myricetin^52^ | 5281672 |
|  |  | Fisetin^52^ | 5281614 |
|  |  | Patuletin^52^ | 5281678 |
|  |  | Luteolin^52^ | 5280445 |
|  |  | Apigenin^52^ | 5280443 |
|  |  | Lyciumoside I^53^ | 10326675 |
|  |  | Lyciumoside II^53^ | 11765826 |
|  |  | Lyciumoside III^53^ | 131752673 |
|  |  | Lyciumoside VI^53^ | 131751439 |
|  |  | Lyciumoside V^53^ | 100928037 |
|  |  | Lyciumoside VI^53^ | 100928038 |
|  |  | Lyciumoside VII^53^ | 348288723 |
|  |  | Lyciumoside VIII^53^ | 131751390 |
|  |  | Arachidic acid^54^ | 10467 |
|  |  | Lyciumamide A^55^ | 122218206 |
| 20 | *Lysimachia christiniae* Hance | Amentoflavone^56^ | 5281600 |
|  |  | Hyperin^56^ | 5281643 |
|  |  | Quinic acid^57^ | 6508 |
|  |  | (-)-Gallocatechin^57^ | 9882981 |
|  |  | Neochlorogenic acid^57^ | 5280633 |
|  |  | Epigallocatechin^57^ | 72277 |
|  |  | Catechin^57^ | 9064 |
|  |  | Chlorogenic acid^57^ | 1794427 |
|  |  | Epicatechin^57^ | 72276 |
|  |  | Schaftoside^57^ | 442658 |
|  |  | Isoschaftoside^57^ | 3084995 |
|  |  | Quercitrin^57^ | 5280459 |
|  |  | Rosmarinic acid^57^ | 5281792 |
|  |  | Myricetin^57^ | 5281672 |
|  |  | Phlorizin^57^ | 6072 |
|  |  | Quercetin^57^ | 5280343 |
|  |  | Kaempferol^57^ | 5280863 |
|  |  | Betaine^57^ | 247 |
|  |  | p-Coumaric acid^57^ | 637542 |
|  |  | Primulanin^58^ | 44419565 |
|  |  | Lysikokianoside 1^58^ | 44593371 |
|  |  | Ardisiacrispin A^58^ | 10328746 |
|  |  | Ardisicrenoside B^58^ | 10373894 |
|  |  | Quercetin 3-glucoside^59^ | 5280804 |
|  |  | Kaempferol 3-galactoside^59^ | 5282149 |
|  |  | Myricetin 3-rhamnoside^59^ | 5352000 |
|  |  | Kaempferol 3-glucoside^59^ | 5282102 |
|  |  | 6,8-di-C-glucosylapigenin^59^ | 3084407 |
|  |  | Syringetin 3-galactoside^59^ | 44259488 |
| 21 | *Lysionotus pauciflorus* var. *pauciflorus* Maxim. | 4-Hydroxy-2',4'-dimethoxydihydrochalcone^60^ | 15549893 |
|  |  | Acerosin^60^ | 177696 |
|  |  | 5,7,3',4'-Tetrahydroxy-6,8-dimethoxyflavone^60^ | 5321859 |
|  |  | Nevadensin^60^ | 160921 |
|  |  | Ikarisoside B^60^ | 5481981 |
|  |  | 2''-O-Rhamnosylicariside II^60^ | 5318987 |
|  |  | Acteoside^60^ | 5281800 |
|  |  | 5,7-dihydroxy 6,8,4′-trimethoxyflavone^61^ | 160921 |
|  |  | p-Hydroxybenzoic acid^62^ | 135 |
|  |  | Vanillic acid^62^ | 8468 |
|  |  | Caffeic acid^62^ | 689043 |
|  |  | Lyoniresinol^62^ | 11711453 |
| 22 | *Mahonia duclouxiana* Gagnep. | Palmatine^63^ | 19009 |
|  |  | Jatrorrhizine^63^ | 72323 |
|  |  | Berberine^63^ | 2353 |
|  |  | Ocimene^63^ | 5281553 |
|  |  | Linalool^63^ | 6549 |
|  |  | 4-Terpineol^63^ | 11230 |
|  |  | alpha-Terpineol^63^ | 17100 |
|  |  | Geraniol^63^ | 637566 |
|  |  | beta-Damascenone^63^ | 5366074 |
|  |  | Alpha-Ionone^63^ | 5282108 |
|  |  | alpha-Farnesene^63^ | 5281516 |
|  |  | (-)-alpha-Gurjunene^63^ | 15560276 |
|  |  | Geranyl acetone^63^ | 1549778 |
|  |  | Ledol^63^ | 92812 |
|  |  | Hexadecane^63^ | 11006 |
|  |  | Patchoulic alcohol^63^ | 10955174 |
|  |  | Cedrol^63^ | 65575 |
|  |  | Phytone^63^ | 10408 |
|  |  | Hexadecanoic acid^63^ | 985 |
|  |  | 2,6,10,14- Tetramethyl octadecane^63^ | 521556 |
|  |  | Oleic acid methyl ester^63^ | 5364509 |
|  |  | Ethyl linoleate^63^ | 5282184 |
|  |  | Docosane^63^ | 12405 |
|  |  | Tetracosane^63^ | 12592 |
|  |  | Pentacosane^63^ | 12406 |
|  |  | Heptacosane^63^ | 11636 |
| 23 | *Marsdenia griffithii* Hook. f. | alpha-Pinene^64^ | 6654 |
|  |  | Sabinene^64^ | 18818 |
|  |  | beta-Pinene^64^ | 14896 |
|  |  | 3-Carene^64^ | 26049 |
|  |  | Ocimene^64^ | 5281553 |
|  |  | p-Cymene^64^ | 7463 |
|  |  | Limonene^64^ | 22311 |
|  |  | Terpinolene^64^ | 11463 |
|  |  | Linalool^64^ | 6549 |
|  |  | 1,3,8-Menthatriene^64^ | 176983 |
|  |  | Borneol^64^ | 64685 |
|  |  | Terpinen-4-ol^64^ | 11230 |
|  |  | Dihydrocarveol^64^ | 12072 |
|  |  | Carvone^64^ | 7439 |
|  |  | Mentha-2,8-diene,1-hydroperoxide^64^ | 6431185 |
|  |  | Piperitone^64^ | 6987 |
|  |  | Bornyl acetate^64^ | 6448 |
|  |  | Cuminol^64^ | 325 |
|  |  | Cyclohexene, 2-ethenyl-1,3,3-trimethyl-^64^ | 584957 |
|  |  | Perillol^64^ | 10819 |
|  |  | Thymol acetate^64^ | 68252 |
|  |  | Carvyl acetate^64^ | 7335 |
|  |  | Mentha-1,8-diene^64^ | 22311 |
|  |  | Bergamotene^64^ | 521569 |
|  |  | Himachalene^64^ | 11586487 |
|  |  | Aromandendrene^64^ | 91354 |
|  |  | Caryophyllene^64^ | 5281515 |
|  |  | alpha-Selinene^64^ | 10856614 |
|  |  | Germacrene^64^ | 9548705 |
|  |  | Muurolene^64^ | 12306047 |
|  |  | alpha-Cadinene^64^ | 12306048 |
|  |  | Palustrol^64^ | 110745 |
|  |  | Caryophyllene oxide^64^ | 1742210 |

**REFERENCES**

1. Al-Snafi, A. E. Chemical constituents, pharmacological effects and therapeutic importance of Hibiscus rosa-sinensis- A review. *Int. J. Pharm. Res.* **10**, 451–475 (2018).

2. Jadhav, V. M., Thorat, R. M., Kadam, V. J. & Sathe, N. S. Hibiscus rosa sinensis Linn – ‘“ Rudrapuspa ”’ : A Review. *J. Pharm. Res.* **2**, 1168–1173 (2009).

3. Ics, G. Y. N. A. M. 水平上下比に対する拡散波動場理論の適用性―地震動と微動の比較 川瀬博 1) 、松島信一 2) 、長嶋史明 3) 1). **11**, 1056–1063 (2014).

4. Salib, J. Y. *et al.* Polyphenolic compounds from flowers of hibiscus rosa-sinensis linn. and their inhibitory effect on alkaline phosphatase enzyme activity in vitro. *Zeitschrift fur Naturforsch. - Sect. C J. Biosci.* **66 C**, 453–459 (2011).

5. Chao Liu, X., Qi Bai, C., Zhi Liu, Q. & Long Liu, Z. Evaluation of nematicidal activity of the essential oil of Homalomena occulta (Lour.) Schott rhizome and its major constituents against Meloidogyne incognita (Kofoid and White) Chitwood. *J. Entomol. Zool. Stud. JEZS* **2**, 182–186 (2014).

6. Qu, Ở V Ƣ Ờ N *et al.* THÀNH PH Ầ N HÓA H Ọ C TINH D Ầ U LOÀI THIÊN NIÊN KI Ệ N ( HOMALOMENA OCCULTA ( LOUR .) SCHOTT ) VÀ TH Ầ N PH Ụ C ( HOMALOMENA PIERREANA ENGL .). 1236–1241 (2013).

7. Zhao, J., Wu, J. & Yan, F. L. A new sesquiterpenoid from the rhizomes of Homalomena occulta. *Nat. Prod. Res.* **28**, 1669–1673 (2014).

8. Fu, J., Dai, L., Lin, Z. & Lu, H. &lt;i&gt;Houttuynia cordata&lt;/i&gt; Thunb: A Review of Phytochemistry and Pharmacology and Quality Control. *Chin. Med.* **04**, 101–123 (2013).

9. Chou, S. C., Su, C. R., Ku, Y. C. & Wu, T. S. The constituents and their bioactivities of Houttuynia cordata. *Chem. Pharm. Bull.* **57**, 1227–1230 (2009).

10. Yang, L. & Jiang, J. G. Bioactive components and functional properties of Hottuynia cordata and its applications. *Pharm. Biol.* **47**, 1154–1161 (2009).

11. Dai, D. N., Thang, T. D., Ogunmoye, A., Eresanya, O. I. & Ogunwande, I. A. Chemical constituents of essential oils from the leaves of *Tithonia diversifolia*, *Houttuynia cordata* and *Asarum glabrum* grown in Vietnam. *Am. J. Essent. Oils Nat. Prod.* **2**, 17–21 (2015).

12. Ravi, S. *et al.* Phytochemical Screening , Antioxidant and Anticancer Potential of Imperata Cylindrica ( L .) Raeusch Against Human Breast Cancer Cell Line (MCF-7). **8**, 938–945 (2018).

13. Janosik, S. M. Phenolic acids from Imperata cylindrica (L.) Raeusch. var. Major (Nees) c.e. Hubb. *NASPA J.* **42**, 1 (2005).

14. Wang, Y., Shen, J. Z., Chan, Y. W. & Ho, W. S. Identification and growth inhibitory activity of the chemical constituents from Imperata cylindrica aerial part ethyl acetate extract. *Molecules* **23**, (2018).

15. Suzuki, M. *et al.* Plant growth inhibitory activity and active substances with allelopathic potential of cogongrass (Imperata cylindrica) rhizome. *Weed Biol. Manag.* **18**, 92–98 (2018).

16. Kalola, J., Shah, R., Patel, A., Lahiri, S. K. & Shah, M. B. Anti-inflammatory and immunomodulatory activities of Inula cappa roots (Compositae). *J. Complement. Integr. Med.* **14**, (2017).

17. Kalola, J., Shah, R. & Shah, M. Quantitative analysis of phytochemicals from Inula cappa roots. *J. Planar Chromatogr. - Mod. TLC* **29**, 336–340 (2016).

18. Priydarshi, R., Melkani, A. B., Mohan, L. & Pant, C. C. Terpenoid composition and antibacterial activity of the essential oil from Inula cappa (Buch-Ham. ex. D. Don) DC. *J. Essent. Oil Res.* **28**, 172–176 (2016).

19. Tang, W., Wei, X., Xu, H., Zeng, D. & Long, L. 13-Deoxyitol A, a new insecticidal isoryanodane diterpene from the seeds of Itoa orientalis. *Fitoterapia* **80**, 286–289 (2009).

20. Chai, X. Y. *et al.* Six insecticidal isoryanodane diterpenoids from the bark and twigs of Itoa orientalis. *Tetrahedron* **64**, 5743–5747 (2008).

21. Tang, W., Xu, H., Zeng, D. & Yu, L. The antifungal constituents from the seeds of Itoa orientalis. *Fitoterapia* **83**, 513–517 (2012).

22. Chai, X. Y. *et al.* Itosides J-N from Itoa orientalis and structure-anti-COX-2 activity relationship of phenolic glycosides. *J. Nat. Prod.* **71**, 814–819 (2008).

23. Chai, X. *et al.* Itosides A – I , New Phenolic Glycosides from Itoa orientalis in Chinese , have been used in folk medicine for the treatment of rheumatism , injuries. **90**, 2176–2185 (2007).

24. Luo, B. *et al.* *Characterization and immunological activity of polysaccharides from Ixeris polycephala*. *International Journal of Biological Macromolecules* vol. 113 (Elsevier B.V, 2018).

25. Deng, S. & Li, X. Inhibition by Jasminum nudiflorum Lindl. leaves extract of the corrosion of aluminium in HCl solution. *Corros. Sci.* **64**, 253–262 (2012).

26. Jain, Akash; Sharma, Rishu; Kumar, Ashok; Sharma, S. JASMINUM SPECIES: AN OVERVIEW. *Int. J. Institutional Pharm. Life Sci.* **1**, 251–266 (2011).

27. Takenaka, Y., Tanahashi, T., Taguchi, H., Nagakura, N. & Nishi, T. Nine new secoiridoid glucosides from Jasminum nudiflorum. *Chem. Pharm. Bull.* **50**, 384–389 (2002).

28. LIU, Y.-B. *et al.* A Review of Triterpenoids and Their Pharmacological Activities from Genus Kadsura. *Digit. Chinese Med.* **1**, 247–258 (2018).

29. Shehla, N. *et al.* New dibenzocyclooctadiene lignan from stems of Kadsura heteroclita. *Nat. Prod. Res.* **0**, 1–10 (2020).

30. Wang, C. F. *et al.* Chemical composition and toxicities of essential oil of Illicium fragesii fruits against Sitophilus zeamais. *African J. Biotechnol.* **10**, 18179–18184 (2011).

31. AL-SNAFI, A. E. a Review on Lawsonia Inermis: a Potential Medicinal Plant. *Int. J. Curr. Pharm. Res.* **9**, 1–13 (2019).

32. Verma, R. S., Padalia, R. C. & Chauhan, A. Compositional variation in the essential oils of vegetative and reproductive parts of laggera crispata (Vahl) hepper & wood. *Natl. Acad. Sci. Lett.* **36**, 447–451 (2013).

33. Miao, L. L., Zhou, Q. M., Peng, C., Liu, Z. H. & Xiong, L. Leonurus japonicus (Chinese motherwort), an excellent traditional medicine for obstetrical and gynecological diseases: A comprehensive overview. *Biomed. Pharmacother.* **117**, 109060 (2019).

34. Xiong, L. *et al.* Chemical composition and antibacterial activity of essential oils from different parts of Leonurus japonicus houtt. *Molecules* **18**, 963–973 (2013).

35. Shang, X., Pan, H., Wang, X., He, H. & Li, M. Leonurus japonicus Houtt.: Ethnopharmacology, phytochemistry and pharmacology of an important traditional Chinese medicine. *J. Ethnopharmacol.* **152**, 14–32 (2014).

36. Zhang, C. *et al.* Two new phenylpropanoid glycosides from the aerial parts of Lespedeza cuneata. *Acta Pharm. Sin. B* **6**, 564–567 (2016).

37. Min, J. Y. & Shim, S. H. Chemical constituents from Lespedeza cuneata G. Don (Leguminosae). *Biochem. Syst. Ecol.* **66**, 293–296 (2016).

38. Cai-peng,X.; Ying,W.; Qian,W.; Yang,F.; Hui,P.; Min,X.; Hai-zhou, L. Chemical Constituents from Lysteria formosa. *Nat Prod Res Dev* **29**, 58–62 (2017).

39. Lobstein, A. *et al.* Bioactive compounds from Leycesteria formosa. *Pharmazie* **57**, 431–432 (2002).

40. Yang, J. B. *et al.* New dimeric phthalides from Ligusticum sinense Oliv cv. Chaxiong. *J. Asian Nat. Prod. Res.* **16**, 747–752 (2014).

41. Wei, Q. *et al.* Bioactive phthalides from Ligusticum sinense Oliv cv. Chaxiong. *Fitoterapia* **93**, 226–232 (2014).

42. Yuanzheng, H.; Fading, P. Studies on the Chemical Components of Leaf Essential Oils from Ligusticum Sinense CV. Chuanxiong. *Plant Divers.* **10**, 1–3 (1998).

43. Yang, Y., Olatunde, O. Z., Yong, J. & Lu, C. Progress of Chemical Components and Biological Activities of Lonicera Maackii. *Mathews J. Cancer Sci.* **3**, 1–5 (2018).

44. Yong, J., Lu, C. & Huang, S. Chemical constituents of Lonicera maackii. *Chem. Nat. Compd.* **50**, 945–947 (2014).

45. Li, Y. *et al.* Volatile Organic Compounds Emissions from Luculia pinceana Flower and Its Changes at Different Stages of Flower Development. *Molecules* **21**, 1–10 (2016).

46. Shendge, P. N. & Belemkar, S. Therapeutic potential of luffa acutangula: A review on its traditional uses, phytochemistry, pharmacology and toxicological aspects. *Front. Pharmacol.* **9**, (2018).

47. Manikandaselvi, S., Vadivel, V. & Brindha, P. Review on Luffa acutangula L.: Ethnobotany, phytochemistry, nutritional value and pharmacological properties. *Int. J. Curr. Pharm. Rev. Res.* **7**, 151–155 (2016).

48. Shirish, P. et al. Pharmacological Review of “ Luffa acutangula ( L ) Roxb. 1–8 (2018).

49. Anitha, J. & Miruthula, S. Traditional Medicinal Uses, Phytochemical Profile And Pharmacological Activities Of Luffa Acutangula Linn. *Int. J. Pharmacogn.* **1**, 174–183 (2014).

50. Skenderidis, P. *et al.* Chemical properties, fatty-acid composition, and antioxidant activity of Goji berry (Lycium barbarum l. and Lycium Chinense mill.) fruits. *Antioxidants* **8**, (2019).

51. Kruczek, A., Ochmian, I., Małkiewicz, M. K.-, Szczecin, T. & Street, S. COMPARISON OF MORPHOLOGICAL ANTIDIABETIC AND ANTIOXIDANT PROPERTIES OF GOJI FRUITS. *Sciendo* **24**, 1–14 (2020).

52. Mocan, A. *et al.* Polyphenolic content, antioxidant and antimicrobial activities of Lycium barbarum L. And Lycium chinense Mill. Leaves. *Molecules* **19**, 10056–10073 (2014).

53. Terauchi,M.; Kanamori,H.; Nobuso,M.; Fukuda,S.; Yahara,S.; Yamasaki, K. Antimicrobial Components in Leaves of Lycium chinense Mill. *J. Food Hyg. Soc. Japan* **39**, 399–405 (1998).

54. Sultana, S., Ali, M. & Naquvi, K. J. Aliphatic esters and fatty acids from the fruits of Lycium chinense miller. **2**, 42–46 (2015).

55. Yang, Y. N. *et al.* Nine new compounds from the root bark of Lycium chinense and their α-glucosidase inhibitory activity. *RSC Adv.* **7**, 805–812 (2017).

56. Gao, F., Zhao, D. & Deng, J. New flavonoids from lysimachia christinae hance. *Helv. Chim. Acta* **96**, 985–989 (2013).

57. Shim, K. S., Hwang, Y. H., Jang, S. A., Kim, T. & Ha, H. Water extract of lysimachia christinae inhibits trabecular bone loss and fat accumulation in ovariectomized mice. *Nutrients* **12**, 1–14 (2020).

58. Tian, L. J., Yang, N. Y. & Chen, W. Q. Triterpene saponins from Lysimachia christinae. *J. Asian Nat. Prod. Res.* **10**, 265–270 (2008).

59. Yasukawa, K. & Takido, M. Flavonoid glycosides from Lysimachiae herba and Lysimachia christinae var. typica. *Planta Med.* **59**, 578 (1993).

60. Luo, W. *et al.* A new flavonoid glycoside from Lysionotus pauciflorus. *Nat. Prod. Commun.* **11**, 621–622 (2016).

61. Wen, Y. *et al.* Preparative enrichment and purification of nevadensin from Lysionotus pauciflorus using macroporous resins. *Sep. Sci. Technol.* **6395**, 339–347 (2016).

62. Liang, B. A New Sesquiterpene Glucoside from Lysionotus pauciflorus. *Nat. Prod. Commun.* **9**, 1025–1026 (2014).

63. Li, Y., Kong, D. & Wu, H. Comparison of the alkaloid content and essential oil composition of Mahonia species as measured by HPLC and GC–MS methods. *Rev. Bras. Bot.* **41**, 765–774 (2018).

64. Min, Y., Wang, Z., Fang, A., Zhan, L. & Xu, C. Chemical Composition and Antimicrobial Activity of Marsdenia griffithii Hook. f. Essential Oil. *Med. Biopharm.* 956–962 (2013).
