# Supplementary material for: Plant-derived compounds effectively inhibit the main protease of SARS-CoV-2: An in silico approach
Source: PLoS One. 2022 Aug 23;17(8):e0273341. doi: 10.1371/journal.pone.0273341 (PMC9398018; doi:10.1371/journal.pone.0273341)
Supplement: S2 Table — (DOCX) [file pone.0273341.s002.docx]

S2 Table: Chemical name and pubchem CID of different phytochemicals retrived from different plants.

| **SERIAL NO.** | **PLANT NAME** | **CHEMICAL NAME** | **PUBCHEM CID** |
| --- | --- | --- | --- |
| 01 | *Lycopodium japonicum* Thunb. ex Murray | Lycopodine^1^ | 5462445 |
|  |  | Lycodoline^1^ | 12312555 |
|  |  | Clavolonine^1^ | 21581110 |
|  |  | Acetylfawcettiine^1^ | 101500246 |
|  |  | Lycofawcine^1^ | 5462444 |
|  |  | Lycoposerramine M^1^ | 11129147 |
|  |  | Lycoposerramine L^1^ | 11054525 |
|  |  | Serratezomine C^1^ | 10636398 |
|  |  | Lycoposerramine G^1^ | 10956867 |
|  |  | 12-epilycodoline^1^ | 621853 |
|  |  | Anhydrolycodoline^1^ | 102442792 |
|  |  | Lycoposerramine K^1^ | 10901401 |
|  |  | Gnidioidine^1^ | 122234718 |
|  |  | Lucidioline^1^ | 21576177 |
|  |  | Flabelline^1^ | 101289809 |
|  |  | Huperzine E^1^ | 102588705 |
|  |  | Lycoposerramine F^1^ | 101751330 |
|  |  | Miyoshianine C^1^ | 102026861 |
|  |  | Lycodine^1^ | 5462443 |
|  |  | alpha-Obscurine^1^ | 5462446 |
|  |  | beta-Obscurine^1^ | 5460546 |
|  |  | Huperzinine^1^ | 195296 |
|  |  | Fawcettimine^1^ | 442475 |
|  |  | Lycopoclavamine A^1^ | 101961408 |
|  |  | Fawcettidine^1^ | 442473 |
|  |  | Phlegmariurine B^1^ | 179734 |
|  |  | Lycojapodine A^1^ | 25258213 |
|  |  | 8-deoxy-13-dehydroserratinine^1^ | 73357774 |
|  |  | Lycoflexine^1^ | 442486 |
|  |  | Lycojaponicumin E^1^ | 57342090 |
|  |  | Lycoflexine N-oxide^1^ | 101961411 |
|  |  | Palhinine A^1^ | 46933830 |
|  |  | Lycojaponicumin D^1^ | 102306584 |
|  |  | Lycojaponicumin A^1^ | 60145017 |
|  |  | Lycojaponicumin B^1^ | 102292237 |
|  |  | Lycojaponicumin C^1^ | 60145172 |
|  |  | Isopalhinine A^1^ | 71747141 |
|  |  | Lycocernuine^1^ | 442481 |
|  |  | Serratenediol^1^ | 164947 |
|  |  | Diepiserratenediol^1^ | 21672659 |
|  |  | 3-epilycoclavanol^1^ | 146157076 |
|  |  | Lycernuic acid A^1^ | 636674 |
|  |  | Phlegmaric acid^1^ | 101316831 |
|  |  | Lycoclavanol^1^ | 91895419 |
|  |  | Japonicumin A^1^ | 16080349 |
|  |  | Lycernuic ketone C^1^ | 10863674 |
|  |  | Japonicumin B^1^ | 16080347 |
|  |  | Tohogenol^1^ | 101528287 |
|  |  | Japonicumin C^1^ | 101408072 |
|  |  | Lycopodiin A^1^ | 11328987 |
|  |  | Alpha-onocerin^1^ | 11453544 |
|  |  | alpha-Onoceradienedione^1^ | 12313728 |
|  |  | Betulin^1^ | 72326 |
|  |  | Alkaloid L-23^2^ | 621853 |
| 02 | *Marsdenia tenacissima* (Roxb.) Moon | Tenacigenoside A^3^ | 91885077 |
|  |  | Tenacigenoside B^3^ | 102153486 |
|  |  | Tenacigenoside C^3^ | 102153487 |
|  |  | Tenacigenoside D^3^ | 102153488 |
|  |  | Tenacigenin A^3^ | 101377895 |
|  |  | Tenacigenin B^3^ | 12137734 |
|  |  | Tenacissoside A^3^ | 102186880 |
|  |  | Tenacissoside F^3^ | 91895269 |
|  |  | Tenacissoside G^3^ | 44561398 |
|  |  | Marsdenoside H^3^ | 101743841 |
|  |  | Marsdenoside D^3^ | 101743839 |
|  |  | Tenacissoside H^4^ | 75412560 |
|  |  | Tenacissoside I^4^ | 91973812 |
|  |  | Marsdenoside A^4^ | 11228220 |
|  |  | Marsdenoside B^4^ | 101743838 |
|  |  | Marsdenoside C^4^ | 11320433 |
|  |  | Marsdenoside E^4^ | 101743840 |
|  |  | Marsdenoside F^4^ | 11343196 |
|  |  | Marsdenoside G^4^ | 11274200 |
|  |  | Marsdenoside J^4^ | 101377893 |
|  |  | Marsdenoside K^4^ | 101377894 |
|  |  | Tenacissoside B^4^ | 102186879 |
|  |  | Marsdenoside M^4^ | 102421940 |
|  |  | Marsdenoside L^4^ | 102421939 |
|  |  | Marstenacisside A1^4^ | 101902129 |
|  |  | Marstenacisside A4^4^ | 129908835 |
|  |  | Marstenacisside A5^4^ | 129908867 |
|  |  | Marstenacisside A7^4^ | 129908695 |
|  |  | Marstenacisside B2^4^ | 129909235 |
|  |  | Marstenacisside B5^4^ | 129909102 |
|  |  | Marstenacisside B6^4^ | 129909035 |
|  |  | Marstenacisside B8^4^ | 129909056 |
|  |  | Drevogenin Q^4^ | 101277285 |
|  |  | Marstenacisside A2^4^ | 101902130 |
|  |  | Marstenacisside A6^4^ | 129909057 |
|  |  | Marstenacisside B1^4^ | 101902132 |
|  |  | Marstenacisside B3^4^ | 129909236 |
|  |  | Marstenacisside B4^4^ | 129908696 |
|  |  | Marstenacisside B7^4^ | 129909055 |
|  |  | Marstenacisside B9^4^ | 129908907 |
|  |  | Dihydrosarcostin^4^ | 15558996 |
|  |  | Dresgenin^4^ | 14281864 |
|  |  | Marstenacigenin A^4^ | 101694474 |
|  |  | Marstenacigenin B^4^ | 101694475 |
|  |  | Tenacissoside L^4^ | 11657939 |
|  |  | Tenacissoside M^4^ | 11622059 |
|  |  | Marsdekoiside B^4^ | 3083306 |
|  |  | Cissogenin^4^ | 102117168 |
|  |  | Tenasogenin^4^ | 101277354 |
|  |  | Isodrevogenin-P^4^ | 101760032 |
|  |  | Tenacigenin D^4^ | 122219079 |
| 03 | *Mimosa pudica* Linn. | N-dl-Alanylglycine^5^ | 79094 |
|  |  | dl-Alanyl-dl Valine^5^ | 137276 |
|  |  | d-Alanin^5^ | 71080 |
|  |  | 2-methylamino-N- phenylacetamide^5^ | 541846 |
|  |  | Meglumine^5^ | 8567 |
|  |  | 9, 12-Octadecadienoic acid (Z, Z), methyl ester^5^ | 6443213 |
|  |  | Quercetin-7-rhamnoside^5^ | 5748601 |
|  |  | Acacetin-7-rutinoside^5^ | 44257888 |
|  |  | D-Pinitol^5^ | 164619 |
|  |  | L-Mimosine^5^ | 440473 |
|  |  | Mimosinamine^5^ | 94477 |
|  |  | P-coumaric acid^5^ | 637542 |
|  |  | Ethanol 2-methoxy acetate^6^ | 8054 |
|  |  | Phthalic acid dioctyl ester^6^ | 8343 |
|  |  | Methyl octyl ester^6^ | 6424476 |
|  |  | 3-Pinanone^6^ | 11038 |
|  |  | α-Linolenic acid^6^ | 5280934 |
|  |  | Phytol^6^ | 5280435 |
|  |  | n-Hexadecanoic acid^6^ | 985 |
|  |  | Dibutyl phthalate^6^ | 3026 |
|  |  | 7,9‑Di‑tert‑butyl‑1‑oxaspiro (4,5) deca‑6,9‑diene‑2,8‑dione^6^ | 545303 |
|  |  | 1, 2‑Benzenedicarboxylic acid, bis (2‑methylpropyl) ester^6^ | 6782 |
|  |  | Tetradecanoic acid^6^ | 11005 |
|  |  | 1‑Pentadecene^6^ | 25913 |
|  |  | Dodecanoic acid^6^ | 3893 |
|  |  | 1,6,10‑Dodecatrien‑3‑ol, 3,7,11‑trimethyl^6^ | 67848531 |
|  |  | Phenol, 2,4‑bis (1,1‑dimethylethyl)‑^6^ | 93344 |
|  |  | 3‑Buten‑2‑one, 4‑(2,6,6‑trimethyl‑1‑cyclohexen‑1‑yl)^6^ | 9562678 |
|  |  | Phenol, 2-methoxy-3-(2-propenyl)-^6^ | 596373 |
|  |  | 2‑Methoxy‑4‑vinylphenol^6^ | 332 |
|  |  | Indole^6^ | 798 |
|  |  | Cinnamaldehyde^6^ | 637511 |
|  |  | 2,3-Dihydrobenzofuran^6^ | 10329 |
|  |  | Benzene methanol^6^ | 21927730 |
|  |  | 3‑Hexen‑1‑ol^6^ | 6441969 |
|  |  | Mimosine^7^ | 3862 |
|  |  | d – Xylose^7^ | 135191 |
|  |  | d- Glucuronic acid^7^ | 44146331 |
|  |  | Quercetin^7^ | 5280343 |
|  |  | Jasmonic acid^7^ | 5281166 |
|  |  | Turgorin^7^ | 442990 |
|  |  | Naringin^7^ | 442428 |
|  |  | Beta-Sitosterol^7^ | 222284 |
|  |  | dl-Alanyl-dl-Valine^7^ | 137276 |
|  |  | 11, 13-Eicosadienoic acid^7^ | 25200976 |
|  |  | 1-Octanamine^7^ | 8143 |
|  |  | 1. Butanamine^7^ | 118823 |
|  |  | 7 -3 -4 - trihydroxy-3,8-dimethoxyflavone^8^ | 44258704 |
|  |  | Magnesium potassium trans-aconitate^8^ | 100982879 |
|  |  | 2-hydroxymethyl-chroman 4-one^8^ | 14089213 |
|  |  | Betulinic acid^8^ | 64971 |
|  |  | Stigmasterol^8^ | 5280794 |
| 04 | *Mirabilis jalapa* Linn. | 3, 3’-Methylenebis(4- hydroxycoumarin)^9^ | 54676038 |
|  |  | Laminaribiitol^9^ | 12302626 |
|  |  | 3-(4-(dimethylamino) cinnamoyl) 4-hydroxycoumarin^9^ | 54707106 |
|  |  | Mirabijalone A^9^ | 10948317 |
|  |  | Mirabijalone B^9^ | 11067832 |
|  |  | Mirabijalone C^9^ | 11071220 |
|  |  | Mirabijalone D^9^ | 11013288 |
|  |  | Boeravinone C^9^ | 13940641 |
|  |  | Boeravinone F^9^ | 12004175 |
|  |  | 9-O-methyl-4-hydroxyboeravinone B^9^ | 487168 |
|  |  | 1, 2, 3, 4-tetrahydro-1-methylisoquinoline-7, 8-diol^9^ | 10261676 |
|  |  | Campesterol^9^ | 173183 |
|  |  | Daucosterol^9^ | 5742590 |
|  |  | Indicaxanthin^9^ | 6096870 |
|  |  | Isobetanin^9^ | 6325438 |
|  |  | 6-methoxyboeravinone^9^ | 487169 |
|  |  | n-Dotriacontane^9^ | 11008 |
|  |  | n Nonacosane^9^ | 12409 |
|  |  | n Pentacosane^9^ | 12406 |
|  |  | n-Triacontane^9^ | 12535 |
|  |  | alpha-Amyrine^10^ | 73170 |
|  |  | Oleanolic acid^10^ | 10494 |
|  |  | Beta sistosterol^10^ | 222284 |
|  |  | Trigonellin^10^ | 5570 |
|  |  | Betanin^10^ | 12300103 |
|  |  | Brassicasterol^10^ | 5281327 |
|  |  | Betalanic acid^10^ | 6123097 |
|  |  | Tartaric acid^10^ | 875 |
| 05 | *Morus alba* L. | Isoquercetin^11^ | 5280804 |
|  |  | Morin hydrate^11^ | 16219651 |
|  |  | Quercetin^11^ | 5280343 |
|  |  | Cyanidin-3-glucoside^12^ | 197081 |
|  |  | Cyanidin-3-rutinoside^12^ | 14034151 |
|  |  | Rutin^12^ | 5280805 |
|  |  | Quercetin 3-O-galactoside^12^ | 5281643 |
|  |  | Myricetin^12^ | 5281672 |
|  |  | Kaempferol^12^ | 5280863 |
|  |  | Kaempferol 3-O-glucoside^12^ | 5282102 |
|  |  | Kaempferol 3-O-rutinoside^12^ | 5318767 |
|  |  | Catechin^12^ | 9064 |
|  |  | Epigallocatechin Gallate^12^ | 65064 |
|  |  | Epicatechin^12^ | 72276 |
|  |  | Procyanidin B1^12^ | 11250133 |
|  |  | Procyanidin B2^12^ | 122738 |
|  |  | Chlorogenic acid^12^ | 1794427 |
|  |  | Ferulic acid^12^ | 445858 |
|  |  | p-Coumaric acid^12^ | 637542 |
|  |  | o-Coumaric acid^12^ | 637540 |
|  |  | Cinnamic acid^12^ | 444539 |
|  |  | Caffeic acid^12^ | 689043 |
|  |  | Gallic acid^12^ | 370 |
|  |  | p-Hydroxybenzoic acid^12^ | 135 |
|  |  | Syringic acid^12^ | 10742 |
|  |  | Protocatechuic acid^12^ | 72 |
|  |  | Vanillic acid^12^ | 8468 |
|  |  | Scopolin^13^ | 439514 |
|  |  | Skimmin^13^ | 99693 |
|  |  | Roseoside II^13^ | 9930064 |
|  |  | Benzyl D-glucopyranoside^13^ | 11076492 |
|  |  | 2′,7-dihydroxy-4′-methoxy-8- prenylflavan^14^ | 85228900 |
|  |  | Brosimine B^14^ | 21604819 |
|  |  | 2′,4′-dihydroxy-7′- methoxy-8-prenylflavan^14^ | 10759602 |
|  |  | Morachalcone A^14^ | 9862769 |
|  |  | Isobavachalcone^14^ | 5281255 |
|  |  | Moracin M^14^ | 185848 |
|  |  | Moracin C^14^ | 155248 |
|  |  | 7-hydroxycoumarin^14^ | 5281426 |
| 06 | *Munronia pinnata* (Wall.) W. Theobald | Hydroxylamine^15^ | 787 |
|  |  | 2-Heptadecanone^15^ | 18027 |
|  |  | 2-Nonadecanone^15^ | 69423 |
|  |  | Phytol^15^ | 5280435 |
|  |  | Heptadecanoic acid,16- methyl-methyl ester^15^ | 110444 |
|  |  | Cholesterol^15^ | 5997 |
| 07 | *Myrica rubra* (Lour.) Siebold et Zucc. | Myricanone^16^ | 161748 |
|  |  | Myricanol^16^ | 161779 |
|  |  | Myricetin^16^ | 5281672 |
|  |  | Myricitrin^16^ | 5281673 |
|  |  | (−)-Epicathechin^16^ | 122738 |
|  |  | Rhoiptelenol^16^ | 102304204 |
|  |  | Ursolic acid^16^ | 64945 |
|  |  | Cyanidin-3-glucoside^17^ | 197081 |
|  |  | Quercetin^17^ | 5280343 |
|  |  | Quercetin-3-O-glucoside^17^ | 5280804 |
|  |  | Quercetin-3-O-rhamnoside^17^ | 5280459 |
|  |  | Quercetin-3-O-rutinoside^17^ | 5280805 |
|  |  | Gallic acid^17^ | 370 |
|  |  | Protocatechuic acid^17^ | 72 |
|  |  | p-Hydroxybenzoic acid^17^ | 135 |
|  |  | P coumaric acid^17^ | 637542 |
|  |  | Caffeic acid^17^ | 689043 |
|  |  | Ferulic acid^17^ | 445858 |
|  |  | (+)-S-myricanol^17^ | 14059610 |
|  |  | Myricanene A^17^ | 102304203 |
|  |  | Prodelphinidin B-2 3,3′-di-O-gallate^17^ | 467306 |
|  |  | 2,2-diphenyl-1-picrylhydrazyl^18^ | 74358 |
|  |  | Cyanidin-3–O-galactoside^18^ | 441699 |
|  |  | Cyanidin-3–O-glucoside^18^ | 441667 |
|  |  | Pelargonidin-3–O-glucoside^18^ | 12302249 |
|  |  | Peonidin-3-O-glucoside^18^ | 14311151 |
|  |  | Quercetin-3-O-galactoside^18^ | 5281643 |
|  |  | Kaempferol-3–O-galactoside^18^ | 5462193 |
|  |  | Kaempferol-3–O-glucoside^18^ | 5282102 |
|  |  | Rutin^19^ | 5280805 |
|  |  | Hyperin^19^ | 5281643 |
|  |  | Kaempferol-3-O-rutinoside^19^ | 5318767 |
|  |  | Dihydromyricetin^19^ | 161557 |
|  |  | Morin^19^ | 5281670 |
|  |  | Isorhamnetin^19^ | 5281654 |
|  |  | Naringenin^19^ | 932 |
|  |  | Luteolin^19^ | 5280445 |
|  |  | DL-Malic acid^19^ | 525 |
|  |  | Citric acid^19^ | 311 |
|  |  | Salicylic acid^19^ | 338 |
|  |  | 2,4-Dihydroxybenzoic acid^19^ | 1491 |
|  |  | Cyanidin^20^ | 128861 |
|  |  | Ellagic acid^20^ | 5281855 |
|  |  | Quercetin deoxyhexoside^20^ | 56664758 |
| 08 | *Nepeta cataria* Linn. | α-Pinene^21^ | 6654 |
|  |  | Sabinene^21^ | 18818 |
|  |  | β-Pinene^21^ | 14896 |
|  |  | 1-Cyclohexen-1-yl-methyl ketone^21^ | 13612 |
|  |  | Triplal^21^ | 93375 |
|  |  | Thymol^21^ | 6989 |
|  |  | trans Caryophyllene^21^ | 5354499 |
|  |  | α-Humulene^21^ | 5281520 |
|  |  | 11-Dodecenol^21^ | 520725 |
|  |  | Spathulenol^21^ | 92231 |
|  |  | Caryophyllene oxide^21^ | 1742210 |
|  |  | alpha-Fenchene^22^ | 28930 |
|  |  | alpha-Fellandrene^22^ | 7460 |
|  |  | alpha-Terpinene^22^ | 7462 |
|  |  | 1,8-Cineol^22^ | 2758 |
|  |  | cis-beta-Ocimene^22^ | 5320250 |
|  |  | Beta-trans-Ocimene^22^ | 5281553 |
|  |  | Terpinene-4-acetate^22^ | 20960 |
|  |  | Linalool^22^ | 6549 |
|  |  | Terpinolene^22^ | 11463 |
|  |  | Terpinene-4-ol^22^ | 11230 |
|  |  | Caran-3 beta-ol^22^ | 142315 |
|  |  | E-Geraniol^22^ | 637566 |
|  |  | Bornyl acetate^22^ | 6448 |
|  |  | beta-Bourbonene^22^ | 62566 |
|  |  | alpha-Copaene^22^ | 19725 |
|  |  | Geranyl acetate^22^ | 1549026 |
|  |  | beta-Caryophyllene^22^ | 5281515 |
|  |  | alpha-trans-Bergamotene^22^ | 86608 |
|  |  | alpha-Humulene^22^ | 5281520 |
|  |  | Germacrene D^22^ | 5317570 |
|  |  | gamma-Cadinene^22^ | 92313 |
|  |  | Santalol^22^ | 5368798 |
|  |  | Caffeic Acid^23^ | 689043 |
|  |  | Rosmarinic Acid^23^ | 5281792 |
|  |  | Luteolin^23^ | 5280445 |
|  |  | Apigenin^23^ | 5280443 |
| 09 | *Oenanthe javanica* (Bl.) DC. | Alpha-Pinene^24^ | 6654 |
|  |  | Camphene^24^ | 6616 |
|  |  | beta-Pinene^24^ | 14896 |
|  |  | beta-Myrcene^24^ | 31253 |
|  |  | alpha-Phellandrene^24^ | 7460 |
|  |  | alpha-Terpinene^24^ | 7462 |
|  |  | p-Cymene^24^ | 7463 |
|  |  | Limonene^24^ | 22311 |
|  |  | (E)-beta-ocimene^24^ | 5281553 |
|  |  | (Z)-beta-ocimene^24^ | 5320250 |
|  |  | γ-Terpinene^24^ | 7461 |
|  |  | alpha-Terpinolene^24^ | 11463 |
|  |  | Limonene oxide^24^ | 91496 |
|  |  | Bicycloelemene^24^ | 56842786 |
|  |  | alpha-Copaene^24^ | 19725 |
|  |  | beta-Elemene^24^ | 6918391 |
|  |  | (E)-caryophyllene^24^ | 5281515 |
|  |  | alpha-Humulene^24^ | 5281520 |
|  |  | (E)-beta-farnesene^24^ | 5281517 |
|  |  | alpha-Amorphene^24^ | 12306052 |
|  |  | Germacrene D^24^ | 5317570 |
|  |  | beta-Selinene^24^ | 442393 |
|  |  | alpha-Selinene^24^ | 10856614 |
|  |  | (Z,E)-alpha-farnesene^24^ | 5362889 |
|  |  | (E,E)-alpha-farnesene^24^ | 5281516 |
|  |  | δ-Cadinene^24^ | 6432404 |
|  |  | Neophytadiene^24^ | 10446 |
|  |  | (Z)-2-pentenol^24^ | 5364919 |
|  |  | (Z)-3-hexenol^24^ | 5281167 |
|  |  | (E)-2-hexenol^24^ | 5318042 |
|  |  | 2-Heptanol^24^ | 10976 |
|  |  | Linalool^24^ | 6549 |
|  |  | Borneol^24^ | 64685 |
|  |  | Terpinen-4-ol^24^ | 11230 |
|  |  | alpha-Terpineol^24^ | 17100 |
|  |  | p-Cymen-8-ol^24^ | 14529 |
|  |  | Nerolidol^24^ | 5284507 |
|  |  | (E)-farnesol^24^ | 445070 |
|  |  | alpha-Cadinol^24^ | 10398656 |
|  |  | Falcarinol^24^ | 5281149 |
|  |  | Hexanal^24^ | 6184 |
|  |  | (Z)-3-hexenal^24^ | 643941 |
|  |  | (E)-2-hexenal^24^ | 5281168 |
|  |  | (E,E)-2,4-hexadienal^24^ | 637564 |
|  |  | Phenylacetaldehyde^24^ | 998 |
|  |  | Nonanal^24^ | 31289 |
|  |  | 2,6-Nonadienal^24^ | 11196 |
|  |  | (E)-2-nonenal^24^ | 5283335 |
|  |  | Caffeic acid^25^ | 689043 |
|  |  | Chlorogenic acid^25^ | 1794427 |
|  |  | p-Coumaric acid^25^ | 637542 |
|  |  | Isochlorogenic acid A^25^ | 6474310 |
|  |  | Isochlorogenic acid B^25^ | 5281780 |
|  |  | Quercetin^25^ | 5280343 |
|  |  | Rutin^25^ | 5280805 |
|  |  | Hyperoside^25^ | 5281643 |
|  |  | Kaempferol-3-O-rutinoside^25^ | 5318767 |
|  |  | Astragalin^25^ | 5282102 |
|  |  | Isorhamnetin^25^ | 5281654 |
|  |  | Narcissoside^25^ | 5481663 |
|  |  | Luteoloside^25^ | 5280637 |
|  |  | Apigenin^25^ | 5280443 |
|  |  | Oenanthoside A^26^ | 54446769 |
|  |  | Ferulic acid^26^ | 445858 |
|  |  | p-Coumaric acid^26^ | 637542 |
|  |  | 4-hydroxyphenethyl trans-ferulate^26^ | 637308 |
|  |  | Falcarinol^26^ | 5281149 |
|  |  | Falcarindiol^26^ | 5281148 |
|  |  | Persicarin^26^ | 5487766 |
|  |  | Isoquercitrin^26^ | 5280804 |
|  |  | Lutein^26^ | 5281243 |
|  |  | γ-tocopherol^26^ | 92729 |
|  |  | Incensole^26^ | 44583885 |
|  |  | α-Copaene^26^ | 19725 |
|  |  | Bornyl acetate^26^ | 6448 |
|  |  | 4,11,11-trimethyl-8-methylene-[1R-(1R,4Z,9S)]- bicyclo-[7.2.0] undec-4-ene^27^ | 5322111 |
|  |  | 6-butyl-1,4-cycloheptadiene^27^ | 556470 |
|  |  | Eudesma-4(14),11-diene^27^ | 442393 |
| 10 | *Opuntia dillenii* (Ker Gawl.) Haw. | Indicaxanthin^28^ | 6096870 |
|  |  | 14,15-dehydrobetanin^28^ | 102502571 |
|  |  | Gomphrenin I^28^ | 6096868 |
|  |  | Isogomphrenin I^28^ | 101105495 |
|  |  | Neobetanin^28^ | 102401026 |
|  |  | Quercetin 3-O-glucoside^28^ | 5280804 |
|  |  | Kaempferol 3-O-arabinoside^28^ | 5481882 |
|  |  | Isorhamnetin 3-O-glucoside^28^ | 5318645 |
|  |  | Isorhamnetin 3-O-rutinoside^28^ | 5481663 |
|  |  | Gallic acid^28^ | 370 |
|  |  | Vanillic acid^28^ | 8468 |
|  |  | Ethyl 3,4-dihydroxybenzoate^28^ | 77547 |
|  |  | Sinapic acid^28^ | 637775 |
|  |  | Ferulic acid^28^ | 445858 |
|  |  | p-Coumaric acid^28^ | 637542 |
|  |  | Palmitic acid^28^ | 985 |
|  |  | oleic acid^28^ | 445639 |
|  |  | Stearic acid^28^ | 5281 |
|  |  | Arachidic acid^28^ | 10467 |
|  |  | myristic acid^28^ | 11005 |
|  |  | Taurine^28^ | 1123 |
|  |  | Betanin^29^ | 12300103 |
|  |  | Isobetanin^29^ | 6325438 |
|  |  | Isorhamnetin-3-glucuronide^29^ | 5491630 |
|  |  | Tryptophan-betaxanthin^29^ | 136728070 |
|  |  | Portulacaxanthin II^29^ | 135438597 |
|  |  | 3-O-methyl quercetin^30^ | 5280681 |
|  |  | Kaempferol^30^ | 5280863 |
|  |  | Kaempferide^30^ | 5281666 |
|  |  | Quercetin^30^ | 5280343 |
|  |  | Isorhamnetin^30^ | 5281654 |
|  |  | β-sitosterol^30^ | 222284 |
|  |  | Opuntisterol^30^ | 101429604 |
|  |  | Opuntisteroside^30^ | 101429605 |
|  |  | Taraxerol^30^ | 92097 |
|  |  | Friedelin^30^ | 91472 |
|  |  | Methyl linoleate^30^ | 5284421 |
|  |  | 7-oxositosterol^30^ | 146157937 |
|  |  | Daucosterol^30^ | 5742590 |
|  |  | Methyl eucomate^30^ | 139074856 |
|  |  | Eucomic acid^30^ | 23757219 |
|  |  | Phytol^30^ | 5280435 |
|  |  | Himachalene^30^ | 11586487 |
|  |  | Spathulenol^30^ | 92231 |
|  |  | Aromadendrene^30^ | 91354 |
|  |  | Caryophyllene^30^ | 5281515 |
| 11 | *Oroxylum indicum* (Linn.) Kurz | Baicalein^31^ | 5281605 |
|  |  | Biochanin A^31^ | 5280373 |
|  |  | Chrysin^31^ | 5281607 |
|  |  | Ellagic acid^31^ | 5281855 |
|  |  | Oroxylin A^31^ | 5320315 |
|  |  | β-Sitosterol^31^ | 222284 |
|  |  | Ursolic acid^31^ | 64945 |
|  |  | Aequinetin^31^ | 15558425 |
|  |  | Arabinopyranoside^31^ | 439195 |
|  |  | Pinocembrin^31^ | 68071 |
|  |  | Pinobanksin^31^ | 73202 |
|  |  | Lupeol^31^ | 259846 |
|  |  | Echinulin^31^ | 115252 |
|  |  | Dimethyl Sulfone^31^ | 6213 |
|  |  | Baicalein 7-O-glucoside^32^ | 5320313 |
|  |  | Baicalein 7-O-glucuronide^32^ | 64982 |
|  |  | Uracil^32^ | 1174 |
|  |  | Dihydropinosylvin^32^ | 442700 |
|  |  | Pinosylvin^32^ | 5280457 |
|  |  | Chrysin-7-O-glucuronide^32^ | 14135335 |
|  |  | Baicalein-6-O-glucoside^32^ | 5321896 |
|  |  | Baicalein-3-O-glucoside^32^ | 101876630 |
|  |  | Oroxin A^32^ | 5320313 |
|  |  | Scutellarein^33^ | 5281697 |
|  |  | Scutellarein 7-O-glucuronide^33^ | 554001 |
|  |  | Aloe-emodin^33^ | 10207 |
|  |  | Salidroside^33^ | 159278 |
|  |  | 2(3,4-dihydroxyphenyl)-ethyl glucoside^33^ | 5316821 |
|  |  | Acteoside^33^ | 5281800 |
|  |  | Rengyol^33^ | 363707 |
|  |  | Rengyoxide^33^ | 14353410 |
|  |  | Cornoside^33^ | 11809239 |
|  |  | Norwogonin^33^ | 5281674 |
|  |  | Acacetin^33^ | 5280442 |
|  |  | Hispidulin^33^ | 5281628 |
|  |  | Isorhamnetin^33^ | 5281654 |
|  |  | Kaempferol 7-O-β-D glucopyranoside^33^ | 5480982 |
|  |  | Isoquercetin^33^ | 5280804 |
|  |  | Apigenin^33^ | 5280443 |
|  |  | Kaempferol^33^ | 5280863 |
|  |  | Quercetin^33^ | 5280343 |
|  |  | Cholest-5-ene-3,7-diol^33^ | 22826216 |
|  |  | β-sitosterol glucoside^33^ | 5742590 |
|  |  | Caprylic acid^33^ | 379 |
|  |  | Mauric acid^33^ | 3893 |
|  |  | Myristic acid^33^ | 11005 |
|  |  | Pyristoleic acid^33^ | 5281119 |
|  |  | Palmitoleic acid^33^ | 445638 |
|  |  | Linoleic acid^33^ | 5280450 |
|  |  | Palmitic acid^33^ | 985 |
|  |  | Stearic acid^33^ | 5281 |
|  |  | Oleic acid^33^ | 445639 |
|  |  | 2-Methyl-6-phenyl-4H-pyran-4-one^33^ | 120521 |
|  |  | Adenosine^33^ | 60961 |
|  |  | Zarzissine^33^ | 6400641 |
|  |  | 6-hydroxyluteolin^33^ | 5281642 |
|  |  | 6-methoxyluteolin^33^ | 5317284 |
|  |  | 7-O-Methylchrysin^33^ | 5281954 |
|  |  | Dihydrooroxylin A^33^ | 5316733 |
|  |  | Pectolinarigenin^33^ | 5320438 |
|  |  | Lapachol^33^ | 3884 |
|  |  | p-Coumaric acid^33^ | 637542 |
|  |  | Prunetin^33^ | 5281804 |
|  |  | 2-acetylnaphtho [2,3-b]-furan-4, 9-dione^33^ | 10331844 |
|  |  | Catalponol^33^ | 169570 |
|  |  | Stigmasterol glucoside^33^ | 6602508 |
|  |  | Pinostrobin^34^ | 73201 |
|  |  | Stigmast-7-en-3-ol^34^ | 12315376 |
| 12 | *Oxalis corniculata* Linn*.* | β-sitosterol^35^ | 222284 |
|  |  | Betulin^35^ | 72326 |
|  |  | 4-hydroxybenzoic acid^35^ | 135 |
|  |  | Ethyl gallate^35^ | 13250 |
|  |  | Apigenin^35^ | 5280443 |
|  |  | Palmitic acid^35^ | 985 |
|  |  | Oleic acid^35^ | 445639 |
|  |  | Linoleic acid^35^ | 5280450 |
|  |  | Linolenic acid^35^ | 5280934 |
|  |  | Stearic acid^35^ | 5281 |
|  |  | Tartaric acid^35^ | 875 |
|  |  | Citric acid^35^ | 311 |
|  |  | Calcium oxalate^35^ | 33005 |
|  |  | Acacetin^35^ | 5280442 |
|  |  | Vanillic acid^35^ | 8468 |
|  |  | Syringic acid^35^ | 10742 |
|  |  | Isoorientin^35^ | 114776 |
|  |  | Isovitexin^35^ | 162350 |
|  |  | 5-hydroxy-7,8-dimethoxyflavone^35^ | 188316 |
|  |  | 5-hydroxy-3', 4', 6, 7, 8-pentamethoxyflavone^35^ | 11079623 |
|  |  | 5-hydroxy-3, 6, 7, 4′-tetramethoxyflavone^35^ | 5318355 |
|  |  | Luteolin^36^ | 5280445 |
|  |  | Luteolin-7-O-β-D-glucoside^36^ | 5280637 |
| 13 | *Paederia foetida* Linn. | β-sitosterol^37^ | 222284 |
|  |  | Paederoside^37^ | 442432 |
|  |  | Asperuloside^37^ | 84298 |
|  |  | Carotene^37^ | 6419725 |
|  |  | vitamin C^37^ | 54670067 |
|  |  | Lupeol^37^ | 259846 |
|  |  | Scandoside^37^ | 21602023 |
|  |  | Friedelin^37^ | 91472 |
|  |  | Campesterol^37^ | 173183 |
|  |  | Ursolic acid^37^ | 64945 |
|  |  | Hentriacontane^37^ | 12410 |
|  |  | Hentriacontanol^37^ | 68345 |
|  |  | Ceryl alcohol^37^ | 68171 |
|  |  | Palmitic acid^37^ | 985 |
|  |  | Methyl mercaptan^37^ | 878 |
|  |  | Ellagic acids^37^ | 5281855 |
|  |  | Epifriedelinol^37^ | 119242 |
|  |  | Stigmasterol^37^ | 5280794 |
|  |  | Butanedione^38^ | 650 |
|  |  | Pentan-2-one^38^ | 7895 |
|  |  | Pentanal^38^ | 8063 |
|  |  | 2-Methylbut-3-en-2-ol^38^ | 8257 |
|  |  | s-Methyl thioacetate^38^ | 73750 |
|  |  | Dimethyl disulfide^38^ | 12232 |
|  |  | Hexanal^38^ | 6184 |
|  |  | β-Pinene^38^ | 14896 |
|  |  | (E)-Pent-3-en-2-one^38^ | 637920 |
|  |  | Butan-1-ol^38^ | 263 |
|  |  | Pent-1-en-3-one^38^ | 15394 |
|  |  | Pent-3-en-2-ol^38^ | 15289 |
|  |  | Pyridine^38^ | 1049 |
|  |  | β-Phellandrene^38^ | 11142 |
|  |  | 3-Methylbutan-1-ol^38^ | 31260 |
|  |  | Pentan-1-ol^38^ | 6276 |
|  |  | 3-Methylbut-2-en-1-ol^38^ | 11173 |
|  |  | Hexanol^38^ | 8103 |
|  |  | (E)-Hex-3-en-1-ol^38^ | 5284503 |
|  |  | Dimethyl trisulfide^38^ | 19310 |
|  |  | (Z)-Hex-3-en-1-ol^38^ | 5281167 |
|  |  | 3-(Methythio)propanal^38^ | 18635 |
|  |  | s.s-Dimethyl dithiocarbonate^38^ | 313474 |
|  |  | 2-Furancarboxaldehyde^38^ | 7362 |
|  |  | Linalool oxide^38^ | 102611 |
|  |  | Benzaldehyde^38^ | 240 |
|  |  | Linalool^38^ | 6549 |
|  |  | Phenylacetaldehyde^38^ | 998 |
|  |  | 2-Furanmethanol^38^ | 7361 |
|  |  | Benzofuran^38^ | 9223 |
|  |  | α-Terpineol^38^ | 17100 |
|  |  | 1,2-Dimethoxybenzene^38^ | 7043 |
|  |  | Methyl salicylate^38^ | 4133 |
|  |  | Nerol^38^ | 643820 |
|  |  | Geraniol^38^ | 637566 |
|  |  | 2-Methoxyphenol^38^ | 460 |
|  |  | 2-Phenylethanol^38^ | 6054 |
|  |  | Eugenol^38^ | 3314 |
|  |  | 2,3-Dihydrobenzofuran^38^ | 10329 |
| 14 | *Paeonia suffruticosa* Andr. | Quinic acid^39^ | 6508 |
|  |  | Galloylquinic acid^39^ | 129650210 |
|  |  | Gallic acid^39^ | 370 |
|  |  | Kaempferol 3,7-di-O-glucoside^39^ | 6325460 |
|  |  | Isorhamnetin 3,7-di-O-glucoside^39^ | 5323537 |
|  |  | Paeoniflorin^39^ | 442534 |
|  |  | Benzoyloxypaeoniflorin^39^ | 21631107 |
|  |  | Pentagalloylglucose^39^ | 65238 |
|  |  | Hexagalloylglucose^39^ | 129630523 |
|  |  | Sucrose^40^ | 5988 |
|  |  | Mudanoside B^40^ | 21604164 |
|  |  | Oxypaeoniflorin^40^ | 21631105 |
|  |  | Catechin^40^ | 9064 |
|  |  | Paeonolide^40^ | 442923 |
|  |  | Trigalloylglucose^40^ | 90116889 |
|  |  | Methyl gallate^40^ | 7428 |
|  |  | Albiflorin^40^ | 24868421 |
|  |  | Suffruticoside A^40^ | 9986231 |
|  |  | Tetragalloylglucose^40^ | 13888120 |
|  |  | Suffruticoside B^40^ | 10258205 |
|  |  | Galloylpaeoniflorin^40^ | 46882879 |
|  |  | Mudanpioside H^40^ | 71457654 |
|  |  | Mudanpioside C^40^ | 21631098 |
|  |  | Mudanpioside J^40^ | 21593828 |
|  |  | Octagalloylglucose^40^ | 54085664 |
|  |  | Nonagalloylglucose^40^ | 53831489 |
|  |  | Mudanpioside B^40^ | 21631102 |
|  |  | Luteolin^40^ | 5280445 |
|  |  | Quercetin^40^ | 5280343 |
|  |  | Benzoylpaeoniflorin^40^ | 21631106 |
|  |  | Apigenin^40^ | 5280443 |
|  |  | Paeonilactone B^40^ | 10375422 |
|  |  | Paeonol^40^ | 11092 |
|  |  | Paeonenoide A^40^ | 12134773 |
|  |  | Hederagenin^40^ | 73299 |
|  |  | 2-Phenylethanol^41^ | 6054 |
|  |  | 1,3,5-trimethoxybenzene^41^ | 69301 |
|  |  | β-citronellol^41^ | 8842 |
|  |  | 6,9-heptadecadiene^41^ | 5365698 |
|  |  | 2-heptanol ^41^ | 10976 |
|  |  | Pentadecane^41^ | 12391 |
|  |  | (Z)-3-nonen-1-ol^41^ | 5364631 |
|  |  | Geraniol^41^ | 637566 |
| 15 | *Panax pseudo-ginseng* Wall. | Panaxynol^42^ | 5281149 |
|  |  | Chikusetsusaponin IV  ^42^ | 10079497 |
|  |  | Ginsenoside-Rd^42^ | 24721561 |
|  |  | Ginsenoside rb1^42^ | 9898279 |
|  |  | Ginsenoside Re^42^ | 441921 |
|  |  | Ginsenoside RG1^42^ | 441923 |
|  |  | Gypenoside XVII^42^ | 44584555 |
|  |  | Notoginsenoside-R1^42^ | 441934 |
|  |  | Majonoside-R2^42^ | 24838365 |
|  |  | Pseudo-ginsenoside-F11^42^ | 134688664 |
|  |  | Ginsenoside-Rb3^42^ | 12912363 |
|  |  | Ginsenoside-Rg2^42^ | 6441009 |
|  |  | Notoginsenoside-R2^42^ | 21599925 |
|  |  | 24(S)-Pseudoginsenoside-F11^42^ | 44144329 |
|  |  | Chikusetsusaponin-IVa^42^ | 13909684 |
|  |  | Chikusetsusaponin-V^42^ | 11815492 |
|  |  | 20-S-protopanaxadiol^43^ | 11213350 |
|  |  | Dammarane^43^ | 9548714 |
|  |  | Triterpenoid^43^ | 451674 |
|  |  | Oleanolic acid^43^ | 10494 |
|  |  | Panacene^43^ | 181799 |
| 16 | *Papaver somniferum* Linn. | Morphine^44^ | 5288826 |
|  |  | Codein^44^ | 5284371 |
|  |  | Neopine^44^ | 5462437 |
|  |  | Thebain^44^ | 5324289 |
|  |  | Porphyroxine^44^ | 601829 |
|  |  | Hydrocotarnine^44^ | 3646 |
|  |  | Narcotoline^44^ | 442330 |
|  |  | dl-narcotine^44^ | 4544 |
|  |  | Oxynarcotine^44^ | 5320347 |
|  |  | Narceine^44^ | 8564 |
|  |  | Papaverine^44^ | 4680 |
|  |  | Xanthaline^44^ | 96932 |
|  |  | dl-Laudanine^44^ | 92732 |
|  |  | Codamine^44^ | 20056510 |
|  |  | Laudanosine^44^ | 15548 |
|  |  | Protopine^44^ | 4970 |
|  |  | Cryptopine^44^ | 72616 |
|  |  | Aporeine^44^ | 800239 |
|  |  | Rhoeadine^44^ | 197775 |
|  |  | Meconic acid^44^ | 10347 |
|  |  | Lactic acid^44^ | 612 |
|  |  | Malic acid^44^ | 525 |
|  |  | Tartaric acid^44^ | 875 |
|  |  | Citric acid^44^ | 311 |
|  |  | Acetic acid^44^ | 176 |
|  |  | Succinic acid^44^ | 1110 |
|  |  | Oripavine^45^ | 5462306 |
|  |  | Sinomenine^45^ | 5459308 |
|  |  | Hasubanonine^45^ | 442246 |
|  |  | 6-Acetylmorphine^45^ | 5462507 |
|  |  | Apomorphine^45^ | 6005 |
|  |  | Hydromorphone^45^ | 5284570 |
|  |  | Bismorphine A^46^ | 101016063 |
|  |  | Bismorphine B^46^ | 10907908 |
|  |  | Noscapine^47^ | 275196 |
| 17 | *Paris polyphylla* Smith | 2H-Pyran-2-One, Tetrahydro-6-Methyl^48^ | 642771 |
|  |  | 6-Oxa-bicyclo[3.1.0]Hexan-3-one^48^ | 535532 |
|  |  | Pentanal^48^ | 8063 |
|  |  | Guanosine^48^ | 135398635 |
|  |  | 1,4: 3,6- Dianhydro-.alpha.-d-glucopyranose^48^ | 549469 |
|  |  | 5-Hydroxymethylfurfural^48^ | 237332 |
|  |  | 1,6- Octadien-3-ol, 3,7-Dimethyl-,2-Aminobenzoate^48^ | 23535 |
|  |  | 2-Methoxy-4-Vinylphenol^48^ | 332 |
|  |  | Decanoic Acid Ethyl Ester^48^ | 8048 |
|  |  | 1,2,3-Trimethoxybenzene^48^ | 12462 |
|  |  | Hexadecanoic acid Ethyl Ester^48^ | 12366 |
|  |  | Hexadecanoic Acid^48^ | 985 |
|  |  | 11,14-Eicosadienoic acid, Methyl Ester^48^ | 5365566 |
|  |  | Ethyl(9Z, 12Z)-9,12-Octadecadienoate^48^ | 5282184 |
|  |  | Linolenin, 1- mono^48^ | 5367328 |
|  |  | Linoleic acid^48^ | 5280450 |
|  |  | 15-Hydroxypentadecanoic acid^48^ | 78360 |
|  |  | 3-cyclopentylpropionic acid, 2-dimethylaminoethyl ester^48^ | 91693811 |
|  |  | 1,E-6, Z-11-Hexadecatriene^48^ | 5365572 |
|  |  | 17-Hydroxy-4,4-Dimethyl estran-3-One^48^ | 21634211 |
|  |  | Palmitin,2-mono^48^ | 123409 |
|  |  | Ethyl (9Z, 12Z)-9,12-Octadecadienoate^48^ | 5282184 |
|  |  | Linolein, 2- mono^48^ | 5365676 |
|  |  | Octadecanoic , 2,3-dihydroxypropyl ester^48^ | 44147726 |
|  |  | E-11(12 Cyclopropyl) dodecen-1-ol acetate^48^ | 5363510 |
|  |  | Squalene^48^ | 638072 |
|  |  | Spirost-5-EN-3-OL^48^ | 234096 |
|  |  | Diosgenin Acetate^48^ | 101952 |
|  |  | Vitamin E^48^ | 14985 |
|  |  | Spirost-5-EN-3-OL, (3.beta., 25R)^48^ | 6432492 |
|  |  | gamma-Sitosterol^48^ | 457801 |
|  |  | 7.beta.-hydroxydiosgenin^48^ | 587595 |
|  |  | Diosgenin^49^ | 99474 |
|  |  | Polyphyllin VI^49^ | 10417550 |
|  |  | Polyphyllin VII^49^ | 71307572 |
|  |  | Polyphyllin C^49^ | 44429637 |
|  |  | Stigmasterol^49^ | 5280794 |
|  |  | Stigmasterol-3-O- β -D-glucoside^49^ | 6602508 |
|  |  | Pennogenin^49^ | 12314056 |
|  |  | 24-αhydroxy pennogenin^49^ | 101666899 |
|  |  | Polyphyllin A^49^ | 11827970 |
|  |  | Polyphyllin B^49^ | 328441 |
|  |  | Polyphyllin E^49^ | 102594501 |
|  |  | Polyphyllin F^49^ | 71664512 |
|  |  | Polyphyllin G^49^ | 46200822 |
|  |  | Polyphyllin H^49^ | 101615586 |
|  |  | Padelaoside B^50^ | 44226432 |
|  |  | Gracillin^50^ | 159861 |
| 18 | *Peperomia blanda* (Jacq.) Kunth | 4H-pyran-4-one^51^ | 7968 |
|  |  | n-Hexadecanoic acid^51^ | 985 |
|  |  | n-Nonadecanol-1^51^ | 80281 |
|  |  | Caryophyllene^51^ | 5281515 |
|  |  | Tetrapentacontane^51^ | 521846 |
|  |  | (Phenylthio)acetic acid, hexadecyl ester^51^ | 531636 |
|  |  | Phen-1,3-diol,2- dodecanoyl^51^ | 365016 |
|  |  | (Phenylthio)acetic acid, octadecyl ester^51^ | 531637 |
|  |  | Vitamin E^51^ | 14985 |
|  |  | Gamma-sitosterol^51^ | 457801 |
|  |  | caryophyllene oxide^51^ | 1742210 |
|  |  | 1-Hexadecanol, 2- methyl^51^ | 17218 |
|  |  | Phytol^51^ | 5280435 |
|  |  | cis-10-Heptadecenoic acid^51^ | 5312435 |
|  |  | Hexacontane^51^ | 24318 |
|  |  | β-Sitosterol^51^ | 222284 |
|  |  | Stigmasterol^51^ | 5280794 |
|  |  | Blandachromene I^52^ | 101396971 |
|  |  | Blandachromene II^52^ | 101396972 |
|  |  | Vicenin-2^53^ | 442664 |
| 19 | *Perilla frutescens* var. *purpurascens* (Hayata) H.W. Li | Myristic acid^54^ | 11005 |
|  |  | Palmitic acid^54^ | 985 |
|  |  | Stearic acid^54^ | 5281 |
|  |  | Oleic acid^54^ | 445639 |
|  |  | Linoleic acid^54^ | 5280450 |
|  |  | α-linolenic acid^54^ | 5280934 |
|  |  | Cis-11-eicosenoic acid^54^ | 5282768 |
|  |  | Luteolin-7-O-glucoside^55^ | 5280637 |
|  |  | Apigenin-7-O-glucoside^55^ | 5280704 |
|  |  | Luteolin^55^ | 5280445 |
|  |  | Apigenin^55^ | 5280443 |
|  |  | Chrysoeriol^55^ | 5280666 |
|  |  | Rosmarinic acid^55^ | 5281792 |
|  |  | Rosmarinic acid methyl ester^55^ | 3012090 |
|  |  | Caffeic acid^55^ | 689043 |
| 20 | *Periploca calophylla* (Woght) Falc. | Periplocin^56^ | 14463159 |
|  |  | Periplogenin^56^ | 10574 |
|  |  | Periplocoside M^56^ | 134715182 |
|  |  | Locin^56^ | 590475 |
|  |  | Calocinin^56^ | 189455 |
|  |  | β-sitosterol^56^ | 222284 |
|  |  | Cymarose^56^ | 73425443 |
|  |  | Perisaccharide B^56^ | 25149487 |
|  |  | β-amyrin^56^ | 73145 |
|  |  | Asiatic acid^56^ | 119034 |
|  |  | α-amyrin acetate^56^ | 293754 |
|  |  | Lupeol^56^ | 259846 |
|  |  | Sinapic acid^56^ | 637775 |
|  |  | Cleomiscosin A^56^ | 442510 |
|  |  | Physcion^56^ | 10639 |
|  |  | Vanillic acid^56^ | 8468 |
|  |  | Erigeside C^56^ | 14132346 |
|  |  | 4-hydroxy-3,5-dimethoxy benzaldehyde^56^ | 8655 |
|  |  | 1-triacontanol^56^ | 68972 |
| 21 | *Phyllanthus emblica* Linn. | Ascorbic acid^57^ | 54670067 |
|  |  | Linolenic acid^57^ | 5280934 |
|  |  | Linoleic acid^57^ | 5280450 |
|  |  | Oleic acid^57^ | 445639 |
|  |  | Stearic acid^57^ | 5281 |
|  |  | Palmitic acid^57^ | 985 |
|  |  | Myristic acid^57^ | 11005 |
|  |  | D-myo-inositol^57^ | 892 |
|  |  | D-galacturonic acid^57^ | 439215 |
|  |  | Emblicanin A^57^ | 119058016 |
|  |  | Emblicanin B^57^ | 119058017 |
|  |  | Pedunculagin^57^ | 442688 |
|  |  | Punigluconin^57^ | 21637585 |
|  |  | Gallic acid^57^ | 370 |
|  |  | Amlaic acid^57^ | 131751968 |
|  |  | β-carotene^57^ | 5280489 |
|  |  | β-sitosterol^57^ | 222284 |
|  |  | Chebulagic acid^57^ | 442674 |
|  |  | Chebulic acid^57^ | 71308174 |
|  |  | Chebulinic acid^57^ | 72284 |
|  |  | Corilagin^57^ | 73568 |
|  |  | Ellagic acid^57^ | 5281855 |
|  |  | Gibberellin^57^ | 6466 |
|  |  | Kaempferol^57^ | 5280863 |
|  |  | Leucodelphinidin^57^ | 3081374 |
|  |  | Phyllantidine^57^ | 12314211 |
|  |  | Quercetin^57^ | 5280343 |
|  |  | Riboflavin^57^ | 493570 |
|  |  | Rutin^57^ | 5280805 |
|  |  | Zeatin^57^ | 449093 |
|  |  | 2-Furanmethanol^58^ | 7361 |
|  |  | Trans-caryophyllene^58^ | 5281515 |
|  |  | Cyclohexane^58^ | 8078 |
|  |  | Caryophyllene^58^ | 5281515 |
|  |  | Sativen^58^ | 530427 |
|  |  | Delta-guaiene^58^ | 94275 |
|  |  | Tetradecanoic acid^58^ | 11005 |
|  |  | Octadecanal^58^ | 12533 |
|  |  | 3-Eicosyne^58^ | 549159 |
|  |  | Octadecanoic acid^58^ | 5281 |
|  |  | 9,12-Octadecadienoic acid^58^ | 5280450 |
|  |  | 9-Hexadecenoic acid^58^ | 5282745 |
|  |  | 1-Hexadecanol^58^ | 2682 |
|  |  | 11-Tetradecen-1-ol^58^ | 36657 |
|  |  | 3,7,11-Tridecatrienenitrile^58^ | 101282417 |
|  |  | 2H-Pyran^58^ | 186148 |
|  |  | Lignan^59^ | 261166 |
|  |  | Teraniin^59^ | 3001497 |
|  |  | Tercatain^59^ | 14411426 |
|  |  | 1,6-di-O-galloyl-β-D-glucose^59^ | 91227631 |
|  |  | Digallic acid^59^ | 341 |
| 22 | *Phytolacca americana* Linn. | Oxyresveratrol^60^ | 5281717 |
|  |  | Gnetol^60^ | 45382232 |
|  |  | Methanol-hydrochloric acid^61^ | 21893726 |
|  |  | Dioxane-hydrochloric acid^61^ | 20443148 |
|  |  | Phytolaccoside A^61^ | 102469813 |
|  |  | Phytolaccoside B^61^ | 441939 |
|  |  | Phytolaccoside D^61^ | 73157052 |
|  |  | Phytolaccoside D2^61^ | 13878346 |
|  |  | Phytolaccoside E^61^ | 125210 |
|  |  | Phytolaccoside G^61^ | 128258 |
|  |  | Phytolaccasaponin B^61^ | 173768 |
| 23 | *Pimpinella candolleana* Wight et Arn. | Ursolic acid^62^ | 64945 |
|  |  | Luteolin^62^ | 5280445 |
|  |  | Urea^62^ | 1176 |
|  |  | Stigmasta-5,22-dien-3-ol acetate^62^ | 129885398 |
|  |  | Erythrol^62^ | 222285 |
|  |  | Isovitexin^62^ | 162350 |
|  |  | 1-(4-hydroxyphenyl)-1,2- ethanediol^62^ | 76764608 |
|  |  | Daucosterol^62^ | 5742590 |
|  |  | B-sitosterol^62^ | 222284 |
| 24 | *Pinellia ternata* (Thunb.) Makino | Isolariciresinol^63^ | 160521 |
|  |  | Pinoresinol 4-O-beta-D-glucopyranoside^63^ | 486614 |
|  |  | 1-(4-hydroxy-3-methoxyphenyl)-2-{4-[(E)-3-hydroxy-1-propenyl]-2-methoxyphenoxy}-1,3-propanediol^63^ | 193323 |
|  |  | Dehydrodiconiferyl alcohol 4-O-beta-D-glucopyranoside^63^ | 5316442 |
|  |  | Neoolivil^63^ | 9976812 |
|  |  | Medioresinol^63^ | 181681 |
|  |  | Americanol A^63^ | 637304 |
|  |  | Pinoresinol^63^ | 73399 |
|  |  | Burselignan^63^ | 11631864 |
|  |  | E-p-coumaryl alcohol^64^ | 5280535 |
|  |  | Coniferin^64^ | 5280372 |
|  |  | Inosine^64^ | 135398641 |
|  |  | Cytidine^64^ | 6175 |
|  |  | Pedatisectine B^64^ | 190 |
|  |  | Protocatechuic aldehyde^64^ | 13886890 |
|  |  | Erythritol^64^ | 222285 |
|  |  | Dibutyl phthalate^64^ | 3026 |
|  |  | Chrysophanol^64^ | 10208 |
|  |  | Pinellic acid^64^ | 9858729 |
|  |  | Gingerol^64^ | 3473 |
|  |  | 1,3-dioleoylglycerol^65^ | 5497165 |
|  |  | 1,3-dilinoleoylglycerol^65^ | 45934042 |
|  |  | Alpha-Tocospiro A^65^ | 21674156 |
|  |  | Squalene^65^ | 638072 |
| 25 | *Pinus kesiya Royle* ex Gordon | Dehydroabietic acid^66^ | 94391 |
|  |  | Cedrusin^66^ | 11210164 |
|  |  | Zeorin^67^ | 159931 |
|  |  | Atranorin^67^ | 68066 |
|  |  | Isopimaric acid^68^ | 442048 |
|  |  | Pimarol^68^ | 12314285 |
|  |  | Isopimarol^68^ | 15586712 |
|  |  | 15-hydroxyabietic acid^68^ | 643004 |
|  |  | alpha-Pinene^68^ | 6654 |
|  |  | β-pinene^68^ | 14896 |
| 26 | *Plantago minuta* Pall. | Shanzhiside^69^ | 11948668 |
|  |  | Geniposidic acid^69^ | 443354 |
|  |  | Alpinoside^69^ | 46882792 |
|  |  | Plantamajoside^69^ | 5281788 |
|  |  | Plantagoguanidinic acid^69^ | 132560907 |
|  |  | Acetoside^69^ | 5281800 |
|  |  | Methyl hesperidin^69^ | 46783847 |
| 27 | *Piper boehmeriaefolium*  (Miq.) C. DC. | alpha-Pinene^70^ | 6654 |
|  |  | Camphene^70^ | 6616 |
|  |  | Verbenene^70^ | 6427476 |
|  |  | Sabinene^70^ | 18818 |
|  |  | Beta-Pinene^70^ | 14896 |
|  |  | Myrcene^70^ | 31253 |
|  |  | Alpha-Phellandrene^70^ | 7460 |
|  |  | Alpha-Terpinene^70^ | 7462 |
|  |  | p-Cymene^70^ | 7463 |
|  |  | Limonene^70^ | 22311 |
|  |  | 1,8-Cineol^70^ | 2758 |
|  |  | (E)-beta-ocimene^70^ | 5281553 |
|  |  | Gamma-Terpinene^70^ | 7461 |
|  |  | alpha-Terpinolene^70^ | 11463 |
|  |  | Linalool^70^ | 6549 |
|  |  | trans-Pinocarveol^70^ | 88302 |
|  |  | trans-Verbenol^70^ | 89664 |
|  |  | Borneol^70^ | 64685 |
|  |  | Terpinen-4-ol^70^ | 11230 |
|  |  | alpha-Thujenal^70^ | 129847841 |
|  |  | p-Cymen-8-ol^70^ | 14529 |
|  |  | Alpha-Terpineol^70^ | 17100 |
|  |  | Verbenone^70^ | 29025 |
|  |  | trans-Carveol^70^ | 94221 |
|  |  | (E)- Citral^70^ | 638011 |
|  |  | Borneol acetate^70^ | 6448 |
|  |  | Eugenol^70^ | 3314 |
|  |  | Isoledene^70^ | 530426 |
|  |  | alpha-Copaene^70^ | 19725 |
|  |  | Methyl eugenol^70^ | 7127 |
|  |  | beta-Caryophyllene^70^ | 5281515 |
|  |  | alpha-Guaiene^70^ | 5317844 |
|  |  | gamma-Gurjunene^70^ | 90805 |
|  |  | alpha-Amorphene^70^ | 12306052 |
|  |  | Epizonarene^70^ | 595385 |
|  |  | gamma-Cadinene^70^ | 92313 |
|  |  | delta-Cadinene^70^ | 441005 |
|  |  | cis-Calamenene^70^ | 6429077 |
|  |  | Spathulenol^70^ | 92231 |
|  |  | Caryophyllene oxide^70^ | 1742210 |
|  |  | Widdrol^70^ | 94334 |
|  |  | beta-Oplopenone^70^ | 14038847 |
|  |  | allo-Aromadendrene epoxide^70^ | 91746712 |
|  |  | alpha-Cadinol^70^ | 10398656 |
|  |  | Germacrene epoxide^70^ | 91747492 |
|  |  | (Z)-9 octadecanamide^70^ | 5283387 |
|  |  | (Z)-13-Docosenamide^70^ | 5365371 |
|  |  | 14-Nor-cadin-5-en-4-one isomer A^70^ | 91750041 |
|  |  | (þ)-sesamin^71^ | 72307 |
|  |  | (þ)-5- methoxysesamin^71^ | 179572 |
|  |  | 3-(4-hydroxy-3,5- dimethoxyphenyl)propanoylpyrrole^71^ | 50994066 |
|  |  | 3- (3,4,5-trimethoxyphenyl)propanoylpyrrole^71^ | 50994067 |
|  |  | Pellitorine^71^ | 5318516 |
|  |  | N-isobutyl2E,4E-dodecadienamide^71^ | 6443006 |
|  |  | N-Isobutyl-2E,4Eoctadecadienamide^71^ | 9974234 |
|  |  | Pipercallosidine^71^ | 5372065 |
|  |  | Retrofractamide C^71^ | 25255091 |
|  |  | Pipercide^71^ | 5372162 |
|  |  | Guineensine^71^ | 6442405 |
|  |  | Brachystamide-B^71^ | 10047263 |
|  |  | Sarmentine^71^ | 6440616 |
|  |  | Piperamide-C9:2 (2E,8E)^71^ | 131752411 |
|  |  | Piperolein-B^71^ | 21580213 |
|  |  | Piperchabamide B^71^ | 44453655 |
|  |  | (þ)-cassipourol^71^ | 11500301 |
|  |  | (-)-loliolide^71^ | 100332 |
|  |  | Blumenol A^71^ | 137705650 |
|  |  | Pipernonaline^72^ | 9974595 |
|  |  | 1-[(9E)-10-(3,4-methylenedioxyphenyl)-9-decenoyl]pyrrolidine^72^ | 16041826 |
|  |  | Eupomatenoid-6^72^ | 6261723 |
|  |  | Conocarpan^72^ | 6474521 |
|  |  | (2E, 4E)-N-[4-hydroxy-3-methoxyphenyl)ethyl]-2,4-decadienamide^73^ | 50994065 |
|  |  | (2E,4E)-N-[2-(Methylsulfinyl)ethyl]-2,4-decadienamide^73^ | 50994064 |
|  |  | 1-[(2E,4E,6E)-2,4,6-Dodecatrienoyl]pyrrolidine^73^ | 50994143 |
|  |  | 1-[(2E,4Z,8E)-9-(3,4-Methylenedioxyphenyl)-2,4,8-nonatrienoyl]pyrrolidine^73^ | 50994144 |

REFERENCES

1. Chen, Y., Yang, Q. & Zhang, Y. Lycopodium japonicum: A comprehensive review on its phytochemicals and biological activities. *Arab. J. Chem.* **13**, 5438–5450 (2020).

2. He, J. *et al.* Lycopodine-Type Alkaloids from Lycopodium japonicum. *Nat. Products Bioprospect.* **4**, 213–219 (2014).

3. Xia, Z. H. *et al.* Five new pregnane glycosides from the stems of Marsdenia tenacissima. *J. Asian Nat. Prod. Res.* **13**, 477–485 (2011).

4. Wang, P., Yang, J., Zhu, Z. & Zhang, X. Marsdenia tenacissima: A Review of Traditional Uses, Phytochemistry and Pharmacology. *Am. J. Chin. Med.* **46**, 1449–1480 (2018).

5. Sanaye, M. M., Joglekar, C. S. & Pagare, N. P. Mimosa - A brief overview. *J. Pharmacogn. Phytochem.* **4**, 182–187 (2015).

6. TK, V., J, D., R, S. & S, R. Composition of the Essential Oil From Mimosa Pudica Linn. *Asian J. Pharm. Clin. Res.* **12**, 170–172 (2019).

7. K., J., G., N. & C., K. Mimosa Pudica Linn- A shyness princess: A review of its plant movement, active constituents, uses and pharmacological activity. *Int. J. Pharm. Sci. Res.* **5**, 5104–5118 (2014).

8. Muhammad, G., Hussain, M. A., Jantan, I. & Bukhari, S. N. A. Mimosa pudica L., a High-Value Medicinal Plant as a Source of Bioactives for Pharmaceuticals. *Compr. Rev. Food Sci. Food Saf.* **15**, 303–315 (2016).

9. Rozina, R. & Rozina, R. Pharmacological and biological activities of Mirabilis jalapa L . Corresponding author *. *Int. J. Pharmacol. Res.* **6**, 160–168 (2016).

10. Linn, J., Shaik, S., Rajendra, Y. & Chandra, P. J. Available through Online Review Article PHYTOCHEMICAL AND PHARMACLOGICAL STUDIES OF MIRABILIS. **4**, 2075–2084 (2012).

11. Rodrigues, E. L. *et al.* Nutraceutical and medicinal potential of the Morus species in metabolic dysfunctions. *Int. J. Mol. Sci.* **20**, (2019).

12. Yuan, Q. & Zhao, L. The Mulberry (Morus alba L.) Fruit - A Review of Characteristic Components and Health Benefits. *J. Agric. Food Chem.* **65**, 10383–10394 (2017).

13. Miyaichi, Y., Ohichi, M., Yaguchi, K., Kawata, Y. & Kizu, H. Studies on the constituents of the leaves of Conandron ramondioides. *J. Nat. Med.* **60**, 159–160 (2006).

14. Yang, Y. *et al.* Chemical constituents of Morus alba L. and their inhibitory effect on 3T3-L1 preadipocyte proliferation and differentiation. *Fitoterapia* **98**, 222–227 (2014).

15. Hapuarachchi, N.S; Hapurarachchi, S. D. & Senerath, W. T. P. S. . Identification of the chemical identities in calli of different explants from Munronia pinnata (Wall.)Theob. *Int. J. Sci. Eng. Res.* **8**, 527–533 (2017).

16. Silva, B. J. C., Seca, A. M. L., Barreto, M. do C. & Pinto, D. C. G. A. Recent breakthroughs in the antioxidant and anti-inflammatory effects of Morella and Myrica species. *Int. J. Mol. Sci.* **16**, 17160–17180 (2015).

17. Sun, C., Huang, H., Xu, C., Li, X. & Chen, K. Biological Activities of Extracts from Chinese Bayberry (Myrica rubra Sieb. et Zucc.): A Review. *Plant Foods Hum. Nutr.* **68**, 97–106 (2013).

18. Zhang, X. *et al.* Phytochemical characterization of Chinese bayberry (Myrica rubra Sieb. et Zucc.) of 17 cultivars and their antioxidant properties. *Int. J. Mol. Sci.* **16**, 12467–12481 (2015).

19. Li, Jing; Wang, Huiling; Li, Jian; Liu, Yonggang; Ding, H. LC-MS analysis of Myrica rubra extract and its hypotensive e ff ects via the inhibition of GLUT 1 and activation of the NO / Akt / eNOS signaling pathway. *R. Soc. Chem.* **10**, 5371–5384 (2020).

20. Fang, Zhongxiang; Zhang, Min; Tao, Guangjun; Sun, Yunfei; Sun, J. Chemical Composition of Clarified Bayberry ( Myrica rubra Sieb . et Zucc .) Juice Sediment. *J. Agric. Food Chem.* **54**, 7710–7716 (2006).

21. Zomorodian, K. *et al.* Chemical Composition and Antimicrobial Activities of Essential Oils from Nepeta cataria L. against Common Causes of Food-Borne Infections . *ISRN Pharm.* **2012**, 1–6 (2012).

22. Gilani, A. H. *et al.* Chemical composition and mechanisms underlying the spasmolytic and bronchodilatory properties of the essential oil of Nepeta cataria L. *J. Ethnopharmacol.* **121**, 405–411 (2009).

23. Simon Distingusihed Professor of Plant Biology, J. Phytochemical Analysis and Anti-Inflammatory Activity of Nepeta cataria Accessions. *J. Med. Act. Plants* **7**, 19 (2018).

24. Won, H. S. & Hyung, H. B. Identification of characteristic aroma-active compounds from water dropwort (Oenanthe javanica DC.). *J. Agric. Food Chem.* **53**, 6766–6770 (2005).

25. He, S. *et al.* Hypoglycemic effects of phenolic compound-rich aqueous extract from water dropwort (: Oenanthe javanica DC.) on streptozotocin-induced diabetic mice. *New J. Chem.* **44**, 5190–5200 (2020).

26. Chan, E. W. C., Wong, S. K. & Chan, H. T. Ulam herbs of Oenanthe javanica and Cosmos caudatus: An overview on their medicinal properties. *J. Nat. Remedies* **16**, 137–147 (2016).

27. Lu, C. L. & Li, X. F. A Review of Oenanthe javanica (Blume) DC. as Traditional Medicinal Plant and Its Therapeutic Potential. *Evidence-based Complement. Altern. Med.* **2019**, 17–26 (2019).

28. Böhm, H. “Opuntia dillenii” – An Interesting and Promising Cactaceae Taxon. *J. Prof. Assoc. Cactus Dev.* **10**, 148–170 (2008).

29. Betancourt, C., Cejudo-bastante, M. J., Francisco, J. & Hurtado, N. Pigment composition and antioxidant capacity of betacyanins and betaxanthins fractions of Opuntia dillenii (Ker Gawl) Haw cactus fruit. *Food Res. Int.* **101**, 173–179 (2017).

30. Sharma, C., Rani, S., Kumar, B., Kumar, A. & Raj, V. Plant Opuntia dillenii: A Review on It’s Traditional Uses, Phytochemical and Pharmacological Properties. *EC Pharm. Sci.* **1**, 29–43 (2015).

31. Deka, D. C. *et al.* Oroxylum indicum- a medicinal plant of North East India: An overview of its nutritional, remedial, and prophylactic properties. *J. Appl. Pharm. Sci.* **3**, 104–112 (2013).

32. Begum, M. *et al.* Ethnopharmacological inspections of organic extract of oroxylum indicum in rat models: A promising natural gift. *Evidence-based Complement. Altern. Med.* **2019**, (2019).

33. Dinda, B., Silsarma, I., Dinda, M. & Rudrapaul, P. Oroxylum indicum (L.) Kurz, an important Asian traditional medicine: From traditional uses to scientific data for its commercial exploitation. *J. Ethnopharmacol.* **161**, 255–278 (2015).

34. Luitel, H. N. *et al.* Chemical constituents from *Oroxylum indicum* (L.) Kurz of Nepalese Origin. *Sci. World* **8**, 66–68 (1970).

35. Merugu, S., Swetha, T. & B., V. PHYTOCHEMISTRY AND PHARMACOLOGY OF OXALIS CORNICULATA LINN.: A REVIEW. *Int. J. Pharm. Sci. Res.* **3**, 4634–4639 (2012).

36. Ibrahim, M. *et al.* Corniculatin A, a new flavonoidal glucoside from Oxalis corniculata. *Brazilian J. Pharmacogn.* **23**, 630–634 (2013).

37. Soni*, R. K., Irchhaiya, R., Dixit, V. & Alok, S. Department of Pharmacognosy, Institute of Pharmacy, Bundelkhand University, Jhansi, Uttar Pradesh, India. *Ijpsr* **4**, 4525–4530 (2013).

38. Wang, L., Jiang, Y., Han, T., Zheng, C. & Qin, L. A phytochemical, pharmacological and clinical profile of Paederia foetida and P. scandens. *Nat. Prod. Commun.* **9**, 879–886 (2014).

39. Zhou, Y. *et al.* Chemical constituents, antibacterial activity and mechanism of Paeonia suffruticosa Andr. buds extract against Staphylococcus aureus and Escherichia coli O157:H7 . *Nat. Prod. Res.* **0**, 1–5 (2019).

40. Pan, Y., Gao, Z., Huang, X. Y., Chen, J. J. & Geng, C. A. Chemical and biological comparison of different parts of Paeonia suffruticosa (Mudan) based on LCMS-IT-TOF and multi-evaluation in vitro. *Ind. Crops Prod.* **144**, 112028 (2020).

41. Lei, G., Song, C. & Luo, Y. Chemical composition of hydrosol volatiles of flowers from ten Paeonia × suffruticosa Andr. cultivars from Luoyang, China. *Nat. Prod. Res.* (2020) doi:10.1080/14786419.2019.1709192.

42. Tanaka, O. *et al.* Saponins of plants of Panax species collected in Central Nepal, and their chemotaxonomical significance. III. *Chem. Pharm. Bull.* **48**, 889–892 (2000).

43. Sharma, P. & Sett, R. Micropropagation of Indian Ginseng ( Panax pseudoginseng Wall): A Proposition to Save an Endangered Commercial and Medicinal Forest Plant . *J. Hum. Ecol.* **12**, 201–205 (2001).

44. Masihuddin, M., Jafri, M., Siddiqui, A. & Chaudhary, S. Traditional Uses, Phytochemistry and Pharmacological Activities of Papaver Somniferum With Special Reference of Unani Medicine an Updated Review. *J. Drug Deliv. Ther.* **8**, 110–114 (2018).

45. Gilpin, R. K. & Hann, C. J. Alkaloids, Pharmaceutical Analysis of. *Encycl. Anal. Chem.* 1–20 (2006) doi:10.1002/9780470027318.a1902.

46. Morimoto, S., Suemori, K., Taura, F. & Shoyama, Y. New dimeric morphine from opium poppy (Papaver somuniferum) and its physiological function. *J. Nat. Prod.* **66**, 987–989 (2003).

47. Bulduk, I. & Taktak, F. Isolation and characterization of antitumor alkaloid from poppy capsules (Papaver somniferum). *J. Chem.* **2013**, (2013).

48. Payum, T. Phytocomposition and pharmacological importance of Paris polyphylla (Smith.) and needs of its conservation in Arunachal Pradesh, India. *Arch. Agric. Environ. Sci.* **3**, 143–150 (2018).

49. Sharma, A., Kalita, P. & Tag, H. Distribution and phytomedicinal aspects of Paris polyphylla Smith from the Eastern Himalayan Region: A review. *Tang [Humanitas Med.* **5**, 15.1-15.12 (2015).

50. Jing, S., Wang, Y., Li, X., Man, S. & Gao, W. Chemical constituents and antitumor activity from Paris polyphylla Smith var. yunnanensis. *Nat. Prod. Res.* **31**, 660–666 (2017).

51. Al-Madhagi, W. M. *et al.* Chemical profiling and biological activity of Peperomia blanda (Jacq.) Kunth. *PeerJ* **2018**, 1–19 (2018).

52. Velozo, L. S. M. *et al.* Unusual chromenes from Peperomia blanda. *Phytochemistry* **67**, 492–496 (2005).

53. Depeursinge, A. *et al.* Fusing Visual and Clinical Information for Lung Tissue Classification in HRCT Data. *Artif. Intell. Med.* 1118 (2010) doi:10.1016/j.

54. Youfang Ding. Characterization of fatty acid composition from five perilla seed oils in China and its relationship to annual growth temperature. *J. Med. Plants Res.* **6**, 1645–1651 (2012).

55. Guan, Z. *et al.* Identification and quantitation of phenolic compounds from the seed and pomace of perilla frutescens using HPLC/PDA and HPLC-ESI/QTOF/MS/MS. *Phytochem. Anal.* **25**, 508–513 (2014).

56. Huang, M., Shen, S., Luo, C. & Ren, Y. Genus Periploca (Apocynaceae): A Review of Its Classification, Phytochemistry, Biological Activities and Toxicology. *Molecules* **24**, (2019).

57. Gaire, B. P. & Subedi, L. Phytochemistry, pharmacology and medicinal properties of Phyllanthus emblica Linn. *Chin. J. Integr. Med.* (2014) doi:10.1007/s11655-014-1984-2.

58. Asmilia, N., Fahrimal, Y., Abrar, M. & Rinidar, R. Chemical Compounds of Malacca Leaf (Phyllanthus emblica) after Triple Extraction with N-Hexane, Ethyl Acetate, and Ethanol. *Sci. World J.* **2020**, (2020).

59. Yang, F. *et al.* Chemical constituents from the fruits of Phyllanthus emblica L. *Biochem. Syst. Ecol.* **92**, (2020).

60. Shimoda, K. *et al.* Glycosylation of stilbene compounds by cultured plant cells. *Molecules* **25**, 2–9 (2020).

61. Tang, W. & Eisenbrand, G. Phytolacca americana L. and P. acinosa Roxb. *Springer, Berlin, Heidelb.* **2**, 765–775 (1992).

62. Chang, X. & Kang, W. Antioxidant and a-glucosidase inhibitory compounds from Pimpinella candolleana wight et arn. *Med. Chem. Res.* **21**, 3–8 (2012).

63. Wu, Y. Y. *et al.* Chemical constituents from the tubers of Pinellia ternata (Araceae) and their chemotaxonomic interest. *Biochem. Syst. Ecol.* **62**, 236–240 (2015).

64. Lin, S. *et al.* Pinellia ternata (Thunb.) Makino Preparation promotes sleep by increasing REM sleep. *Nat. Prod. Res.* **33**, 1–4 (2018).

65. Kil, Y. S., Kang, U., Nam, J. W., Hong, J. & Seo, E. K. Phytochemical Study of the Low Polar Constituents of Pinellia ternata. *Chem. Nat. Compd.* **53**, 1152–1153 (2017).

66. Sa, N. H., Tam, N. T., Thi, N., Anh, H. & Quan, T. D. Abietane diterpenoids and neolignans from the roots of Pinus kesiya. *Vietnam J. Chem.* **55**, 240–243 (2017).

67. Ragasa, C. Y., Tan, M. C. S., Linis, V. C. & Shen, C. C. A triterpene and a depside from Parmotrema austrocetratum Elix and J. Johnst. *Pharmacogn. J.* **10**, S27–S29 (2018).

68. Weerapreeyakul, N., Machana, S. & Barusrux, S. Synergistic effects of melphalan and Pinus kesiya Royle ex Gordon (Simaosong) extracts on apoptosis induction in human cancer cells. *Chinese Med. (United Kingdom)* **11**, 1–10 (2016).

69. Zhou, Q. *et al.* *Identification and quantification of phytochemical composition and anti-inflammatory, cellular antioxidant, and radical scavenging activities of 12 Plantago species*. *Journal of Agricultural and Food Chemistry* vol. 61 (2013).

70. Hieu, L. D., Thang, T. D., Hoi, T. M. & Ogunwande, I. A. Chemical composition of essential oils from four Vietnamese species of Piper (Piperaceae). *J. Oleo Sci.* **63**, 211–217 (2014).

71. Liu, T. *et al.* Chemical constituents from Piper boehmeriifolium (Miq.) Wall. ex C. DC. *Biochem. Syst. Ecol.* **75**, 27–30 (2017).

72. Mgbeahuruike, E. E., Yrjönen, T., Vuorela, H. & Holm, Y. Bioactive compounds from medicinal plants: Focus on Piper species. *South African J. Bot.* **112**, 54–69 (2017).

73. Tang, G. H. *et al.* Cytotoxic amide alkaloids from piper boehmeriaefolium. *J. Nat. Prod.* **74**, 45–49 (2011).
