# Supplementary material for: Plant-derived compounds effectively inhibit the main protease of SARS-CoV-2: An in silico approach
Source: PLoS One. 2022 Aug 23;17(8):e0273341. doi: 10.1371/journal.pone.0273341 (PMC9398018; doi:10.1371/journal.pone.0273341)
Supplement: S3 Table — (DOCX) [file pone.0273341.s003.docx]

S3 Table: Chemical name and pubchem CID of different phytochemicals retrived from different plants.

| **SERIAL NO.** | **PLANT NAME** | **CHEMICAL NAME** | **PUBCHEM CID** |
| --- | --- | --- | --- |
| 01 | *Piper nigrum* Linn. | α-phellandrene^1^ | 7460 |
|  |  | Camphene^1^ | 6616 |
|  |  | Sabinene^1^ | 18818 |
|  |  | β-pinene^1^ | 14896 |
|  |  | α-pinene^1^ | 6654 |
|  |  | 3-Carene^1^ | 26049 |
|  |  | Trifluoromethanesulfenyl fluoride^1^ | 550355 |
|  |  | (+)-Camphene^1^ | 92221 |
|  |  | Limonene^1^ | 22311 |
|  |  | β-phellandrene^1^ | 11142 |
|  |  | Gamma-terpinene^1^ | 7461 |
|  |  | Terpinolene^1^ | 11463 |
|  |  | Linalol^1^ | 6549 |
|  |  | 4-terpineol^1^ | 11230 |
|  |  | Delta-Elemene^1^ | 12309449 |
|  |  | Copaene^1^ | 12303902 |
|  |  | β-Elemene^1^ | 6918391 |
|  |  | α-Bergamotene^1^ | 86608 |
|  |  | Caryophyllene^1^ | 5281515 |
|  |  | α-Curcumene^1^ | 92139 |
|  |  | Cedrene^1^ | 521207 |
|  |  | β-Bisabolene^1^ | 10104370 |
|  |  | (+)-delta-Cadinene^1^ | 441005 |
|  |  | Caryophyllene oxide^1^ | 1742210 |
|  |  | Spathulenol^1^ | 92231 |
|  |  | α-Bisabolol^1^ | 1549992 |
|  |  | 2-Undecanone^1^ | 8163 |
|  |  | Lauric acid^2^ | 3893 |
|  |  | Myristic acid^2^ | 11005 |
|  |  | Palmitoleic acid^2^ | 445638 |
|  |  | Palmitic acid^2^ | 985 |
|  |  | Oleic acid^2^ | 445639 |
|  |  | Stearic acid^2^ | 5281 |
|  |  | Lignoceric acid^2^ | 11197 |
|  |  | Cyclohexene, 4-ethenyl-4-methyl-3-(1-methylethenyl)-1-(1-methylethyl)-, (3R-trans)-^3^ | 89316 |
|  |  | alpha-Copaene^3^ | 70678558 |
|  |  | 2-Methylene-4,8,8-trimethyl-4-vinyl-bicyclo[5.2.0]nonane^3^ | 564746 |
|  |  | 1,4,7,-Cycloundecatriene, 1,5,9,9-tetramethyl-, Z,Z,Z-^3^ | 5368784 |
|  |  | 1-Piperidinecarboxaldehyde^3^ | 17429 |
|  |  | Ethyl 6,9,12-hexadecatrienoate^3^ | 91697553 |
|  |  | 1,3,3-trimethyl-2-Oxabicyclo[2.2.2]octan-6-ol^3^ | 529885 |
|  |  | Hexadecanoic acid, methyl ester^3^ | 8181 |
|  |  | Piperonal^3^ | 8438 |
|  |  | 1H-Cycloprop[e]azulen-7-ol, decahydro-1,1,7-trimethyl-4-  methylene-, [1ar-(1a.alpha.,4a.alpha.,7.beta.,7a.beta.,7b.alpha.)]-^3^ | 6432640 |
|  |  | Tricyclo[5.2.2.0(1,6)]undecan-3-ol, 2-methylene-6,8,8-trimethyl-^3^ | 535346 |
|  |  | (+-)-1-Isopropylcyclopropane-trans-1,cis-2-dicarboxylic acid^3^ | 567821 |
|  |  | Octadecanoic acid, ethyl ester^3^ | 8122 |
|  |  | Ethyl oleate^3^ | 5363269 |
|  |  | Ethyl 9.cis.,11.trans.-octadecadienoate^3^ | 9963693 |
|  |  | Vanillin lactoside^3^ | 592475 |
|  |  | n-Tetracosanol-1^3^ | 10472 |
|  |  | Ethyl 9,12,15-octadecatrienoate^3^ | 5367460 |
|  |  | Phytol^3^ | 5280435 |
|  |  | 1H-Cycloprop[e]azulen-7-ol, decahydro-1,1,7-trimethyl-4-methylene-, [1ar-(1a.alpha.,4a.alpha.,7.beta.,7a.beta.,7b.alpha.)]-^3^ | 6432640 |
|  |  | n-Hexadecanoic acid^3^ | 985 |
|  |  | Naphthalene, decahydro-2,2-dimethyl^3^ | 591982 |
|  |  | (8R,Z)-8-Methyl-6-((R)-2-methylpentylidene)octahydroindolizine^3^ | 91721008 |
|  |  | 2-Ethyl-5-undecyl-.delta.1--pyrroline^3^ | 566328 |
|  |  | 6-Octadecenoic acid^3^ | 5282754 |
|  |  | 9,12-Octadecadienoic acid (Z,Z)-^3^ | 5280450 |
|  |  | (3S,5R,7aS)-3-(But-3-en-1-yl)-5-(hex-5-en-1-yl)hexahydro-1H-pyrrolizine^3^ | 91724130 |
|  |  | Piperidine, 1-(1-oxo-3-phenyl-2-propenyl)-^3^ | 223147 |
|  |  | 2-Cyclohexen-3-ol-1-one, 2-[1-iminotetradecyl]-^3^ | 135703373 |
|  |  | Isonipecotic acid, N-acryloyl-, undecyl ester^3^ | 91740764 |
|  |  | 4-Hexadecenoic acid, pyrrolidide^3^ | 91705225 |
|  |  | cis-13-Octadecenoic acid, 4,4-dimethyloxazoline derivative^3^ | 91705087 |
|  |  | trans-2-Octadecenoic acid^3^ | 5282750 |
|  |  | 13-Eicosenoic acid, pyrrolidide^3^ | 91705100 |
|  |  | 4,5,6,7-Tetrahydrobenz[z]isoxazole-5-ol-4-one, 3-[9-tridecenyl]-^3^ | 5364480 |
|  |  | 10,13-Octadecadienoic acid^3^ | 54284936 |
|  |  | Pipercallosine^4^ | 5372201 |
|  |  | Tricholein^4^ | 21580214 |
|  |  | Trichostachine^4^ | 636537 |
|  |  | Piperine^4^ | 638024 |
|  |  | 3’ ,4’ -methylenedioxycinnamaldehyde^4^ | 84630 |
|  |  | Retrofractamide A^4^ | 11012859 |
|  |  | Retrofractamide D^4^ | 131751424 |
|  |  | Bicyclo[7.2.0]undec -4-ene,4,11,11 -trimethyl -8-methylene -,[1R-(4E,9S)]^4^ | 6887 |
|  |  | Octadecanoic acid^4^ | 5281 |
|  |  | p-Cymene^5^ | 7463 |
|  |  | α-Copaene^5^ | 19725 |
|  |  | α-Cubebene^5^ | 86609 |
|  |  | α-Humulene^5^ | 5281520 |
|  |  | β-Caryophyllene^5^ | 5281515 |
|  |  | β-Myrcene^5^ | 31253 |
|  |  | δ-3-Carene^5^ | 26049 |
|  |  | δ-Elemene^5^ | 12309449 |
|  |  | (E)-Nerolidol^5^ | 5284507 |
|  |  | 6-Hydroxypiperitol^5^ | 10317157 |
|  |  | ar-Turmerone^5^ | 160512 |
|  |  | Caryophyllenol^5^ | 61125 |
|  |  | Eugenol^5^ | 3314 |
|  |  | Humulene epoxide II^5^ | 10704181 |
|  |  | Isocaryophyllene oxide^5^ | 1742211 |
|  |  | Isospathulenol^5^ | 14038848 |
|  |  | Myrtenol^5^ | 10582 |
|  |  | p-Cymen-8-ol^5^ | 14529 |
|  |  | Piperitenone oxide^5^ | 61942 |
|  |  | trans-Sabinol^5^ | 6429076 |
|  |  | Verbenone^5^ | 29025 |
|  |  | α-Selinene^5^ | 10856614 |
|  |  | α-Terpinene^5^ | 7462 |
|  |  | β-Eudesmol^5^ | 91457 |
|  |  | β-Selinene^5^ | 442393 |
|  |  | Caryophylla-4(12),8(13)-dien-5β-ol^5^ | 91753606 |
|  |  | γ-Selinene^5^ | 521334 |
|  |  | Guaiol^6^ | 227829 |
|  |  | Piperanine^6^ | 5320618 |
|  |  | Piperolein A^6^ | 11141599 |
|  |  | Piperolein B^6^ | 21580213 |
|  |  | Piperyline^6^ | 636537 |
|  |  | Pellitorine^6^ | 5318516 |
|  |  | Hexadecanoylpyrrolidine^6^ | 247220 |
| 02 | *Plumbago zeylanica* Linn. | Trans-cinnamic acid^7^ | 444539 |
|  |  | Isoshinanolone^7^ | 443777 |
|  |  | Indole-3-carboxaldehyde^7^ | 10256 |
|  |  | Vanillic acid^7^ | 8468 |
|  |  | Napthoquinone^7^ | 8530 |
|  |  | Plumbagin^7^ | 10205 |
|  |  | Chloroplumbagin^7^ | 338719 |
|  |  | Maritinone^7^ | 633024 |
|  |  | Elliptinone^7^ | 146680 |
|  |  | Isoshinanolone^7^ | 443777 |
|  |  | Lapachol^7^ | 3884 |
|  |  | Seselin^7^ | 68229 |
|  |  | Suberosin^7^ | 68486 |
|  |  | Zeylanone^7^ | 5276618 |
|  |  | Sitosterol^7^ | 222284 |
|  |  | Isozeylanone^7^ | 100947536 |
|  |  | Glucopyranoside^7^ | 5793 |
|  |  | n-hexadecanoic acid^8^ | 985 |
|  |  | Naphtho(2,3-b)furan-2 (3H)-one^8^ | 85823872 |
|  |  | Oleic Acid^8^ | 445639 |
|  |  | Binaphthoquinone^9^ | 628770 |
|  |  | 3,3′-Biplumbagin^9^ | 183757 |
|  |  | 1-Naphthol^9^ | 7005 |
|  |  | 3- chloroplumbagin^9^ | 338719 |
|  |  | Droserone^9^ | 442739 |
|  |  | Plumbagic acid^9^ | 92468470 |
|  |  | Plumbazeylanone^9^ | 100947539 |
|  |  | Hentriacontane^9^ | 12410 |
|  |  | Campesterol^10^ | 173183 |
|  |  | Stigmasterol^10^ | 5280794 |
|  |  | 2,5-dimethyl-7 hydroxy chromone^10^ | 5316891 |
|  |  | 4-hydroxybenzaldehyde^10^ | 126 |

REFERENCES

1. Morshed, S., Hossain, M. D., Ahmad, M. & Junayed, M. Physicochemical characteristics of essential oil of black pepper (Piper nigrum) cultivated in Chittagong, Bangladesh. *J. Food Qual. Hazards Control* **4**, 66–69 (2017).

2. Hossain, M. D. *et al.* Studies on Fatty Acids Composition and Some Valuable Nutrients of Piper nigrum Linn. (Gol Morich). *Dhaka Univ. J. Sci.* **62**, 65–68 (2015).

3. Chen, W., Zou, L., Chen, W., Hu, Y. & Chen, H. Effects of Black Pepper (Piper nigrum L.) Chloroform Extract on the Enzymatic Activity and Metabolism of Escherichia coli and Staphylococcus aureus. *J. Food Qual.* **2018**, (2018).

4. Siddiqui, B. S., Gulzar, T., Begum, S., Afshan, F. & Sattar, F. A. Insecticidal amides from fruits of Piper nigrum Linn. *Nat. Prod. Res.* **19**, 143–150 (2005).

5. Salehi, B. *et al.* *Piper species: A comprehensive review on their phytochemistry, biological activities and applications*. *Molecules* vol. 24 (2019).

6. Takooree, H. *et al.* A systematic review on black pepper (Piper nigrum L.): from folk uses to pharmacological applications. *Crit. Rev. Food Sci. Nutr.* **59**, S210–S243 (2019).

7. Tyagi, R. & Menghani, E. A Review on Plumabgo zeylanica : A Compelling Herb. *Int. J. Pharma Sicences Res.* **5**, 119–126 (2014).

8. Rajakrishnan, R. *et al.* Phytochemical evaluation of roots of Plumbago zeylanica L. and assessment of its potential as a nephroprotective agent. *Saudi J. Biol. Sci.* **24**, 760–766 (2017).

9. Mandavkar, Y. D. & Jalalpure, S. S. A comprehensive review on Plumbago zeylanica linn. *African J. Pharm. Pharmacol.* **5**, 2738–2747 (2011).

10. Pant, M., Lal, A., Rana, S. & Rani, A. Plumbago Zeylanica L.: a Mini Review. *Int. J. Pharm. Appl.* **3**, 399–405 (2012).
